# Isolation Techniques of Micro/Nano‐Scaled Species for Biomedical Applications

**DOI:** 10.1002/advs.202414109

**Published:** 2025-05-24

**Authors:** Qing Lu, Zhinan Zhang, Xianting Ding

**Affiliations:** ^1^ State Key Laboratory of Mechanical System and Vibration Shanghai Jiao Tong University Shanghai 200240 China; ^2^ School of Mechanical Engineering Shanghai Jiao Tong University Shanghai 200240 China; ^3^ Department of Anesthesiology and Surgical Intensive Care Unit Xinhua Hospital School of Medicine and School of Biomedical Engineering Shanghai Jiao Tong University Shanghai 200030 China; ^4^ State Key Laboratory of Systems Medicine for Cancer Institute for Personalized Medicine Shanghai Jiao Tong University Shanghai 200030 China

**Keywords:** biomedical application, isolation, micro/nano‐scaled species, microfluidics

## Abstract

Isolation of micro/nano‐scaled bioparticles, such as circulating tumor cells (CTCs), exosomes, bacteria, white blood cells (WBCs), platelets, and viruses, from the sample is essential for cancer diagnosis and treatment, preventing bacterial infections, and monitoring human health. Numerous separation techniques, including magnetophoresis, dielectrophoresis, acoustophoresis, optophoresis, and fluorescence‐activated sorting (FAS) have been developed to isolate the target bioparticles from complex samples accurately. However, these active methods usually rely on sophisticated instruments which are expensive and bulky. Passive platforms with high throughput, low cost, and small volume have gradually become alternative methods. Alongside this context, this review paper is no longer confined to one specific category of isolation techniques, advanced systems that have been developed in recent years are comprehensively introduced. Characteristics and limitations of each technology are discussed according to the critical performance parameters including purity, recovery rate, throughput, resolution, size, and convenience. Specific biomedical applications of separation techniques are summarized to provide practical implications for disease diagnosis, treatment, and mechanism research. This review also addresses the current challenges, potential solutions, and prospects in this field, laying the foundation for further optimization, innovation, and cross‐integration of isolation techniques in the future.

## Graphical abstract

Separating micro/nano‐scale bioparticles from samples is essential for disease diagnosis, prevention, and monitoring. Therefore, a comprehensive overview of the latest advanced separation technologies is provided, and characteristics and limitations based on six key performance parameters, including purity, recovery rate, throughput, resolution, size, and convenience are discussed. Furthermore, biomedical applications, challenges, potential solutions, and future prospects of isolation techniques are also explored.

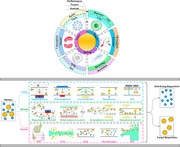

## 1 Introduction

Isolation of micro/nano‐scaled bioparticles, such as CTCs (15 µm–22 µm),^[^ ^1^, ^2^ ^]^ exosomes (30–150 nm),^[^ ^3^, ^4^, ^5^ ^]^ bacteria (≈1 µm),^[^ ^6^, ^7^ ^]^ WBCs (≈9 µm),^[^ ^8^, ^9^ ^]^ platelets (≈3.5 µm),^[^ ^10^, ^11^ ^]^ and viruses (30–150 nm),^[^ ^12^, ^13^ ^]^ holds significant potential across various research fields, including early tumor diagnosis, prognosis, therapy, metastasis mechanism studies, prevention of bacterial infections, and health monitoring.^[^ ^14^, ^15^, ^16^, ^17^, ^18^ ^]^ These bioparticles contain various biological components, such as proteins, DNA, and RNA, which perform complex biological functions and collectively maintain normal physiological activities.^[^ ^19^, ^20^ ^]^ Biomedical researches and applications typically focus on specific bioparticle types in the complex sample which also contains other interfering components such as red blood cells (RBCs, 5 × 10^9^ RBCs/mL) and WBCs (1 × 10^7^ WBCs/mL) in human whole blood,^[^ ^21^, ^22^ ^]^ Therefore, target bioparticles are usually required to be precisely and rapidly isolated with other species from the original sample.^[^ ^2^, ^23^, ^24^ ^]^

Traditionally, three types of methods have been proposed for separating bioparticles: active, passive, and hybrid.^[^ ^25^, ^26^, ^27^, ^28^ ^]^ Active methods rely on external driving forces formed by multi‐physical fields, such as magnetic, electric, acoustic, and optical fields.^[^ ^29^, ^30^, ^31^, ^32^ ^]^ Separation is realized by distinct driving forces exerted on the bioparticle, typically depending on their physical characteristics such as size, shape, density, deformability, magnetism, dielectricity, and refractive index.^[^ ^33^, ^34^, ^35^, ^36^, ^37^, ^38^, ^39^ ^]^ Active separation methods generally provide accurate and on‐demand control of bioparticle position distribution, but mostly rely on sophisticated instrument architectures, which significantly increase the cost, operational complexity, and integration difficulty.^[^ ^40^, ^41^ ^]^ In addition, the high‐precision manipulation often comes at the cost of sacrificing sample processing throughput, making it difficult to quickly isolate target bioparticles with other components.^[^ ^42^ ^]^ As a result, the passive technique with high throughput, low cost, small size, and convenience has gradually become the other promising method to isolate bioparticles.^[^ ^43^, ^44^, ^45^ ^]^

Passive methods utilize intrinsic hydrodynamic forces, generated by the interactions between the bioparticles, fluid, and microstructures, to achieve isolation.^[^ ^46^, ^47^, ^48^ ^]^ Intrinsic hydrodynamic forces are primarily influenced by the physical properties of bioparticle, channel cross‐sectional shape and size, channel distribution, and properties of the fluid.^[^ ^49^, ^50^, ^51^, ^52^ ^]^ Target bioparticles and interfering species are subjected to distinct hydrodynamic forces when flowing in optimized channels due to differences in their physical properties. This enables the separation of bioparticles based on variations in migration speed or equilibrium position.^[^ ^53^, ^54^, ^55^ ^]^ Although passive separation systems are easy to operate and integrate, they primarily rely on hydrodynamic forces generated by fixed microstructures. As a result, passive devices are incapable of reliably shielding against the interference of other bioparticles and change sorting objects as needed, making it difficult to compete with active methods in terms of separation purity and technical flexibility. For example, if passive techniques are used to isolate target bioparticles, it is usually necessary to lyse RBCs and dilute the sample dozens of times before isolating to ensure the purity,^[^ ^40^, ^56^ ^]^ which leads to complex operations and the low throughput, even if the throughput of diluted sample can reach mL·min^−1^ level.^[^ ^44^ ^]^ In addition, passive methods also face the problems of severe blockage and high shear stress, posing significant challenges to the integrity and recovery rate of bioparticles.^[^ ^57^, ^58^ ^]^

Hybrid methods refer to connecting multiple typical subsystems with mixing, pre‐focusing, and isolating functions in series, or embedding active control components into aforementioned separation devices.^[^ ^59^, ^60^, ^61^, ^62^ ^]^ The original intention of hybrid systems is to combine advantages of active and passive methods. However, simply connecting multiple sub‐modules in series is usually incapable of improving critical performance parameters, because the optimal performance of each module can only be achieved under specific conditions which are usually not met simultaneously due to coupling constraints between sub‐modules. In addition, the drawbacks of each sub‐module may carry over to the cascade system, potentially amplifying certain weaknesses. Embedding active control elements into active/passive separation devices typically offers greater technical advantages compared to serial systems. However, this approach involves more complex fabrication and higher costs, posing challenges for clinical applications.

Over the past two decades, innovative active, passive, and hybrid techniques for isolating micro/nano‐scaled bioparticles have emerged, driven by the interdisciplinary integration of mechanics, chemistry, biology, materials science, and medicine. This progress has enabled the development of rapid and accurate bioparticle separation methods, as well as the integration of these microsystems with advanced analytical technologies, potentially revolutionizing bioparticle separation and analysis. Although several review articles have summarized the aforementioned developments,^[^ ^45^, ^63^, ^64^ ^]^ most of them focus on specific targets, such as nanoparticles, or specific techniques, including deterministic lateral displacement (DLD), acoustophoresis, or inertial microfluidics (IMF). As a result, due to the lack of integration and comparison of the applications and performances of different techniques, existing review articles are generally only beneficial to researchers studying particular isolation method. Therefore, in preparing this review, we no longer confine our manuscript to any one specific separation technology. Instead, a comprehensive overview of advanced systems is provided, detailing their basic constitutions, working principles, and technical characteristics. The advantages and limitations of each method are discussed according to the critical performance parameters, including purity, recovery rate, throughput, resolution, size, and convenience. In addition, the applications of isolation technology in CTCs, exosomes, bacteria, WBCs, platelets, and viruses are also introduced. Finally, we analyze the current challenges in biological particle separation, explore potential solutions, and discuss future prospects. Therefore, we believe that this review can provide readers with a comprehensive understanding of the fundamental principles, technical characteristics, research challenges, and application scenarios of various mainstream separation methods from a broader perspective.

## 2 Isolation Techniques

**Figure 1:**
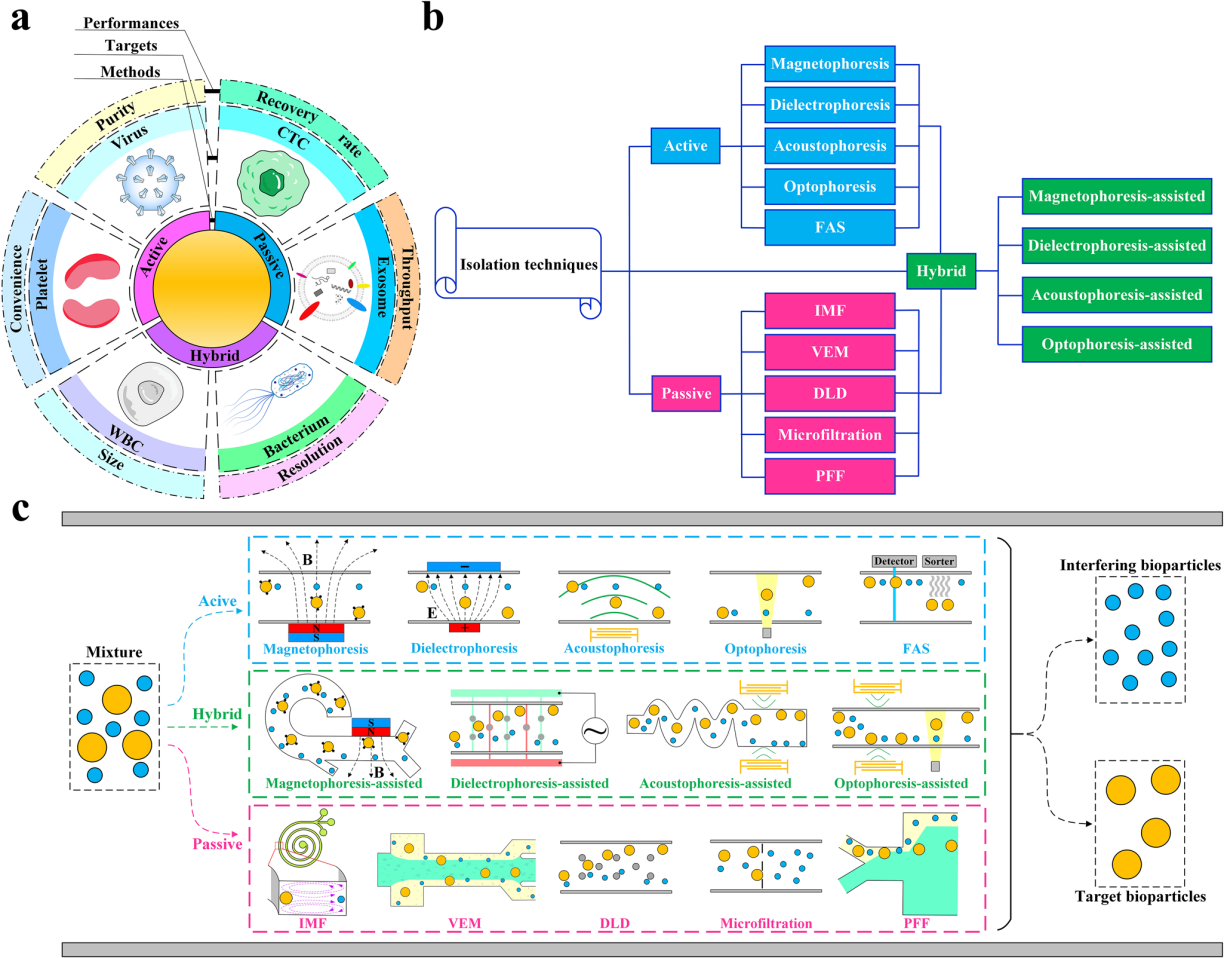
Overview of bioparticle isolation techniques. a) Methods, targets, and performances of isolation techniques. b) Isolation techniques are divided into active, passive, and hybrid methods. Active methods include magnetophoresis, dielectrophoresis, acoustophoresis, optophoresis, and fluorescence‐activated sorting (FAS). Passive methods include IMF, viscoelastic microfluidics (VEM), DLD, microfiltration, and pinched flow fraction (PFF). Hybrid methods refer to connecting multiple typical subsystems with mixing, pre‐focusing, and isolating functions in series or embedding active control components into aforementioned separation devices, including magnetophoresis‐assisted, dielectrophoresis‐assisted, acoustophoresis‐assisted, and optophoresis‐assisted techniques. c) Schematic illustration of various isolation techniques.

As illustrated in **Figure** **1**a, bioparticle separation techniques are classified as active, passive, and hybrid methods.^[^ ^65^ ^]^ Bioparticles are usually micro/nano‐scaled and include: CTCs, exosomes, bacteria, WBCs, platelets, and viruses.^[^ ^66^, ^67^, ^68^, ^69^, ^70^, ^71^ ^]^ In order to quantitatively evaluate separation performances of various techniques, six critical parameters are proposed, including purity, recovery rate, throughput, resolution, size, and convenience.^[^ ^56^, ^72^, ^73^ ^]^ In this section, we describe system architectures and working principles of each separation method, list some practical cases of sorting bioparticles, and point out advantages and limitations.

### 2.1 Active Methods

Active methods utilize external driving forces formed by multi‐physical fields, such as magnetic, electric, acoustic, and optical fields, to isolate bioparticles based on their distinct properties, including size, density, shape, deformability, magnetism, dielectricity, and refractive index. According to the type of external physical field and the working principle of each system, active separation technologies are divided into five categories: magnetophoresis, dielectrophoresis, acoustophoresis, optophoresis, and FAS.^[^ ^42^, ^74^, ^75^ ^]^

#### 2.1.1 Magnetophoresis

Magnetophoresis relies on the external magnetic field to directly or indirectly manipulate bioparticles, including positive and negative methods.^[^ ^76^, ^77^, ^78^ ^]^ Positive magnetophoresis refers to the phenomenon that bioparticles with greater magnetism than that of surrounding fluid media will migrate toward the field maxima when magnetized by non‐uniform external magnetic field,^[^ ^79^, ^80^ ^]^ and separate with other species based on the difference in magnetic forces. Positive magnetophoresis can directly manipulate magnetic micro/nano‐scaled particles or bioparticles with intrinsic magnetic properties, such as RBCs and magnetotactic bacteria.^[^ ^81^, ^82^ ^]^ However, most target bioparticles in practical applications such as CTCs are diamagnetic and cannot be directly manipulated by magnetic field.^[^ ^83^, ^84^ ^]^ In order to separate diamagnetic bioparticles by positive magnetophoresis, magnetic micro/nano‐scaled beads are usually labeled on surfaces of target bioparticles to modify their magnetic properties.^[^ ^85^, ^86^, ^87^ ^]^

**Figure 2:**
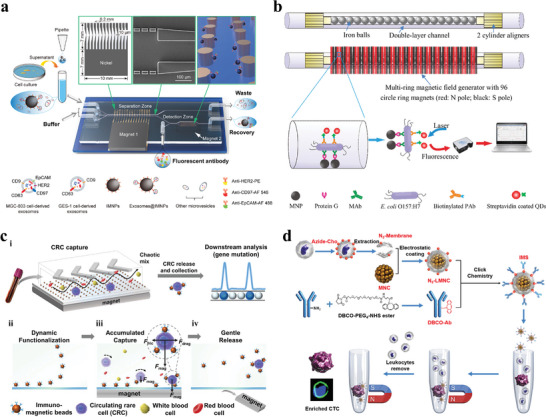
Positive magnetophoresis isolation techniques. a) An ExoSD chip for high‐purity immunomagnetic isolation and high‐sensitivity detection of exosomes derived by gastric cancer cells.^[^ ^88^ ^]^ Copyright 2021, Elsevier. b) Ultrasensitive fluorescent biosensor for high‐gradient magnetic isolation and rapid detection of foodborne pathogenic bacteria.^[^ ^89^ ^]^ Copyright 2018, Elsevier. c) DynarFace‐Chip for dynamical capture and reversible release of CTCs.^[^ ^85^ ^]^ Copyright 2021, Wiley. i) Schematic illustration of DynarFace‐Chip. ii) Functionalization of chip channel. iii) Cell capture. iv) Cell release. d) Construction of biomimetic immuno‐magnetosomes and the procedure of CTC enrichment.^[^ ^86^ ^]^ Copyright 2016, Wiley.

Yu et al.^[^ ^88^ ^]^ proposed an ExoSD chip to immunomagnetically separate exosomes from cell culture supernatants and detect cancer cell‐derived exosomes with high sensitivity (**Figure** **2**a). Comb‐like microstructures were introduced into separating zone to enhance the magnetic field gradient and magnetic flux density, improving magnetic forces exerted on particles. Microfilter structures consisting of two parallel rows of rectangular blocks were designed to improve the purity of exosomes by preventing impurities larger than 10 µm from entering the recovery stream due to random diffusion. The detection module composed of Ni cylindrical array was utilized to capture isolated particles, and exosomes were detected by injecting the fluorescent antibodies which can specifically bind to exosome surfaces. The separation purity and recovery rate of exosomes are more than 83% and 80%, respectively. In addition, the detection rate of ExoSD chip can achieve 70% which was verified by experimental results based on clinical serum samples from gastric cancer patients (stage I and II). Xue et al.^[^ ^89^ ^]^ investigated a fluorescent biosensor for specific separation and fast detection of *E. coli* O157:H7 (Figure 2b). The device is mainly composed of two parts including the multi‐ring magnetic field generator and the double‐layer channel consisting of inner and outer quartz capillaries. The inner quartz capillary was filled with 1 mm diameter iron balls which could be magnetized by external magnetic field formed through multi‐ring generator, producing high‐gradient magnetic fields. The protein G modified magnetic nanoparticles were first injected into the platform and captured uniformly in double‐layer channel by the high‐gradient magnetic field. The monoclonal antibodies against target bacteria *E. coli* O157:H7 were then infused, and immune magnetic nanoparticles were formed by binding the protein G to monoclonal antibodies specifically. The target bacteria were captured by immune magnetic nanoparticles from the impurities, constituting the magnetic bacteria complexes, and the quantum dots modified with the biotinylated anti‐*E. coli* O157:H7 polyclonal antibodies were injected to react with magnetic bacteria to form the fluorescent bacteria for rapid detection. Chen et al.^[^ ^85^ ^]^ developed a DynarFace‐Chip for dynamically capturing CTCs from blood sample (Figure 2c). The DynarFace, formed by attracting immunomagnetic beads using external magnets, can enhance the trapping force on CTCs by accumulating the attached immunomagnetic beads during interface collision. Consequently, CTCs were finally isolated and trapped on the DynarFace due to the progressively enhanced magnetic force. The release of CTCs was realized by magnet withdrawing‐induced DynarFace disassembly. This method overcomes the limitations of traditional affinity interfaces, which rely on immobilizing recognition components on a substrate. In addition, this technology no longer relies on the constant trapping force dependent on the density of recognition ligands and sample loading speed, and it enables the release of CTCs without introducing physicochemical stimuli. Xiong et al.^[^ ^86^ ^]^ synthesized biomimetic immuno‐magnetosomes with uniform size, high magnetization, and positive charge for isolating CTCs from WBCs (Figure 2d). The immuno‐magnetosomes were coated with membrane fragments of WBCs through electrostatic interaction, resulting in the repulsive interaction between immuno‐magnetosomes and WBCs due to their homology. As a result, the unspecific adsorption of WBCs was significantly inhibited, thereby lowering the background impurities.

Negative magnetophoresis is a label‐free separation technique, as it directly manipulates the magnetic fluid medium (ferrofluid or paramagnetic salt solution).^[^ ^84^, ^90^, ^91^ ^]^ When exposed to a non‐uniform external magnetic field, the magnetic fluid medium migrates toward the region with maximum magnetic field intensity, causing bioparticles to be pushed back by surrounding fluids in the opposite direction.^[^ ^92^, ^93^ ^]^ The magnetic buoyancy forces acting on the bioparticles are proportional to their volumes,^[^ ^94^, ^95^ ^]^ thereby causing the position difference between target bioparticles and interfering species after flowing through the non‐uniform magnetic field region.

**Figure 3:**
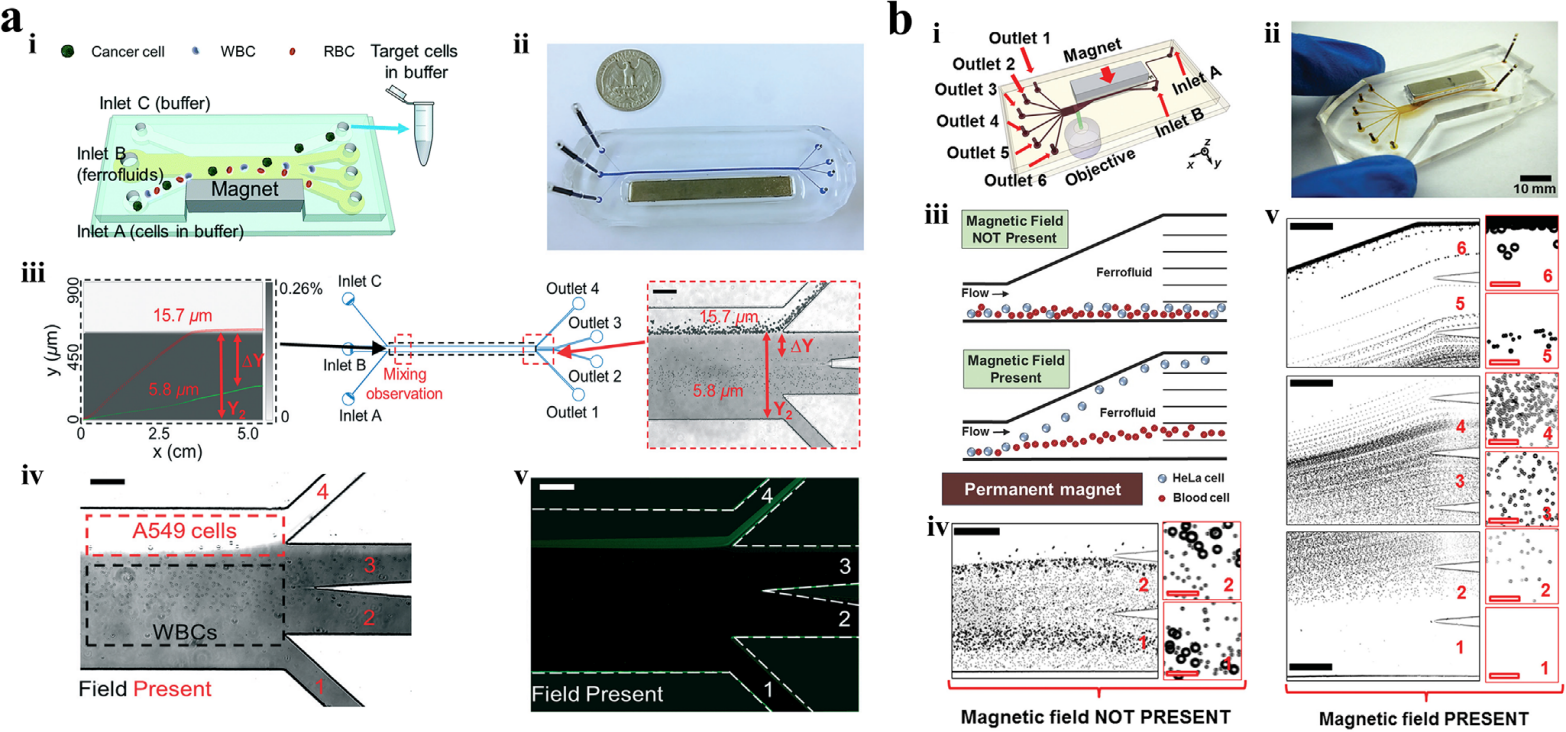
Negative magnetophoresis isolation techniques. a) Microfluidic chip capable of separating CTCs within a few seconds of ferrofluid exposure time.^[^ ^100^ ^]^ Copyright 2017, Royal Society of Chemistry. i) Schematic illustration of the device. ii) Prototype device. iii) The concentration profile of ferrofluids (gray scale), and the trajectories of particles with diameters of 5.8 µm (green circles) and 15.7µm (red circles). iv) Bright field image of the separation of WBCs and A549. v) Fluorescence image of A549. Scale bars: 200 µm. b) Microfluidic chip for separating HeLa cells with custom‐made biocompatible ferrofluid.^[^ ^97^ ^]^ Copyright 2015, Wiley. i) Device illustration. ii) Prototype device. iii) Schematic representation of separating mammalian cells in biocompatible ferrofluids. iv) In the absence of an external magnetic field, particle mixtures flowed out from outlet 1 – 2. Insets are zoom‐in views of outlets. v) When magnetic fields were applied, 15.8 µm microparticles exited through outlet 6, while 5.8 µm microparticles exited through outlet 2 – 5. Black solid scale bar: 400 µm, red hollow scale bars: 50 µm.

Frequently used negative magnetophoresis methods require exposing cells to ferrofluid during sample preparation and extraction, which can last for hours,^[^ ^96^, ^97^, ^98^ ^]^ inevitably causing particle endocytosis and diffusion and influencing the normal functions and viability of cells.^[^ ^99^ ^]^ Zhao et al.^[^ ^100^ ^]^ proposed a negative magnetophoresis device for separating tumor cells A549 from WBCs (**Figure** **3**a). The microfluidic device was specifically optimized to shorten the exposure time of live cells to ferrofluid from hours to seconds by integrating traditionally time‐consuming off‐chip sample preparation and extraction steps directly into the chip. Ferrofluids, cell samples, and buffer were injected into chip simultaneously, and the samples were mixed with ferrofluids almost instantaneously due to the strong magnetic convection. Cells separation experiments were conducted to verify the isolation performance of the system, and the experimental results indicated that the recovery rate and purity of CTCs were ≈80% and ≈30%, respectively. Light diffraction caused by the high concentration of magnetic nanoparticles makes it difficult to visualize bioparticles suspended in the medium. In order to address this problem, Zhao et al.^[^ ^97^ ^]^ developed an optimized negative magnetophoresis technique for isolating HeLa cells and blood cells, which combined the use of microfluidic chip with shallow channel (≈100 µm) and ferrofluids with low solid volume fraction (<1% v/v) (Figure 3b). In addition, a customized water‐based ferrofluid with pH 6.8, balanced salt concentration, and graft copolymer functionalized maghemite particles were utilized to maintain the viability of mouse blood cells and HeLa cells.

Positive magnetophoresis techniques enable accurate and high‐purity separation of target bioparticles by directly manipulating bioparticles labeled with magnetic beads. However, positive magnetophoresis typically requires incubating target bioparticles with magnetic micro/nano‐scaled beads for a certain period, followed by multiple washing steps.^[^ ^101^, ^102^, ^103^ ^]^ In addition, the effectiveness of positive magnetophoresis heavily depends on the number, size, and magnetism of magnetic beads,^[^ ^85^, ^104^ ^]^ and is therefore significantly influenced by endocytic capacities or ligand‐receptor interactions, which can vary even within the same type of bioparticles due to the heterogeneity.^[^ ^79^ ^]^ Therefore, positive magnetophoresis is usually time‐consuming and difficult to realize reliable and timely detection and analysis due to the requirement of incubating bioparticles and magnetic beads. Negative magnetophoresis caters the need for rapid analysis by eliminating labor‐intensive labeling steps through the incorporation of magnetic fluid media.^[^ ^97^ ^]^ Despite the progress of negative magnetophoresis, the method is limited by the difficulties of guaranteeing high purity, clearly visualizing separation process, and maintaining viability of bioparticles in magnetic fluid media.^[^ ^96^, ^97^, ^105^ ^]^ In addition, due to the reliance of external magnetic field, shielding devices need to be introduced during system integration and parallel design to minimize mutual coupling between magnetic sources, which increases the complexity, cost, and size of the system.

#### 2.1.2 Dielectrophoresis

Dielectrophoresis is the physical phenomenon that bioparticles migrate in the non‐uniform electric field under the action of dielectrophoresis force caused by different electrical properties from the surrounding fluid medium.^[^ ^106^, ^107^ ^]^ According to the polarization strength of bioparticles, dielectrophoresis responses are divided into two categories: positive and negative.^[^ ^108^, ^109^ ^]^ The positive/negative dielectrophoresis response occurs when the polarization of bioparticle is stronger/weaker than that of the fluid medium, making bioparticles migrate toward/away from the region with highest electric field intensity.^[^ ^110^, ^111^ ^]^ Bioparticle separation based on dielectrophoresis is achieved through distinct dielectrophoretic forces acting on different types of bioparticles, arising from variations in their physical properties such as size, shape, and electrical property.^[^ ^112^, ^113^ ^]^

**Figure 4:**
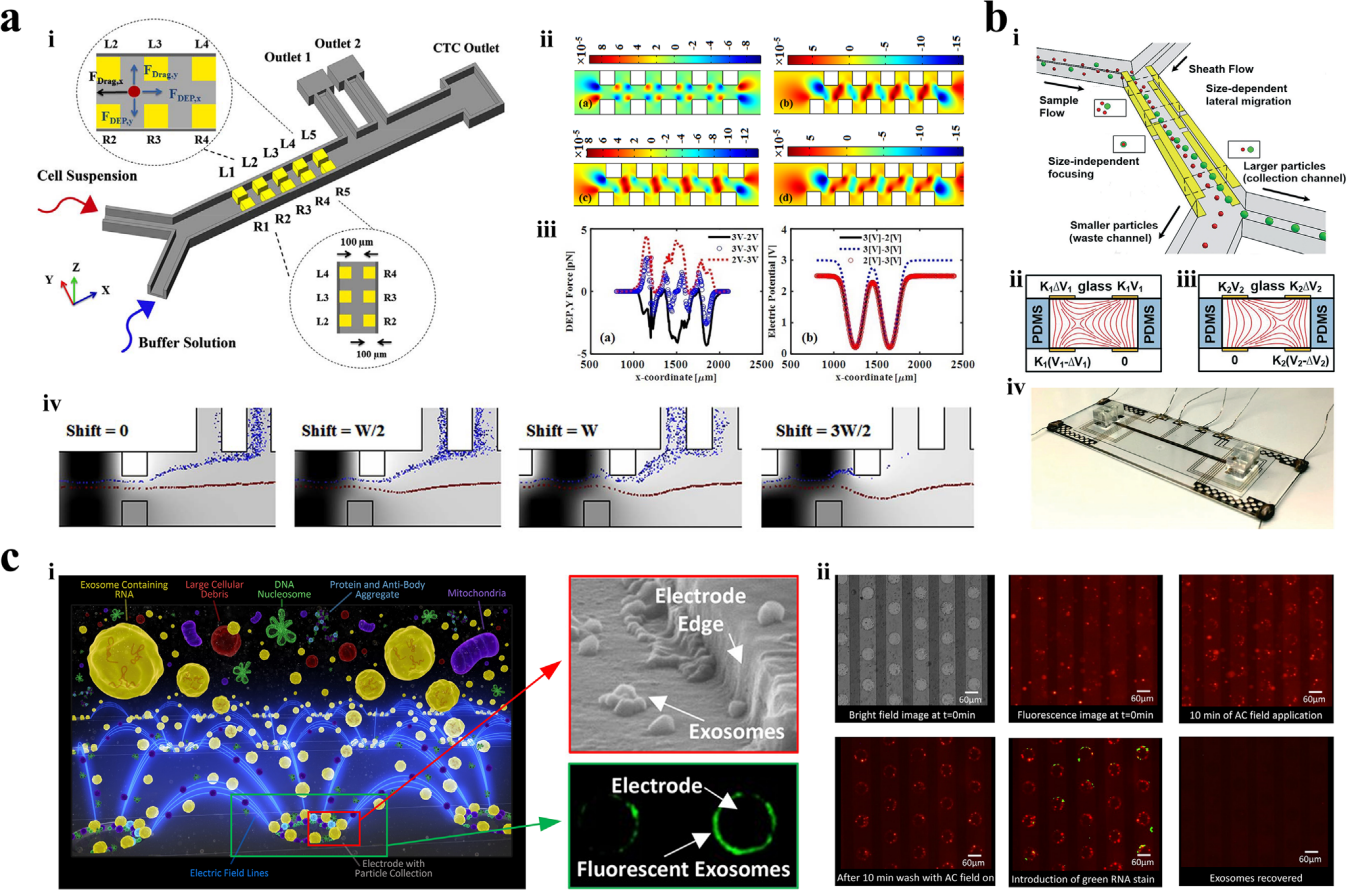
Dielectrophoresis isolation techniques. a) Dielectrophoresis‐based system equipped with differential electrodes.^[^ ^114^ ^]^ Copyright 2019, Institute of Electrical and Electronics Engineers. i) Schematic illustration of the platform. ii) Velocity distribution inside the microchannel. iii) The dielectrophoretic force and the electric potential in microchannel under different potentials applied on electrodes. iv) Separation results under different electrode shifts. Blue circle: blood cells. Red circle: MCF‐7. b) High‐resolution tunnel dielectrophoresis system.^[^ ^115^ ^]^ Copyright 2021, Royal Society of Chemistry. i) Schematic representation of the separation system. ii) Electric field in microchannel when alternating current signals were applied. iii) Prototype device. c) The alternating current electrokinetic microarray chip to separate and recovery glioblastoma exosomes from human plasma samples.^[^ ^116^ ^]^ Copyright 2017, American Chemical Society. i) Schematic diagram of the concentration process of exosomes and other nanoparticles. ii) Bright‐field and fluorescence images of exosome isolation at the different stages.

Shirmohammadli et al.^[^ ^114^ ^]^ proposed a microfluidic system based on direct current dielectrophoresis for isolating CTCs from blood cells with a purity of 83% and a recovery rate of 100% (**Figure** **4**a). Differential sidewall electrodes were designed in channels of chip to effectively reduce voltages, thereby weakening the joule heating effect. In addition, these electrodes guided cell movement by introducing hydrodynamic forces. Kung et al.^[^ ^115^ ^]^ developed a high‐resolution tunnel dielectrophoresis technique for isolating microparticles with similar dimensions, such as separating leukocyte subtype from peripheral blood mononuclear cells (Figure 4b). Two sets of alternating current signals were applied to quadro‐electrode pairs to form a specific electric field pattern. Electrode pairs near inlets were used to realize the size‐independent single‐stream focusing, while other electrode pairs facilitated size‐dependent lateral migration of microparticles. This microsystem is able to separate polystyrene particles with a size difference of 1 µm with a purity of 90%. Additionally, it enables low‐pass, high‐pass, and band‐pass filtering of monocellular mammalian cell population. Ibsen et al.^[^ ^116^ ^]^ investigated an alternating current electrokinetic microarray chip for separating and recovering glioblastoma exosomes from human plasma samples (Figure 4c). Due to the dielectric property difference of various bioparticles, nanoparticles such as exosomes were attracted to regions of high electric field intensity near the edges of circular microelectrodes, while larger bioparticles like cells were pulled into regions of low electric field intensity between electrodes. The separation process for a 50 µL plasma sample took 15 minutes, followed by washing the bulk plasma materials with a buffer, leaving exosomes concentrated around the circular microelectrodes. All operational procedures were completed within 30 minutes, and the platform supported subsequent on‐chip detection of exosomal proteins.

Dielectrophoresis techniques support high‐sensitivity and high‐resolution separation of various bioparticles due to the direct and tunable manipulation of particles induced by external electric fields.^[^ ^108^, ^117^ ^]^ Therefore, dielectrophoresis can not only achieve high‐purity isolation of different types of bioparticles, such as separating CTCs or bacteria from other sample components,^[^ ^26^, ^118^ ^]^ but also sort subpopulations of bioparticles including cells and exosomes.^[^ ^115^, ^119^ ^]^ However, dielectrophoresis separation techniques still face challenges caused by various factors and limitations, including the design of sophisticated system, microstructure fabrication, medium optimization, and the integration of electrodes with diverse sizes, shapes, and position layouts. The requirement for specific channel patterns and the introduction of electrical devices usually improve the overall cost and dimension of the system. Typically, dielectrophoresis devices usually require bioparticles to be suspended in isotonic buffers with low ionic strength,^[^ ^120^, ^121^ ^]^ which may prevent bioparticles from maintaining normal metabolic functions as in conventional physiological buffers, leading to a significant decline in their viability over time.^[^ ^122^, ^123^, ^124^ ^]^ Electrochemical reactions, such as electrolysis, are easily triggered at electrode edges, reducing the life time of device.^[^ ^125^, ^126^ ^]^ In addition, the high electrical conductivity of the medium often generates excessive joule heat,^[^ ^127^, ^128^ ^]^ which is harmful to bioparticles. Therefore, the maximum voltage applied to the electrodes is usually limited by aforementioned factors, which may result in the insufficient dielectrophoresis force to manipulate bioparticles under certain conditions,^[^ ^122^ ^]^ hindering the broader application of dielectrophoresis technologies in other research fields. Moreover, the high‐precision isolation of electrophoresis comes at the expense of reduced sample processing throughput, resulting in lower separation efficiency compared to other techniques.

#### 2.1.3 Acoustophoresis

**Figure 5:**
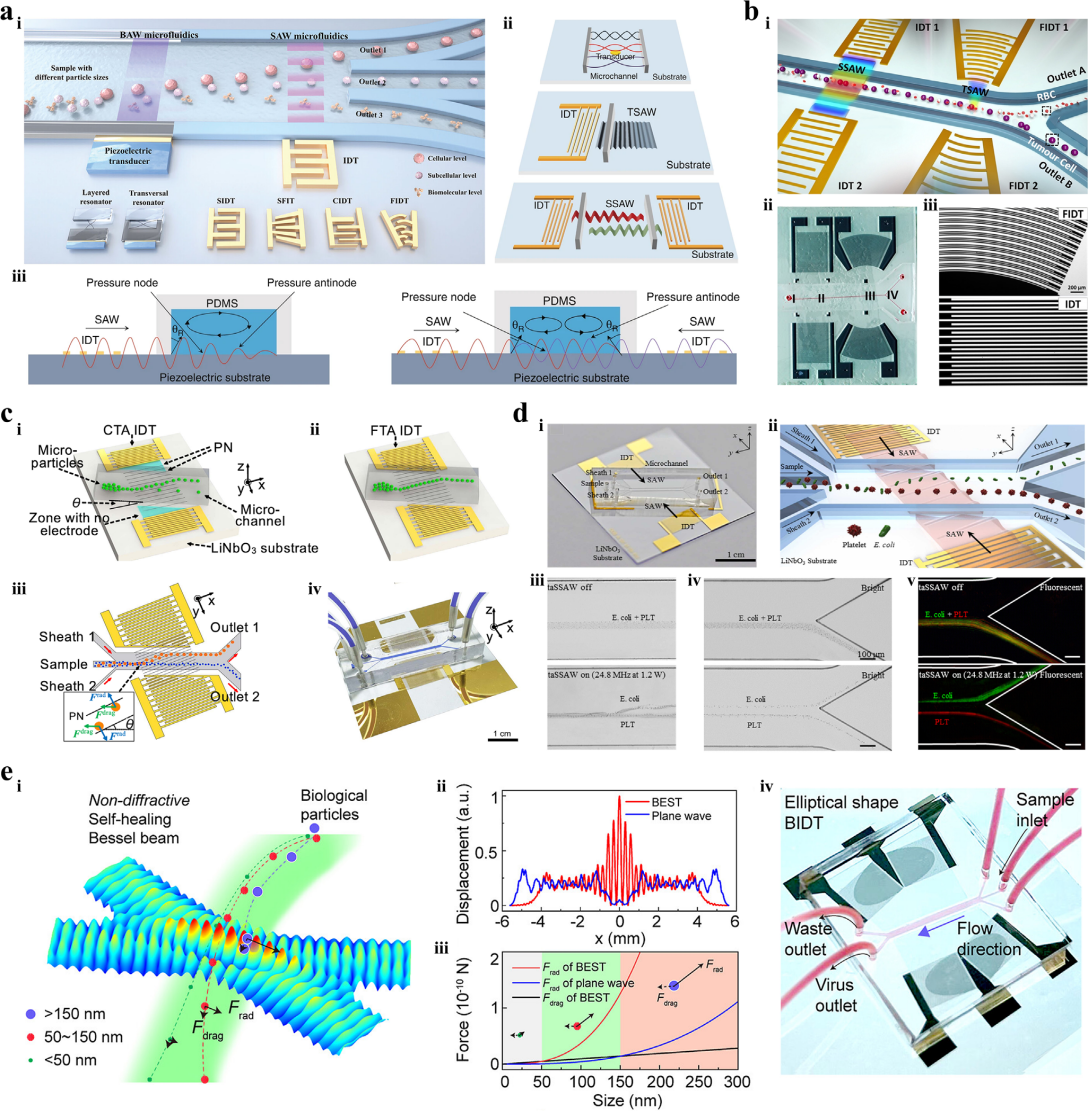
Acoustophoresis isolation techniques. a) Schematic of acoustophoresis separation.^[^ ^63^, ^136^ ^]^ Copyright 2023, Wiley. Copyright 2022, Springer Nature. i) Acoustophoresis systems are divided into two categories: BAW‐based and SAW‐based. ii) Schematic diagram of BAWs (top), TSAWs (middle), and SSAWs (bottom). iii) Acoustic streaming effect of TSAWs (left) and SSAWs (right). b) Multi‐stage acoustophoresis system based on SAWs for separating CTCs from blood cells.^[^ ^146^ ^]^ Copyright 2018, Elsevier. i) Schematic illustration of the platform. ii) Prototype device. iii) The microscopic image of finger pairs of IDT and FIDT. c) Filled tilted‐angle acoustofluidic chip for separating CTCs from peripheral blood mononuclear cells.^[^ ^142^ ^]^ Copyright 2021, Institute of Electrical and Electronics Engineers. i) Conventional tilted‐angle device. ii) Filled tilted‐angle device. iii) Schematic illustration of the system. iv) Prototype device. d) Tilted‐angle SSAW‐based platform for separating bacteria from platelets.^[^ ^17^ ^]^ Copyright 2024, Elsevier. i) Prototype device. ii) Schematic illustration of the device. iii) The trajectories of *E. coli* and platelets in the acoustic wave acting region under the condition of SSAW‐off (top) and SSAW‐on (bottom). iv)–v) The trajectories of *E. coli* and platelets under bright and fluorescent fields in the outlet region under the condition of SSAW‐off (top) and SSAW‐on (bottom). e) Bessel beam excitation separation technology for isolating viruses.^[^ ^12^ ^]^ Copyright 2024, American Chemical Society. i) Schematic illustration of acoustic standing Bessel beam formation. ii) Comparison of the acoustic intensity between Bessel beams and plane waves. iii) Comparison of Bessel beams induced acoustic radiation force, plane waves induced acoustic radiation force, and fluid drag force. iv) Prototype device.

Acoustophoresis separation techniques rely on acoustic radiation forces generated by acoustic waves, which are produced through the high‐frequency vibration of piezoelectric materials,^[^ ^129^, ^130^, ^131^ ^]^ such as piezoceramics and piezoelectric single crystals.^[^ ^6^, ^132^, ^133^ ^]^ According to the type of acoustic waves, which depends on whether just the piezoelectric material surface or the entire body vibrates, acoustophoresis systems are divided into two categories: bulk acoustic wave based (BAW‐based) and surface acoustic wave based (SAW‐based) (**Figure** **5**a).^[^ ^63^, ^134^, ^135^ ^]^ Acoustic waves are further categorized into unidirectional traveling wave with regular propagation and composite standing wave with bilateral transmission.^[^ ^136^, ^137^, ^138^ ^]^ BAWs are standing waves, and BAW‐based systems, including layered resonators and transversal resonators, are composed of a piezoelectric transducer, coupling layer, matching layer, fluid layer, and reflector layer.^[^ ^134^ ^]^ The piezoelectric transducer and coupling layer are used to generate acoustic waves and enable the ideal acoustic transmission into the system, respectively. The following matching layer forms the bottom of the resonator cavity and serves as a reflection surface for the standing wave in the fluid layer, which contains the fluid medium and bioparticles. The reflector layer, located at the other end of the system, is responsible for returning the incoming acoustic wave back into the fluid layer to form the standing wave. The main reflection of layered resonators producing the standing wave parallel to the actuation path occurs between the piezoelectric transducer and air backed reflector, while that of transversal resonators forming the standing wave perpendicular to the actuation path occurs between the channel walls.^[^ ^134^ ^]^ SAWs are elastic waves traveling along the piezoelectric material surface, and SAW‐based platforms involve interdigital transducer (IDT), piezoelectric substrate, and microchannel.^[^ ^139^, ^140^ ^]^ IDT has been optimized into four configurations including straight IDT (SIDT), slanted‐finger IDT (SFIT), chirped IDT (CIDT), and focused IDT (FIDT) for the requirement of flexibility and versatility.^[^ ^63^ ^]^ SAWs can be categorized into traveling surface acoustic waves (TSAWs) and standing surface acoustic waves (SSAWs).^[^ ^141^ ^]^ TSAWs are formed by an IDT on one side of the channel and SSAWs are generated by two opposing IDTs on both sides of the channel.^[^ ^142^, ^143^, ^144^ ^]^ TSAWs and SSAWs generate the acoustic radiation force acting on bioparticles, which enables the separation of bioparticles based on their size, density, and compressibility.^[^ ^17^, ^72^, ^144^, ^145^ ^]^

Wang et al.^[^ ^146^ ^]^ proposed a multi‐stage microsystem based on SAWs for separating CTCs from blood cells (Figure 5b). The platform consists of SIDTs and FIDTs which are used to generate SSAWs placing cells at the pressure nodes and form the focused TSAWs to separate CTCs from RBCs based on different sizes. The acoustic radiation force generated by unidirectional TSAW provides greater alignment tolerance for the system, improving the flexibility and stability of cell separation. Additionally, the pulsed TSAWs significantly weaken the accumulation of joule heat, which is beneficial for the viability and proliferation of cells. Wu at al.^[^ ^142^ ^]^ introduced a filled tilted‐angle acoustofluidic chip for separating CTCs from peripheral blood mononuclear cells (Figure 5c). Additional patterns of IDTs were introduced to adequately fill the adjacent device space of the microfluidic chip, reducing the footprint of the system and increasing acoustic energy density for improved cell manipulation. This system separated HeLa cancer cells from peripheral blood mononuclear cells with a high recovery rate (≈80%) through low input power (≈57% of conventional devices), effectively reducing heat generation and minimizing adverse effects on both the bioparticles and the device. Lee et al.^[^ ^17^ ^]^ investigated an acoustofluidic system for isolating *E. coli* from platelets (Figure 5d). Platelets are the smallest blood cells, with a size similar to that of *E. coli*, making it challenging to separate these two bioparticles using size‐based isolation techniques. However, under acoustic radiation force, microparticles with lower compressibility migrate further laterally, perpendicular to the flow direction, than those with higher compressibility. Based on this principle, tilted‐angle SSAWs induced greater acoustic force on *E. coli*, which has lower compressibility, compared to the more compressible platelets, enabling the high‐purity (>96%) and high‐recovery (>81%) separation of *E. coli* and platelets. Xia et al.^[^ ^12^ ^]^ proposed a Bessel beam excitation separation technique for isolating viruses from biological samples such as human saliva and cell culture media (Figure 5e). The Bessel IDT, utilizing an elliptical apodization pattern to form the enhanced acoustic resonant structure, improved the acoustic intensities and radiation force to levels five times higher than those generated by conventional plane‐wave IDTs. Therefore, this device generates sufficient acoustic radiation force to overcome the drag force, enabling the separation of target viruses or nanoparticles from interfering particles of different sizes. In addition, the cutoff diameter of microparticles can be flexibly modified for isolating various types of targets with different sizes by adjusting the input acoustic power. The tunable acoustic platform isolated SARS‐CoV‐2 and Moloney Murine Leukemia Virus from biological samples with a purity of 90.66% and a recovery rate of 86.5%.

**Figure 6:**
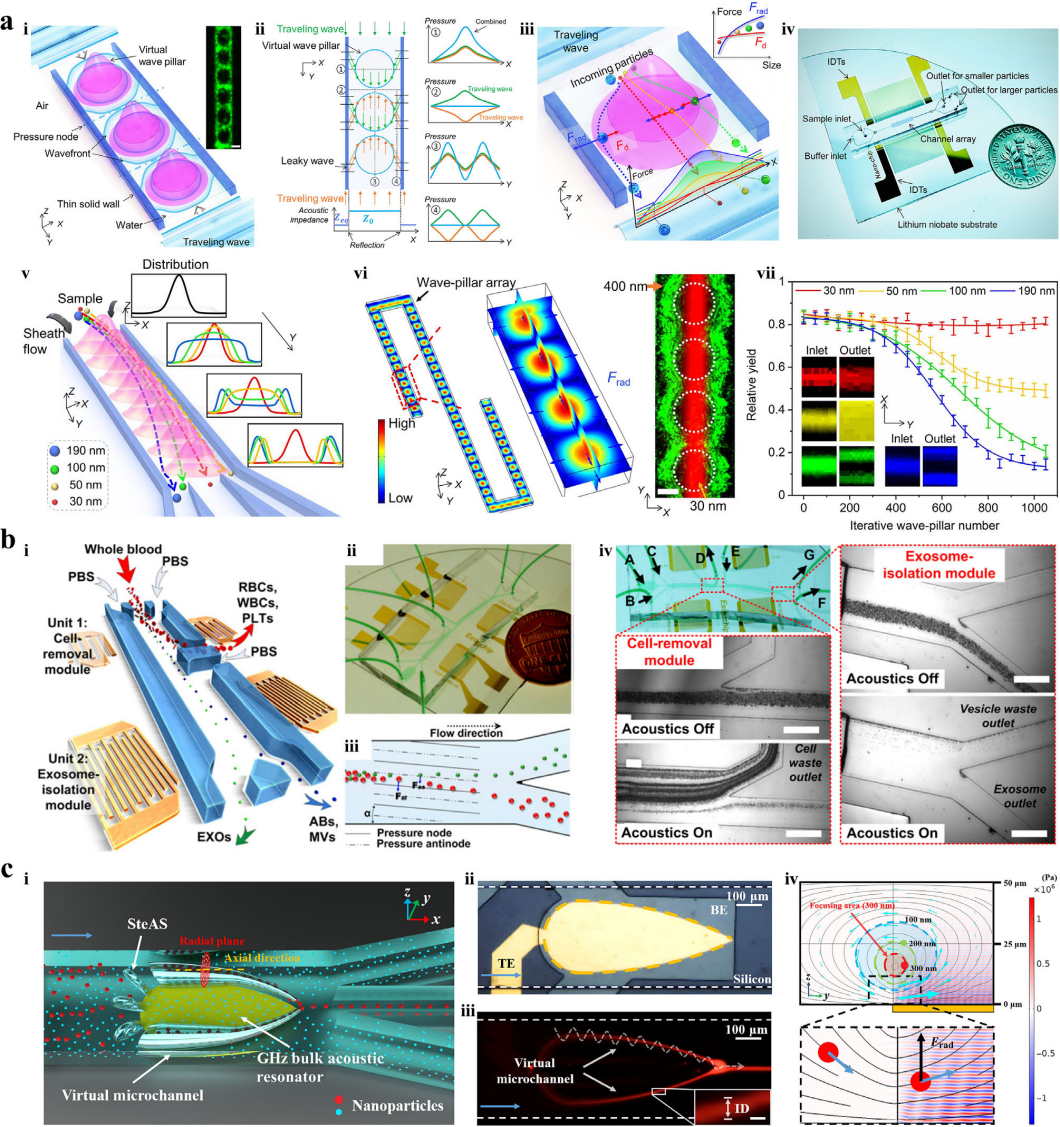
Acoustophoresis techniques for isolating exosomes. a) Acoustic nanoscale separation via wave‐pillar excitation resonance.^[^ ^147^ ^]^ Copyright 2022, American Association for the Advancement of Science. i) Schematic illustrating the formation of acoustic virtual wave pillars through IDTs. Inset, 2 µm fluorescent polystyrene particles exhibit the generation of virtual wave pillars. Scale bar: 20 µm. ii) Wave propagation analysis during the formation of virtual wave pillars. iii) Schematic illustration of the nanoparticle separation and the force analysis. Inset, force analysis for different‐sized nanoparticles. iv) Prototype device. v) The iterative process of the accumulation and the increase of the minimum lateral deterministic displacement for nanoparticle separation. vi) Simulation results of the acoustic pressure distribution in one channel unit and the experimental validation of the virtual wave pillars by the fluorescent pattern of 400 nm/30 nm polystyrene particles. Scale bar: 10 µm. vii) Relative yield for 30, 50, 100, and 190 nm nanoparticles by the number of wave pillars. b) Integrated acoustofluidic platform for isolating exosomes directly from undiluted human whole blood samples.^[^ ^3^ ^]^ Copyright 2017, Charlesworth. i) Schematic illustration of the system. ii) Prototype device. iii) The separating process of bioparticles near the outlets. iv) The experimental validation of separating exosomes from blood sample. Scale bar: 500 µm. c) Stereo acoustic stream platform for isolating exosomes from plasma directly.^[^ ^148^ ^]^ Copyright 2022, American Association for the Advancement of Science. i) Schematic of the stereo acoustic stream platform. ii) Actual gigahertz BAW system integrated with a microchannel. iii) Fluorescence image of nanoparticles (300 nm diameter) trapped in virtual channel and released at focal point. iv) Force analysis of the focusing process.

Zhang et al.^[^ ^147^ ^]^ developed a wave‐pillar excitation resonance technology for single‐step, high‐purity, and rapid isolation of small extracellular vesicles subpopulations, including exomeres (<50 nm), small exosomes (60–80 nm) and large exosomes (90–150 nm), from biological samples without the requirement of complex nanoscale fabrication and redundant sample processing procedures (**Figure** **6**a). The array of virtual acoustic wave pillars was formed by superposing the excitation resonance field and the original standing SAW field under the conditions of sufficient reflection of leaky waves and suitable frequency of input power. Two limitations of current acoustic‐based separation techniques: the rapid attenuation of acoustic force and the acoustic streaming induced by high‐frequency SAW, were broken by the iterative cumulative effect of multiple virtual posts. Therefore, small extracellular vesicles subpopulations of different sizes exhibited distinct positional deflections after passing through a virtual acoustic wave pillar, and the accumulative differences caused by the comprehensive effect of multiple virtual pillars drove various subpopulations to corresponding outlets. Additionally, the cutoff diameter can be precisely adjusted by controlling the acoustic power input, enabling the isolation of different small extracellular vesicle subpopulations without requiring the design of numerous nanoscale structures, as is necessary for mechanical filtration and DLD. In order to isolate exosomes from undiluted human whole blood sample, Wu et al.^[^ ^3^ ^]^ proposed an integrated acoustofluidic device consists of two sequential SAW modules: cell‐removal module and exosome‐isolation module, which both rely on a tilted‐angle SSAW generated by one pair of IDTs (Figure 6b). The cell‐removal module was used to extract the microscale blood cells to obtain concentrated extracellular vesicles, which were then further purified by exosome‐isolation module to remove other subgroups of extracellular vesicles. This size‐based acoustic separation system is capable of isolating exosomes from undiluted human whole blood with high purity (98.4%) and high recovery rate (82.4%), and the cutoff sizes can be adjusted for sorting different types of extracellular vesicle subgroups by tuning the input power. Yang et al.^[^ ^148^ ^]^ designed a stereo acoustic stream platform for manipulating nanoscale particles (Figure 6c). A series of closed and connected microscale vortices were triggered by ultrahigh‐frequency BAW and confined by the microchannel, generating an arc‐shaped virtual channel. Under continuous flow conditions, microparticles migrated downstream with a *y*‐directed motion while being limited within the virtual channel until they were released from the focal point. The focusing effect is significantly related to the particle size, thereby driving large particles to move in the stereo acoustic stream and flow into the center outlet, while the motion trajectories of small particles are almost unaffected. As a result, small target nanoparticles, such as exosomes, were separated from samples by removing the larger impurities.

Acoustophoresis is a kind of ultrahigh‐precision technique for separating bioparticles, particularly at the nanoscale. Acoustophoresis techniques have been widely utilized to isolate viruses and small extracellular vesicles subpopulations from complex samples, where target bioparticles are similar to other interfering components in size (diameter difference is ≈100 nm).^[^ ^149^, ^150^, ^151^ ^]^ In addition, the cutoff size of acoustophoresis technique can be precisely adjusted by controlling the acoustic power input, enabling the separation of various target bioparticles across different research contexts. This approach eliminates the need for repetitive and complex micro/nano‐scaled fabrication required by isolation methods such as mechanical filtration and DLD,^[^ ^69^, ^152^, ^153^ ^]^ thereby avoiding redundant time investments and manufacturing costs. Despite the aforementioned advantages, acoustophoresis still have many limitations. According to the separation mechanism of acoustophoresis, the acoustic radiation force weakens with the reduction of bioparticle size,^[^ ^154^ ^]^ necessitating higher acoustic wave intensity and operating frequency to manipulate nanoscale bioparticles.^[^ ^155^, ^156^ ^]^ However, the intense acoustic streaming is also formed as a byproduct which disrupts the laminar flow and reduces the isolation efficiency.^[^ ^63^ ^]^ As a result, it is challenging for current acoustophoresis techniques to strike a balance between the strength of the acoustic radiation force and negative effects. Additionally, practical application of acoustophoresis is constrained by low throughput, especially in nanoscale separation, and the need for sophisticated electromechanical systems, which include numerous instruments such as power amplifiers, function generators, and controllers.^[^ ^157^, ^158^, ^159^ ^]^ To address these limitations, in‐depth research into the mechanism of acoustophoresis is essential, along with the development of advanced fabrication technologies and the establishment of comprehensive design and manufacturing standards. On this basis, integrated design for acoustophoresis platforms and peripheral devices is further conducted to improve critical performance parameters such as throughput and size, thereby promoting the clinical application of acoustophoresis.

#### 2.1.4 Optophoresis

Optophoresis is a technology for precise manipulation of micro/nano‐scaled particles based on the optical force induced by momentum exchange between irradiated microparticles and photons.^[^ ^160^, ^161^, ^162^ ^]^ In general, optical forces are categorized into scattering force and gradient force, both of which can be calculated or summarized by Maxwell stress tensor.^[^ ^163^ ^]^ The scattering force pushes microparticles along the direction of light propagation and the gradient force drives microparticles to migrate toward the region with highest light intensity.^[^ ^131^, ^164^ ^]^ The gradient force is typically used to trap target particles (called optical tweezer),^[^ ^165^, ^166^ ^]^ but needs to be ignored in most particle sorting applications.^[^ ^161^ ^]^ Therefore, the collimated laser beam with a width larger than the particle size is usually utilized to generate the scattering force while minimizing the gradient force, enabling the separation of various particles with different physical properties,^[^ ^167^, ^168^ ^]^ such as sizes and refractive index.^[^ ^169^, ^170^ ^]^

**Figure 7:**
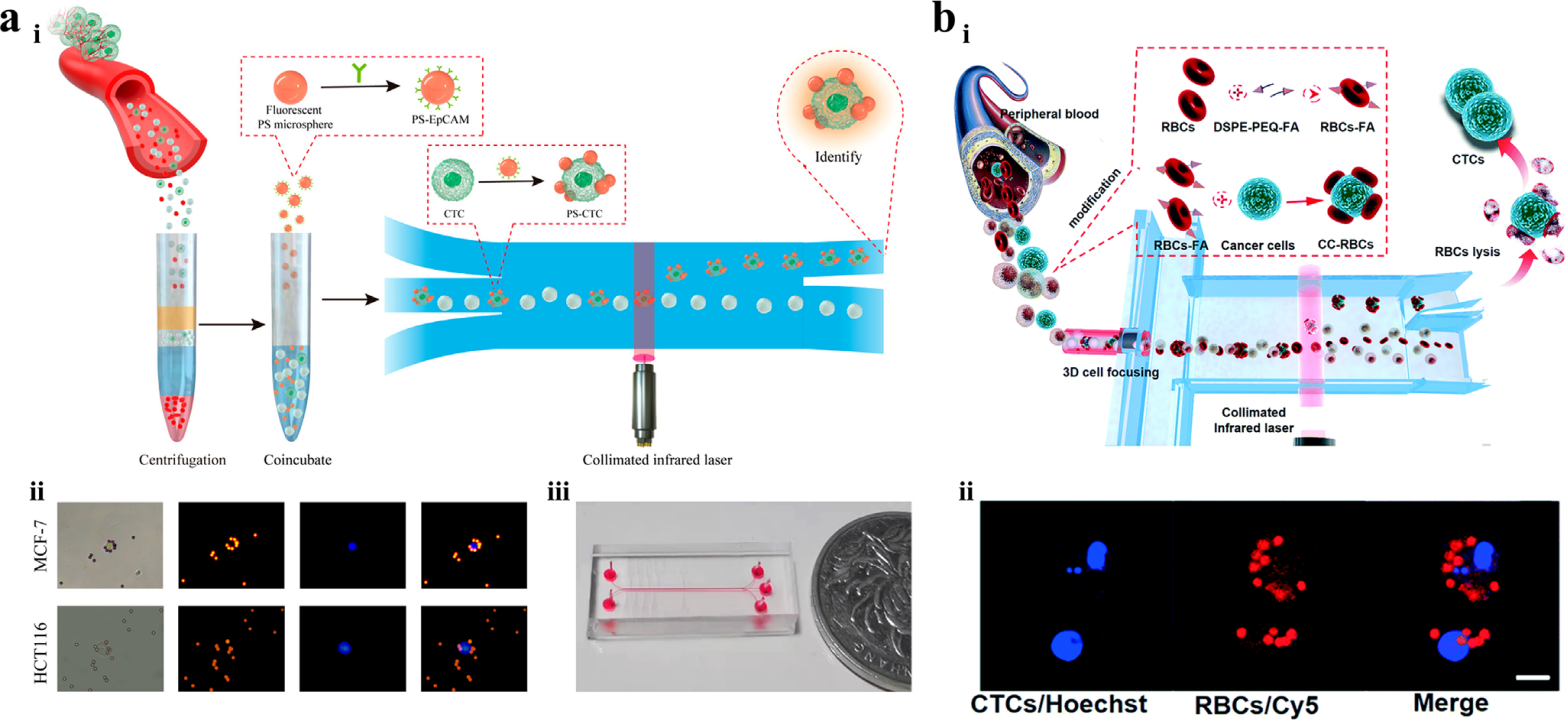
Optophoresis techniques. (a) Optical isolation of CTCs engineered by fluorescent microspheres.^[^ ^35^ ^]^ Copyright 2022, American Chemical Society. i) Schematic depiction of the device. ii) Bright field and fluorescence images of microsphere‐labeled CTCs. The nuclei of CTCs stained with DAPI and the fluorescent PS microspheres are shown in blue and orange, respectively. iii) Prototype device. (b) Optical isolation of CTCs engineered by RBCs.^[^ ^173^ ^]^ Copyright 2019, Royal Society of Chemistry. i) Schematic illustration of the platform. ii) The fluorescence images of RBC‐labeled CTCs. The nuclei of CTCs are stained with Hoechst (blue) and the membranes of RBCs are labeled with Cy5 (red).

Chen et al.^[^ ^35^ ^]^ proposed an optical microfluidic platform for separating fluorescent microsphere modified CTCs (**Figure** **7**a). Due to the similar sizes and refractive indices of WBCs and CTCs,^[^ ^171^, ^172^ ^]^ target CTCs cannot be directly separated from the sample. Therefore, fluorescent polystyrene microspheres with a high refractive index were modified with EpCAM antibody to capture CTCs, significantly improving the average refractive index of the modified CTCs. Furthermore, the fluorescence signals of polystyrene microspheres were utilized to directly and rapidly identify collected cells, avoiding the multiple cumbersome steps of cellular immunostaining. Hu et al.^[^ ^173^ ^]^ utilized folic acid as the target agent to bind CTCs and RBCs (Figure 7b), increasing the size and mean refractive index of the RBC‐labeled CTCs due to the larger refractive index of RBCs.^[^ ^174^ ^]^ This technique adopted homologous RBCs to label CTCs instead of introducing foreign materials such as magnetic microbead, exhibiting multiple advantages of good biocompatibility and easy access. Experimental results indicated that CTCs modified with RBCs were separated from blood sample with high purity (>92%) and high recovery rate (>90%).

Optophoresis relies on the optical force which is significantly dependent on various physical properties of bioparticles, not just the geometric parameters. Therefore, similar size bioparticles such as WBCs and CTCs can be precisely isolated by adjusting the refractive index through labeling techniques. In addition, optophoresis techniques can maintain the original state of bioparticles, reducing the probability of bioparticle damage.^[^ ^35^ ^]^ However, optophoresis often relies on complex precision instruments for assistance, which are typically high‐cost and bulky. In addition, labeling techniques are usually time‐consuming and require limiting the sample flow rate during subsequent separation processes to achieve ideal purity and recovery rate. In general, integrating optophoresis technologies into microsystems for the rapid and reliable isolation of target bioparticles is challenging, posing a significant hurdle for their widespread adoption in clinical applications.

#### 2.1.5 FAS

Typical FAS systems include three major modules:^[^ ^61^ ^]^ focusing module, detection module, and deflection module. Single bioparticle stream can be generated in the focusing module by external devices such as IDT or utilizing continuous oil phase on both sides to break the dispersed water phase into discrete water‐in‐oil droplets.^[^ ^61^, ^175^ ^]^ The aligned bioparticles are identified individually in the detection module based on the fluorescence signal or fluorescence image.^[^ ^176^, ^177^ ^]^ If the target bioparticle is detected, the deflection module generates the triggering signals that drive controller to form the physical field, propelling the target bioparticle toward the corresponding outlet by the external acoustic or electric forces.^[^ ^178^, ^179^ ^]^

**Figure 8:**
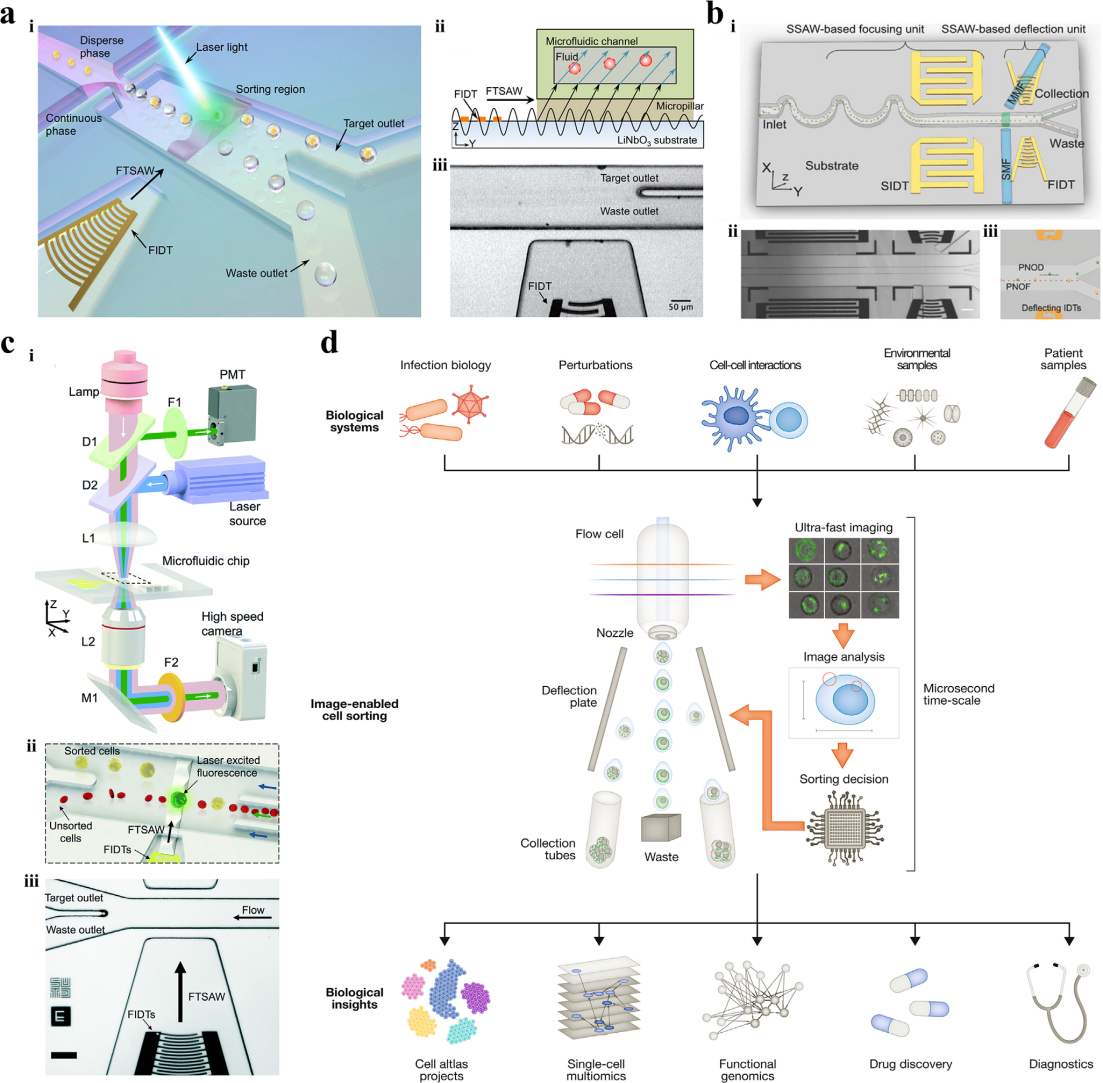
FAS techniques. a) FADS system for isolating CTCs at single‐droplet level.^[^ ^175^ ^]^ Copyright 2019, American Chemical Society. i) Schematic depiction of the platform. ii) The cross‐sectional view of the FADS system. iii) The microscopic image of the sorting region in the FADS system. b) SSAW‐based FACS chip.^[^ ^61^ ^]^ Copyright 2018, Wiley. i) Schematic illustration of the platform. ii) Microscopic image of the focusing module and detection module. Scale bar: 200 µm. iii) The position of pressure nodes of the deflecting IDT and the focusing IDT. c) FACS system for separating CTCs from human whole blood.^[^ ^180^ ^]^ Copyright 2017, Royal Society of Chemistry. i) Schematic depiction of the optical device for the fluorescence interrogation and the high‐speed imaging. ii) The process of separating CTCs from blood sample. iii) The microscopic image of the sorting area. Scale bar: 100 µm. d) Combining imaging with FACS technique enables novel experiment strategies.^[^ ^176^ ^]^ Copyright 2023, Wiley.

Li et al.^[^ ^175^ ^]^ proposed a fluorescence‐activated droplet sorting (FADS) system for isolating droplets encapsulating single CTC (**Figure** **8**a). Droplets were periodically generated in the flow‐focusing microfluidic platform, where the continuous oil phase flowing from two sides broke the dispersed water phase into discrete water‐in‐oil droplets. Droplets without target CTCs flowed into the waste outlet due to the low flow resistance, while droplets containing target CTCs labeled with fluorescence were identified as they reached the detection region. The fluorescent signals were excited and transmitted to photomultiplier tube which converted the fluorescence intensity into an electrical signal used to trigger a pulse alternating signal. As a result, TSAW beam was formed by the FIDT, generating the acoustic radiation force that propelled droplets containing target CTCs toward the designated outlet. Ren et al.^[^ ^61^ ^]^ developed a fluorescence‐activated cell sorting (FACS) system to isolate fluorescent labeled HeLa cells from unlabeled HeLa cells based on SSAW, without the utilization of sheath fluid and the precise control of flow rate (Figure 8b). The serpentine channel was introduced to preconcentrate cells near the designated pressure node, preventing cells from aggregating together and generating the uniform space between adjacent cells. All cells were further focused into the closest single pressure node in the SSAW‐based focusing module through SIDT, forming a single‐cell stream. The fluorescent labeled HeLa cells were then identified in the detection module and separated by FIDT in the deflection module. This separation occurred because the pressure node of the deflecting SSAW, compared to that of the focusing SSAW, was shifted and aligned with the collection outlet. In order to further verify the practical application value of FAS technology, Ma et al.^[^ ^180^ ^]^ investigated a FACS platform to sort CTCs from human whole blood sample, with high purity (>86%) and high viability (>95%) (Figure 8c). The working principle is similar with the aforementioned methods, CTCs stained with SYTO 9 fluorescent dye were confined by two sheath flows to form the single‐cell stream, ensuring that the non‐fluorescent interfering components reliably followed the original sample into the waste outlet. The fluorescent labeled CTCs were detected and isolated in interrogation region and deflection region, respectively. Traditional FACS techniques rely solely on simple morphological parameters, such as approximate cell size or protein expression levels, for cell separation. However, enriching cells based on spatial phenotypes of interest can provide deeper insights into the causes and consequences of cellular morphology. Therefore, Schraivogel et al.^[^ ^176^ ^]^ proposed combining imaging with FACS (Figure 8d), which allows for the recording of spatial phenotypic changes, such as alterations in the intracellular localization of proteins. This novel combined FACS technique is advantageous for identifying new cell types based on spatial phenotypes and separating them for functional characterization.

FAS techniques can separate fluorescent labeled cells from complex sample with ultrahigh precision owing to individual detection. However, the entire system, consisting of a focusing module, detection module, and deflection module, is typically bulky and high‐cost, posing a challenge in seamlessly integrating different modules with complex designs into a compact and all‐in‐one platform. The processes of fluorescent labeling of cells, forming an aligned single‐cell stream, and identifying the fluorescence signals typically take some time for preprocessing and limit the flow rate of cells during separation, severely reducing the processing throughput. In addition, FAS technology is mainly utilized to the cell separation and face challenges in isolating submicron bioparticles, such as exosomes and viruses, limiting the expansion of FAS technique to other research fields and clinical applications.

### 2.2 Passive Methods

Passive methods rely on intrinsic hydrodynamic forces generated by the coupling effect between microstructures, fluid, and bioparticles to separate target bioparticles from samples.^[^ ^41^, ^181^ ^]^ Passive separation techniques can be classified into five categories based on the shapes of microstructures and the types of forces acting on bioparticles: IMF, VEM, DLD, microfiltration and PFF.^[^ ^182^, ^183^, ^184^ ^]^

#### 2.2.1 IMF

**Figure 9:**
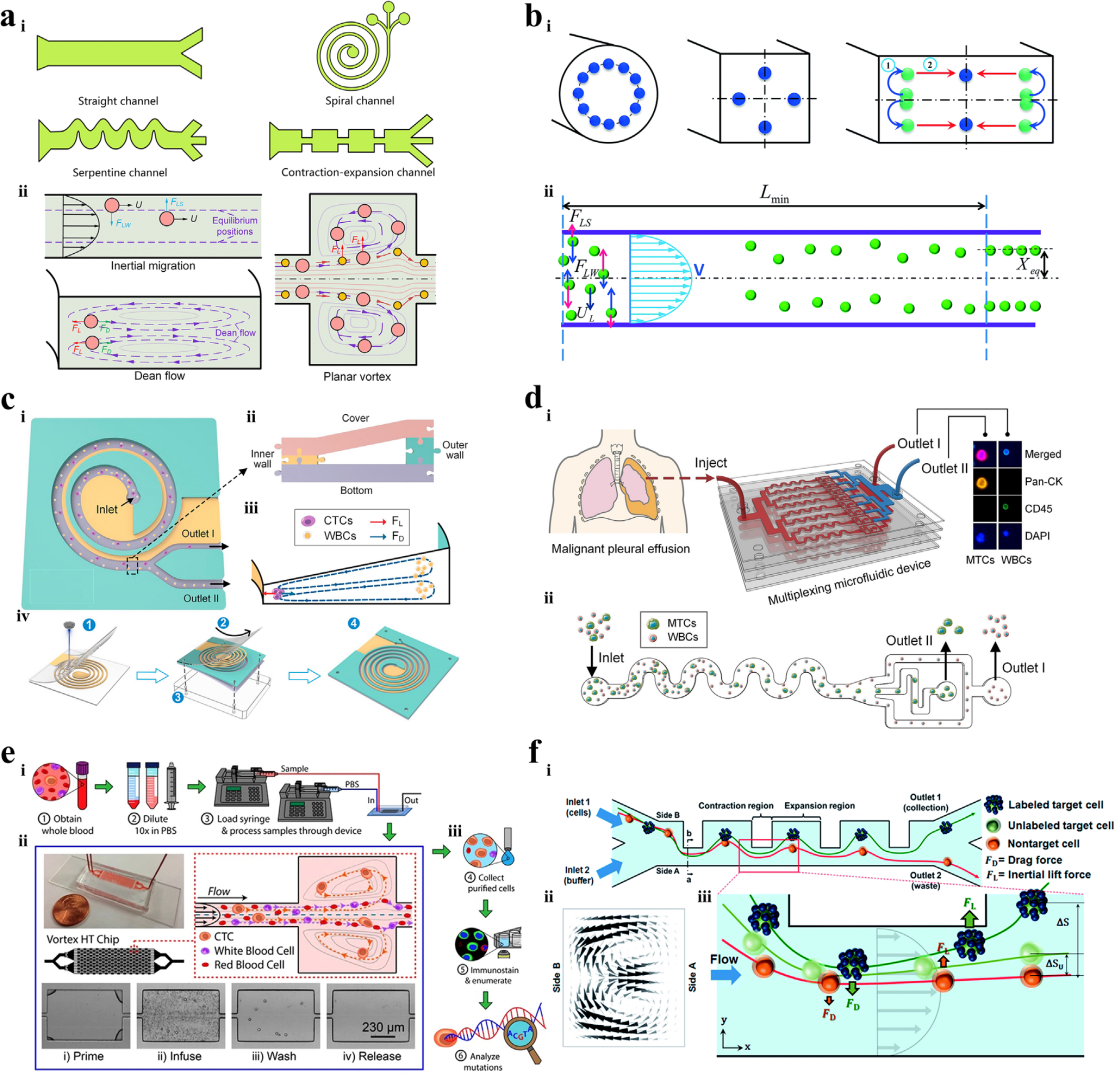
IMF techniques. a) Geometric and physical mechanisms of IMF.^[^ ^44^ ^]^ Copyright 2022, Royal Society of Chemistry. i) Common channel patterns. ii) Separation mechanism. b) Straight channels in IMF.^[^ ^41^ ^]^ Copyright 2016, Royal Society of Chemistry. i) Cross‐section shapes. ii) The equilibrium process of particles. c) Polymer‐film IMF sorter.^[^ ^204^ ^]^ Copyright 2021, Elsevier. i) Schematic depiction of the chip. ii) The trapezoidal cross section. iii) Distributions of CTCs and WBCs in trapezoidal spiral channels. iv) Fabrication process. d) The multiplexed IMF with ultra‐high separation throughput.^[^ ^205^ ^]^ Copyright 2021, Elsevier. i) The geometry of the multiplexed IMF. ii) The separation process of malignant tumor cells and WBCs in the serpentine channel. e) Vortex microfluidic technology.^[^ ^216^ ^]^ Copyright 2017, Springer Nature. i) The preprocessing before separating. ii) The isolating process. iii) The postprocessing after sorting. f) IMF with asymmetric contraction‐expansion microchannels.^[^ ^218^ ^]^ Copyright 2014, Royal Society of Chemistry. i) Schematic illustration of the device. ii) The Dean flow at the entrance of contract channels. iii) Motion trajectories of different types of cells in contract and expanded channels.

Inertial migration refers to the phenomenon where randomly distributed particles gradually migrate along the lateral direction in Newtonian fluid and ultimately reach equilibrium at multiple positions in the cross‐section of straight, curved, and contraction‐expansion channels (**Figure** **9**a).^[^ ^44^, ^185^, ^186^ ^]^ Particles flowing in the straight microchannel are subjected to multiple forces such as shear gradient induced lift force,^[^ ^186^, ^187^ ^]^ wall‐induced lift force,^[^ ^64^, ^188^ ^]^ viscous drag force,^[^ ^41^ ^]^ Saffman lift force,^[^ ^189^ ^]^ and Magnus lift force.^[^ ^190^ ^]^ In most cases, the Saffman lift force and Magnus lift force are much smaller than other forces,^[^ ^191^ ^]^ and the lateral component of viscous drag force is also negligible due to the minimal relative velocity difference between particles and the fluid. Therefore, the lateral migration of particles in straight channel is primarily caused by the competition effect of shear gradient induced lift force and wall‐induced lift force,^[^ ^41^, ^192^ ^]^ and their resultant force is referred to as the inertial lift force.^[^ ^193^ ^]^ All types of particles eventually migrate to the same equilibrium positions, which are independent of the particle geometric characteristics and determined solely by the Reynolds number and the cross‐sectional shape of the microchannel (Figure 9b).^[^ ^41^, ^49^ ^]^ Although various particles eventually balance at several designated positions in the channel cross‐section, particle separation can be achieved by exploiting the different migration velocities of particles with distinct dimensions.^[^ ^194^, ^195^ ^]^

Secondary flow arises in the cross‐section when fluid flows through a curved channel, due to the mismatch in flow velocities between the center and near‐wall regions.^[^ ^196^, ^197^ ^]^ Fluid elements near the centerline of the channel, having larger inertia, tend to flow toward the outside of the curved channel, generating a radial pressure gradient that causes the fluid near the walls to re‐circulate into two symmetric vortices, known as Dean flow.^[^ ^194^ ^]^ Under the combined effect of inertial lift force and Dean drag force, particles of different sizes reach new equilibrium positions on either side of the curved channel and flow into corresponding outlets, thereby achieving separation.^[^ ^198^, ^199^, ^200^ ^]^ Curved channels are mainly divided into spiral channel and serpentine channel,^[^ ^201^, ^202^, ^203^ ^]^ Zhu et al.^[^ ^204^ ^]^ proposed a polymer‐film IMF with trapezoidal spiral channels through precisely assembling polymer‐film jigsaws (Figure 9c). This approach bypassed complex and costly fabrication processes such as micro‐milling and PDMS soft lithography, while also improving the accuracy of the channel shape. This microfluidic chip was fabricated based on the laser cutting system and bonded through plasma, the inner and outer walls were cut from the silicon rubber sheet plates, while the bottom and cover jigsaws were cut from polyvinyl chloride support plates. This trapezoidal spiral channel offers better separation resolution compared to traditional rectangular spiral channels and successfully isolates CTCs from blood sample with a purity of 79.05% and a recovery rate of 94.14%. The size of IMFs with spiral channels is generally larger than that of other microfluidic chips, which presents challenges in parallelization design to further improve sample processing throughput. To this end, Ren et al.^[^ ^205^ ^]^ developed a polymer‐film multiplexed IMF with parallel serpentine channels to enable the ultra‐fast isolation of cancer cells from malignant pleural effusion (Figure 9d). In each serpentine channel, malignant tumor cells with larger size gradually focused at the channel centerline, while WBCs were primarily distributed near the channel sidewalls. Although the throughput of multiplexed IMF is significantly improved by the parallelization design, there is a notable decrease in both purity and recovery rate. Experimental results show that the purity and recovery rates are 54% and 81% under the throughput of 9.6 mL·h^−1^, whereas those are just 29% and 65% under the throughput of 28.8 mL·h^−1^.

Contraction‐expansion channel consists of periodic contracted and expanded channels,^[^ ^206^, ^207^, ^208^ ^]^ and can be divided into two categories: symmetric and asymmetric.^[^ ^209^, ^210^, ^211^ ^]^ For symmetric channels, two symmetric vortices are generated within an expanded channel on both sides when the narrow flow beam enters from the contracted channels into expanded channels. The wall‐induced lift force is significantly weakened due to the sudden increase in the distance between particles and side walls.^[^ ^212^, ^213^ ^]^ Therefore, under the comprehensive effect of shear gradient induced lift force and viscous drag force, larger particles are drawn into vortices due to the faster lateral motion speed and gradually migrate toward the center of vortices.^[^ ^214^, ^215^ ^]^ However, smaller particles with lower lateral migration velocity are unable to enter vortices and continue to flow downstream with the fluid. These motion patterns are continuously repeated in periodic contraction‐expansion channels, and larger bioparticles are eventually captured in vortices within various expanded channels to separate with smaller particles flowing into waste outlets. Renier^[^ ^216^ ^]^ developed a vortex microfluidic chip with symmetric expanded channels to isolate prostate CTCs from human whole blood, achieving a purity of 37.59% and a throughput of 8 mL·min^−1^ (Figure 9e). The vortex microfluidic technology was verified to successfully identify 80% of prostate cancer patients, based on the threshold of 3.37 CTCs/mL, which was calculated from five age‐matched healthy donors. The separating process involves priming the channels with a wash solution to remove bubbles, followed by infusing the blood sample and capturing prostate CTCs in the expanded channels. The wash solution, flowing at the same rate as the sample, is then used to remove residual blood cells. Finally, captured prostate CTCs are released by dissipating the vortices, which is achieved by reducing the flow rate. There is only one side of expanded channels in the asymmetric contraction‐expansion structures, and the Dean flow is formed when the fluid enters the contract channels from expanded channels due to the centrifugal effect.^[^ ^217^ ^]^ All particles are initially focus near the wall of expanded channel, and smaller particles, which are dominated by the Dean flow, migrate further toward the straight wall in the contract channels.^[^ ^218^ ^]^ However, larger particles, which are primarily influenced by the inertial lift force, migrate back toward the side wall of expanded channels at a higher velocity.^[^ ^209^ ^]^ Therefore, significant spatial position differences between particles of distinct sizes are generated after they pass through the contraction‐expansion channel array. Shin et al.^[^ ^218^ ^]^ proposed an asymmetric contraction‐expansion array microchannel for separating CTCs from samples (Figure 9f). In order to isolate cells with similar sizes, target CTCs were labeled with microbeads through the immune‐specifical reaction to increase the size difference between target CTCs and non‐target cells. The purity, recovery rate, and throughput of this IMF with asymmetric contraction‐expansion channels are 73.8%, 97.6%, and 0.875 mL·h^−1^, respectively.

IMF is currently one of the most widely used passive isolation techniques due to the advantages of high throughput (≈mL·min^−1^) and simple structure.^[^ ^219^, ^220^, ^221^ ^]^ However, IMF techniques rely on the Newtonian fluid medium,^[^ ^29^ ^]^ and the sorting efficiency for target bioparticles, such as CTCs, are susceptible to being disrupted by high concentrations of blood cells. Therefore, RBCs in human whole blood samples are usually lysed, and the remaining sample is diluted several tens of times before isolation,^[^ ^2^, ^222^ ^]^ which adds inconvenience to the process and reduces the actual processing throughput for original blood samples. In addition, the size of IMF devices is generally larger than that of other passive isolation microfluidic chips, as the separation phenomenon depends on a long flowing distance,^[^ ^204^ ^]^ which complicates further integration of IMF.

#### 2.2.2 VEM

**Figure 10:**
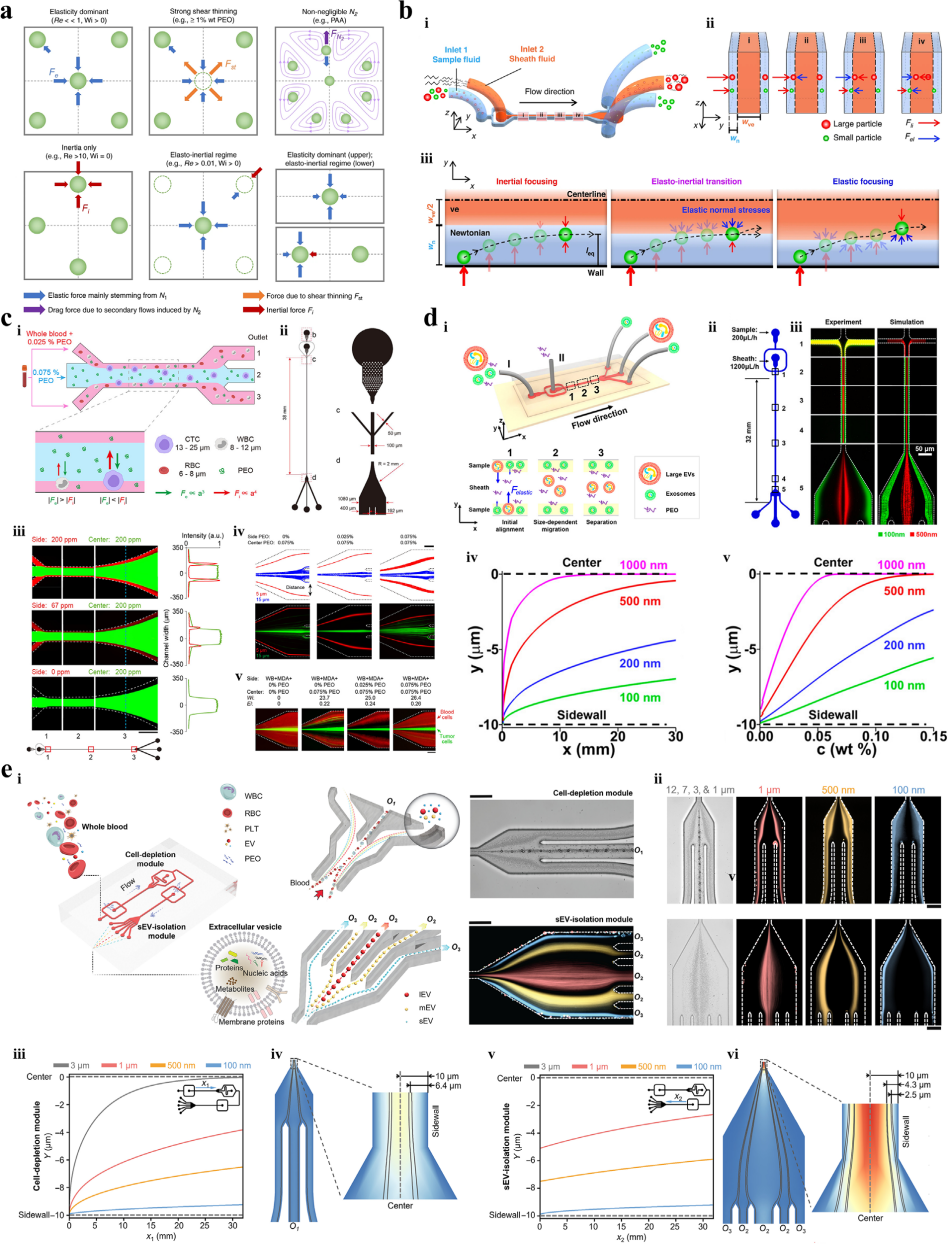
VEM techniques. a) Multiple forces govern the equilibrium position of particles in the channel cross‐section.^[^ ^40^ ^]^ Copyright 2020, Springer Nature. b) Schematic illustration of VEM used to isolate micron particles.^[^ ^73^ ^]^ Copyright 2024, Springer Nature. i) Size‐selective microparticle separation utilizing a co‐flow of viscoelastic and Newtonian fluids. ii) The separation process. iii) Migration trajectories of particles in the microchannel of VEM. c) PEO concentration gradient‐based VEM for isolating CTCs.^[^ ^22^ ^]^ Copyright 2023, American Chemical Society. i) Schematic illustration of the platform. ii) The system design diagram. iii) The visualization of PEO concentration gradient. Scale bar: 200 µm. iv) The simulation (top) and experimental (bottom) results of the trajectories for 5 µm and 15 µm particles under different concentration gradients of PEO. Scale bar: 200 µm. v) The trajectories of blood cells and MDA‐MB‐231 cells under different PEO concentration gradients. Scale bar: 200 µm. d) VEM device for isolating exosomes from extracellular vesicles.^[^ ^233^ ^]^ Copyright 2017, American Chemical Society. i) Schematic of the separation platform. ii) The system design diagram. iii) Experimental (left) and simulation (right) results of the trajectories for 100 nm and 500 nm particles. iv) The calculated trajectories of nanoparticles with different diameters in the straight channel. v) The calculated lateral positions of different nanoparticles at the end of straight channel under various PEO concentrations. (e) Cascaded VEM for isolating small extracellular vesicles from human whole blood.^[^ ^53^ ^]^ Copyright 2023, American Association for the Advancement of Science. i) Schematic illustration of the device. ii) Experimental results of different particle trajectories in the separation sections of both cell depletion module (top) and small extracellular vesicles isolation module (bottom). iii) Calculated particle trajectories in the straight channel of the cell depletion module. iv) The simulation result of flow field distribution at the separation section of the cell depletion module. v) Calculated particle trajectories in the straight channel of small extracellular vesicles isolation module. vi) The simulation result of flow field distribution at the separation section of small extracellular vesicles isolation module.

VEM systems work under the circumstances where viscoelastic fluids are introduced into microfluidic chips, with the viscoelastic force playing the major role in the particle separating process.^[^ ^223^, ^224^ ^]^ Viscoelastic fluids are prepared by dissolving synthetic polymer powders, such as polyethylene oxide (PEO),^[^ ^225^, ^226^ ^]^ polyvinylpyrrolidone (PVP),^[^ ^227^, ^228^ ^]^ and polyacrylamide (PAA),^[^ ^228^ ^]^ into DI water or PBS. The migration of particles in a viscoelastic fluid is comprehensively governed by multiple forces stemming from the fluid inertial, elasticity, shear thinning effect, and secondary flow induced by the second normal stress difference (**Figure** **10**a).^[^ ^23^, ^40^, ^229^ ^]^ The strength of the latter three effects depends on the properties and concentrations of polymer macromolecules.^[^ ^224^, ^230^, ^231^ ^]^ For example, PVP with a relatively small molecular weight can be safely regarded as Boger fluid, which has shear rate‐independent viscosity,^[^ ^232^ ^]^ but exhibits a relatively low elastic effect.^[^ ^233^ ^]^ Conversely, although PAA with the largest molecular weight can provide sufficient elastic force, the unconducive factors for separation, including the shear thinning effect and secondary flow, are also prominent.^[^ ^224^ ^]^ Therefore, most studies on VEM techniques adopt the intermediate molecular weight polymer PEO to prepare the viscoelastic fluids.^[^ ^234^, ^235^ ^]^ Under specific PEO concentrations, PEO solution can exhibit strong elasticity while ignoring the effects of shear thinning and secondary flow.^[^ ^233^, ^236^ ^]^ Based on the above analysis, particles flowing in the microchannels of VEM are primarily subjected to the inertial lift force and elastic lift force in the lateral direction.^[^ ^237^ ^]^ Viscoelastic sheath fluids and samples are typically introduced into VEM chip from multiple inlets simultaneously, reliably forming an interface between the sheath fluid and sample fluid due to the negligible diffusion of PEO molecules.^[^ ^238^, ^239^, ^240^ ^]^ Particles align near the sidewalls when just entering the channel in VEM, and the inertial lift force and elastic lift force are both center‐directed and push particles away from the walls (Figure 10b). The direction of elastic lift force reverses when particles reach the interface due to the higher stress on the side of sheath fluid. Since both inertial lift force and elastic lift force are size‐dependent, larger particles can fully penetrate the interface, continue moving toward the channel centerline, and finally reach their balance position. However, smaller particles can only partially penetrate the interface and balance near the interface due to the dominant effect of side‐directed elastic force.^[^ ^73^, ^239^, ^240^ ^]^ As a result, various particles will migrate to different lateral equilibrium positions through the barrier effect caused by the interface. Moreover, the barrier effect is insignificant for submicron particles, and thus the separation of nanoparticles is achieved by the different lateral migration velocities.^[^ ^233^ ^]^

Cheng et al.^[^ ^22^ ^]^ proposed a PEO concentration gradient‐based VEM for isolating CTCs from undiluted human whole blood sample (Figure 10c). In order to visualize the PEO concentration gradient, 100 nm polystyrene fluorescence particles with the similar sizes to the 600 kDa PEO molecules (gyration radius of 48 nm) were used to characterize the PEO concentration distribution.^[^ ^238^ ^]^ This approach is preferred over small‐size fluorescent dyes (a few nanometers), as the latter diffuses too quickly in the fluid. The competition between the center‐directed inertial lift force and sidewall‐directed elastic lift force resulted in distinct migration trajectories for cells of different diameters, thus achieving the separation of particles. Due to the introduction of the elastic lift force, the interface between the sheath fluid and the blood sample effectively shields the blood cells. As a result, this VEM technique enables the direct separation of target CTCs from untreated blood samples, eliminating the need for labor‐intensive and time‐consuming procedures. Liu et al.^[^ ^233^ ^]^ verified the feasibility of VEM technology for separating exosomes (Figure 10d). Different from the separation of micron‐sized bioparticles, the barrier effect of the interface is negligible for submicron bioparticles. Hence, the isolation of exosomes from complex media containing a variety of extracellular vesicles was realized by the different lateral migration velocities of bioparticles with distinct sizes. The large extracellular vesicles, with faster lateral velocities, moved to the vicinity of the channel centerline when arriving to the end of the straight channel. In contrast, the exosomes experienced only minimal lateral displacements, causing the exosomes and other bioparticles to flow into the center channel and two side channels, respectively. In order to further demonstrate the application value of VEM in isolating exosomes from human whole blood, Meng et al.^[^ ^53^ ^]^ developed a cascaded VEM including two sequential modules: the cell depletion module and the small extracellular vesicle isolation module (Figure 10e). Blood components larger than 1 µm in diameter, including WBCs, RBCs, and platelets, were first removed in the cell depletion module. The cell‐free sample then flowed downstream into the small extracellular vesicle isolation module, where small extracellular vesicles were separated from other extracellular vesicles. Due to the advantages of low cost, simple operation, and minimal sample preparation requirements, VEM is a more desirable technique for isolating exosomes compared to acoustophoresis, which may potentially damage the membranes of exosomes.^[^ ^241^ ^]^

The significant advantages of VEM isolation techniques lie in the ability to directly sort target particles from undiluted whole blood samples and the ultra‐high separating resolution (hundreds of nanometers).^[^ ^22^, ^242^ ^]^ VEM avoids labor‐intensive and time‐consuming sample preprocessing operations and can accurately isolate submicron target bioparticles, such as exosomes.^[^ ^243^, ^244^ ^]^ In addition, VEM chips typically use long straight channels as the main structure, which are significantly smaller than spiral IMF chips. This design facilitates parallelization for higher throughput and easier integration with other units, such as downstream detection modules. However, VEM techniques rely on the viscoelastic sheath fluids prepared through dissolving the polymer powders into DI water, and the above preparation process typically takes several tens of hours.^[^ ^22^ ^]^ Moreover, the separating dynamics in the microchannels of VEM are not fully understood, and the design of VEM structures is primarily based on numerous experimental results,^[^ ^40^ ^]^ restricting the flexible expansion of VEM to other research fields and engineering applications.

#### 2.2.3 DLD

**Figure 11:**
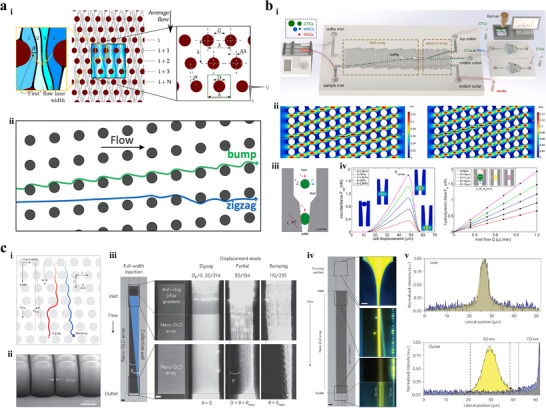
DLD techniques. a) Typical DLD microfluidic chip.^[^ ^245^, ^252^ ^]^ Copyright 2021, Wiley. Copyright 2020, American Chemical Society. i) The geometry of pillar array. ii) The bump mode and the zigzag mode of particles. b) Multistage DLD microfluidic device.^[^ ^47^ ^]^ Copyright 2023, Elsevier. i) Schematic illustration of the system. ii) Particles of the same size moved in zigzag and bump modes within circular and droplet‐shaped pillar arrays respectively, indicating that the droplet‐shaped pillar arrays have a smaller critical diameter. iii) Force analysis of cells in cone channel. iv) The counterforce and hydrodynamic force acting on cells within the cone channel. c) DLD microfluidic chip with nanoscale pillar array.^[^ ^69^ ^]^ Copyright 2016, Springer Nature. i) Schematic illustration of the device. ii) Scanning electron microscope image of nano pillar array. iii) Optical microscope image of nano DLD microfluidic chip. iv) Experimental results of isolating particles with diameters of 50 nm and 110 nm. v) The fluorescence intensity line at inlet and outlet.

The DLD microfluidic chip consists of a flat channel filled with a tilted array of pillar obstacles, which are used to generate fluid bifurcation and multiple streamlines between the pillar gaps.^[^ ^245^, ^246^, ^247^ ^]^ Each subsequent row of pillar array is offset by a certain distance, generating an inclination angle relative to the flow direction,^[^ ^248^, ^249^ ^]^ and creating a periodic flow pattern where the fluid is divided into distinct flow lanes by multiple stagnation streamlines as it passes through the gaps (**Figure** **11**a).^[^ ^250^, ^251^, ^252^ ^]^ Particles with a radius smaller than the width of the first flow lane will follow the original flow lane and move in a zigzag mode. In contrast, larger particles will migrate laterally to the next flow lane due to the collision with pillars and move along the array inclination angle.^[^ ^45^, ^253^ ^]^ The cutoff dimension between the bump mode and the zigzag mode is defined as the DLD critical size.^[^ ^254^, ^255^ ^]^ Therefore, the separation of target bioparticles with other components can be realized by controlling a desired critical size, which depends on multiple design parameters, including the row shift fraction, pillar gap, and the pillar diameter.^[^ ^45^, ^256^ ^]^

Li et al.^[^ ^47^ ^]^ proposed a multistage DLD microfluidic device to separate CTCs from blood sample based on differences in size and stiffness (Figure 11b). The platform consists of two‐array DLD chip, cone channel chip, and Raman analysis system. In order to optimize isolation performance metrics, such as purity, throughput, and array length, which are influenced by design parameters, they developed a droplet‐shaped pillar array through optimization design rather than empirical methods. This design enhancement improved fluid regulation, resulting in a 21.25% reduction in critical size and a 28% increase in throughput. Three types of cells in blood sample were isolated in the DLD chip based on size differences, and the mixed solution of CTCs and WBCs was further purified in the cone channel, exploiting the smaller stiffness of CTCs.^[^ ^257^ ^]^ Wunsch et al. developed a DLD microfluidic chip with nanoscale pillar arrays for isolating exosomes and colloids down to 20nm (Figure 11c).^[^ ^69^ ^]^ *Pe* number, defined as the ratio of the time required for particles to diffuse in a length of pillar gap to the time required for traversing the same distance in the advecting flow, is a critical parameter for DLD separation techniques at the nanoscale. As the pillar gap changes from the micron to the nanoscale, the *Pe* number decreases rapidly, causing diffusion to dominate over displacement and making particle motion indeterminate. In order to determine whether DLD method remains effective at the nanoscale, nanoscale DLD arrays with uniform gap sizes ranging 25–235 nm were fabricated using manufacturable silicon processes. Experimental results demonstrated that these nanoscale DLD arrays could successfully separate exosomes and nanoparticles with diameters ranging from 20 nm to 110 nm, and the partial displacement mode between bump and zigzag was proposed to characterize the competitive effect of deterministic displacement and diffusion. The migration angle for zigzag mode and bump mode are zero and maximum, respectively. In the partial displacement mode, the migration angle is a fraction of the maximum angle, as particles cannot fully migrate to the opposite side of the channel. Therefore, the partial displacement mode can be considered a lower‐efficiency version of the bump mode.

Although DLD is a versatile particle isolation method with high resolution for various biological applications, several challenges remain, including low throughput, pillar clogging, and high shear stress. The channel fluid resistance in DLD chips is comparatively higher than other isolating technologies due to the numerous pillar structures, which limits the scalability of DLD for processing large sample volumes. Particle adhesion and clogging present significant challenges for efficient isolation, as the disruption of streamlines near the clogged regions, alters the critical size of the original DLD chip.^[^ ^248^, ^258^ ^]^ In addition, bioparticles typically experience high shear stress, which may compromise the integrity.^[^ ^252^ ^]^

#### 2.2.4 Microfiltration

Microfiltration is a size‐exclusive method commonly used to isolate the largest type of bioparticle in the complex sample.^[^ ^259^, ^260^ ^]^ When samples are sucked into the microfiltration chip through the negative pressure generated by a syringe pump, bioparticles smaller than the hole diameter can pass through the pores on the membrane and flow out from the designated outlets, while larger target bioparticles are hindered on the upstream side of the membrane.^[^ ^261^, ^262^ ^]^

**Figure 12:**
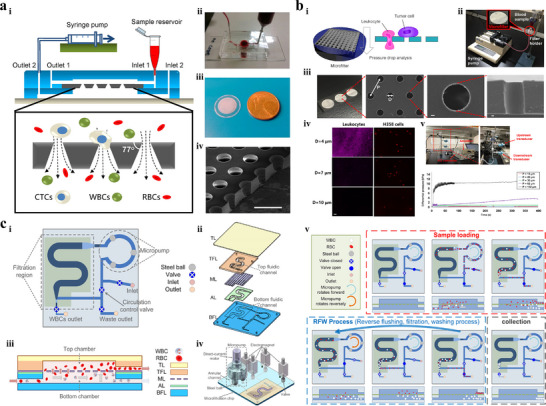
Microfiltration techniques. a) Microfluidic chip with integrated conical‐pore filter for isolating CTCs.^[^ ^260^ ^]^ Copyright 2014, Springer Nature. i) Schematic illustration of the system. ii) Prototype device. iii) The filter membrane. iv) The scanning electron microscope image of the conical pores array. Scale bar: 40 µm. b) Pressure‐sensing microfiltration device for isolating CTCs.^[^ ^265^ ^]^ Copyright 2022, American Chemical Society. i) Schematic illustration of the device. ii) Image of the experimental setup. iii) Photograph of the filter and scanning electron microscopy images of the pores. Scale bar: 1 µm. iv) Fluorescence images of the microfilter with different pore diameters after separating. v) The pressure sensing module (top) and pressure drop results (bottom) for different pore pitches. c) The microfiltration device integrating the rotary bidirectional micropump and the polycarbonate microporous membrane.^[^ ^266^ ^]^ Copyright 2016, Springer Nature. i) Schematic of the microfiltration chip. ii) Exploded views of the multilayer structure. iii) The cross‐section of the filtration region. iv) Schematic illustration of the experimental platform. v) Working procedure for separating WBCs.

The main challenges of the microfiltration technique are contamination and the coalescence of multiple pores.^[^ ^263^, ^264^ ^]^ These issues lead to a high retention ratio of non‐target bioparticles due to excessively small pore sizes, as well as the undesired escape of target bioparticles. To address these problems, Tang et al.^[^ ^260^ ^]^ designed a 2D microfilter with high density and conical‐shaped pores to optimize flow rate and transfilter pressure, thereby improving the clearance of interfering cells (**Figure** **12**a). Moreover, the microfilters were fabricated using micro‐aspiration aided molding of UV curable polyethylene glycol diacrylate, offering several advantages including the mechanical and thermodynamic stability, optical transparency, and biocompatibility. The pore pattern on membrane and the pressure difference between upstream and downstream sides of the membrane are also the critical factors for microfiltration‐based separation. Onoshima et al.^[^ ^265^ ^]^ investigated the influence of two parameters, including pore diameter and pressure drop, on the performance of CTCs sorting (Figure 12b). When the pore diameter exceeded 10 µm, the number of CTCs and WBCs captured on the microfiltration membrane significantly decreased. In contrast, numerous CTCs and WBCs were captured on membrane if the diameter of pore is smaller than 5 µm. Therefore, pores with 7 µm diameter were chosen to capture the target CTCs and filter out blood cells. In addition, they discovered that the pressure drop is positively correlated with the pore pitch, and further concluded that CTCs and WBCs can be effectively isolated when the pore pitch is smaller than 30 µm. Cheng et al.^[^ ^266^ ^]^ proposed a microfiltration device featuring with bidirectional micropump to effectively reduce membrane clogging by flushing the pores back and forth (Figure 12c). The flexible PDMS membrane covering the annular channel in filtration region was pressed by three steel balls connected to a motor. The peristaltic deformation of the PDMS membrane, generated by the circular movement of steel balls, was utilized to drive the fluid in the channel.^[^ ^267^ ^]^ The separation process began with loading the sample, in which the channel was rinsed by injecting buffer, followed by loading a volume of human whole blood sample and buffer. The fluid was drive to flow forward by controlling the motor to rotate counterclockwise, and the WBCs were captured by the microporous membrane, while partial RBCs and platelets passed through the membrane and flowed into the waste outlet. In order to further remove interfering components in blood sample, the device operated repeatedly in multiple modes, including reverse flushing, circulating filtration, and washing, until most non‐target cells were filtered out. The clogging issue was prevented by activating reverse flushing, which was triggered by clockwise rotation of the motor, pushing cells trapped in the micropores back into the bottom chamber. The ideal separation effect was achieved after several repeated operations, and WBCs were finally collected from corresponding outlet.

Microfiltration is a cost‐effective technique for isolating target cells from samples due to the integration of commercially available polycarbonate microporous filter membranes.^[^ ^262^ ^]^ In addition, the microfiltration system can directly process the undiluted whole blood,^[^ ^266^ ^]^ avoiding the complex preprocessing operations required by other sorting methods. However, microfiltration techniques are typically utilized to isolate micron‐scale bioparticles, such as CTCs and WBCs,^[^ ^268^, ^269^, ^270^ ^]^ and it is challenging to reliably separate nanoscale bioparticles. High transfilter pressure can cause excessive cellular stress,^[^ ^260^, ^271^ ^]^ compromising the viability and integrity of the cells separated by microfiltration, which is detrimental to downstream bioanalysis. Furthermore, to prevent clogging of the filter membrane pores, the flow rate or single injection volume needs to be limited,^[^ ^265^, ^266^ ^]^ which significantly restricts the separation throughput.

#### 2.2.5 PFF

PFF microfluidic chip consists of a pinched segment and a broadened segment. By adjusting the flow rates from two inlets, bioparticles of different sizes are focused against one sidewall in the pinched segment, regardless of their dimensions.^[^ ^272^, ^273^ ^]^ The direction of the hydrodynamic forces, exerted on smaller and larger particles about to enter the broadened segment from pinched segment, are significantly dependent on the centroid positions of particles within the spreading flow profile.^[^ ^274^, ^275^ ^]^ Consequently, the insignificant difference in particle centroid positions in the pinched segment is markedly amplified in the broadened segment, causing the isolation of particles of different sizes in the direction perpendicular to the flow.^[^ ^276^ ^]^ A smaller width of the pinched segment can significantly improve the isolation efficiency of the PFF device, but it may also lead to issues such as particle clogging in the pinched segment.^[^ ^274^ ^]^ Therefore, various PFF techniques have been proposed to improve the separation efficiency without reducing the width of the pinched segment. For example, the asymmetric PFF (AsPFF) with an additional drain channel was proposed to optimize separating effect by inducing the asymmetric distribution of the pinched flow in a broadened segment.^[^ ^273^, ^275^ ^]^

**Figure 13:**
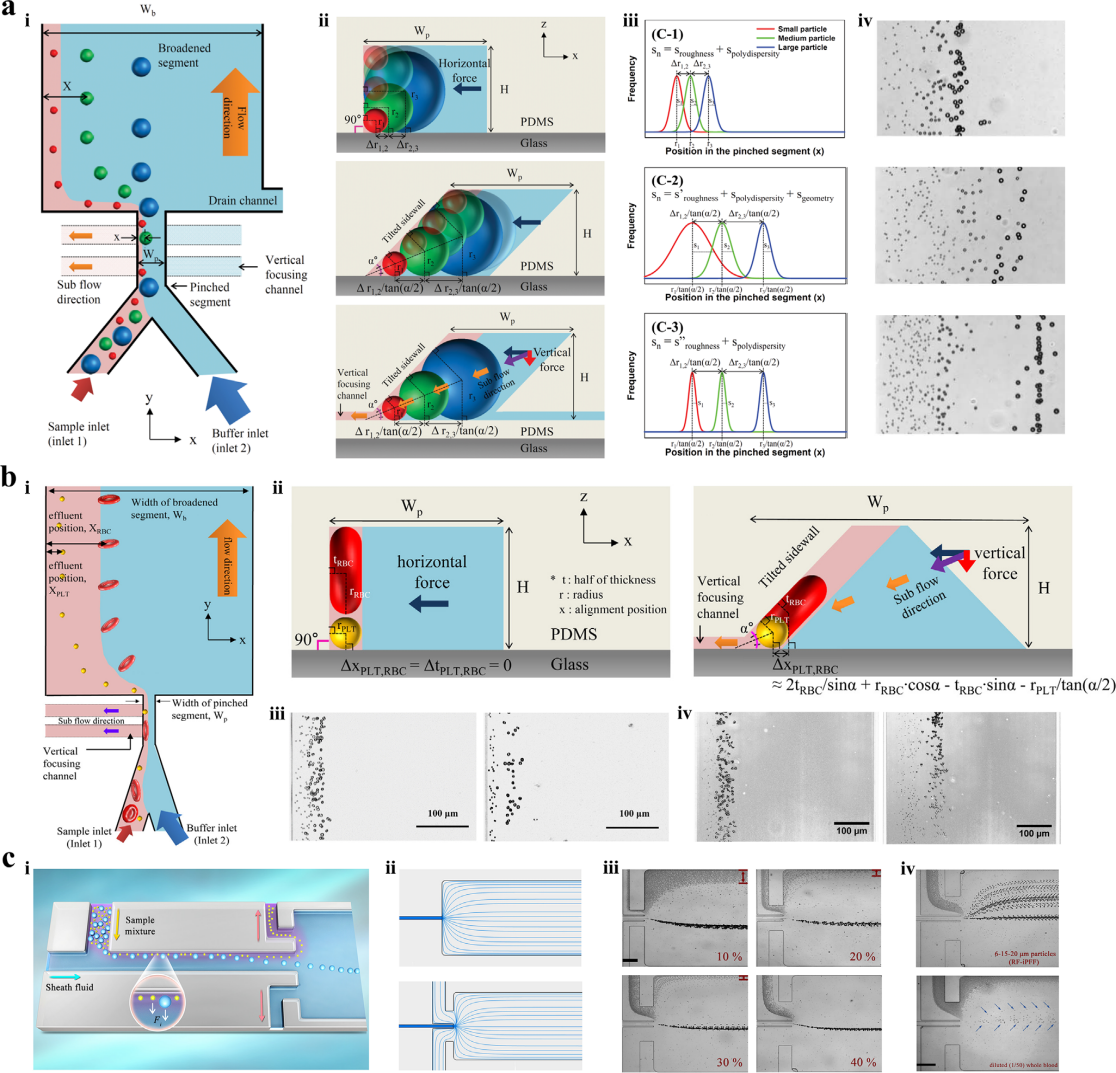
PFF techniques. a) AsPFF device with tilted sidewall and vertical focusing channels.^[^ ^277^ ^]^ Copyright 2013, Royal Society of Chemistry. i) Schematic diagram of the device. ii) Distribution of particles in pinched channels with different cross‐sections: normal AsPFF (top), tilted‐wall AsPFF (middle), and tilted‐wall AsPFF with vertical focusing channels (bottom). iii) The estimated position distribution of particles in pinched channels with different cross‐sections. iv) Experimental results of particle trajectories in broadened segments. b) Tilted‐wall PFF for isolating disc‐shaped particles.^[^ ^278^ ^]^ Copyright 2017, Elsevier. i) Schematic illustration of the isolation of RBCs and platelets. ii) The distribution of RBCs and platelets in pinched segments of normal PFF and tilted‐wall PFF. iii) Trajectories of 2 µm spherical particles and 2 µm thick, 4 µm diameter disc‐shaped particles in the broadened segment. iv) Trajectories of RBCs and platelets in the broadened segment. c) Reverse flow enhanced inertia PFF device.^[^ ^279^ ^]^ Copyright 2023, Royal Society of Chemistry. i) Schematic diagram of the isolation process. ii) The simulated streamlines in reverse flow enhanced inertia PFF device and traditional PFF device. iii) The effects of reverse flow under different opening size. Scale bar: 500 µm. iv) Results of separating three kinds of particles simultaneously (top) and isolating tumor cells from whole blood (bottom). Scale bar: 500 µm.

In order to further improve the sorting efficiency, Nho et al.^[^ ^277^ ^]^ designed an AsPFF with two additional features, including the tilted sidewall and vertical focusing channels (**Figure** **13**a). This AsPFF device enhanced isolation performance through following two aspects: increasing the horizontal position differences of particles with different sizes by adopting the tilted sidewall in the pinched segment, and reducing the distribution width of particles of the same type through the vertical focusing channels. A portion of the fluid in the pinched channels was diverted from the vertical focusing channels, causing a downward force on particles. Combined with the horizontal force generated by the different flow rates from two inlets, a diagonal force was formed to drive particles into the corner of pinched segment. The centroid position differences of particles with distinct sizes in the pinched segment were amplified by the tilted sidewalls, and the magnification factor is dependent on the tilting angle (the magnification factor is 2.14 for the tilting angle of 50°). In addition, Nho et al.^[^ ^278^ ^]^ further verified the capability of this tiled‐wall PFF device with vertical focusing channels for shape‐based isolation of particles (Figure 13b). The tilted‐wall PFF device determines the centroid position of disc‐shaped particle based on the lengths of two axes, rather than relying solely on the minor axis length. Therefore, the tilted‐wall PFF device can separate spherical particles with a radius similar to the thickness of disc‐shaped particles, which was verified by the experiments on the separation of spherical and disc‐shaped polystyrene particles, as well as the separation of platelets and RBCs. Wang et al.^[^ ^279^ ^]^ proposed a reverse flow enhanced inertia PFF to improve the separation efficiency by introducing reverse flow channels (Figure 13c). Smaller particles were pulled backward into the reverse flow from the stream in the broadened segment, where larger particles were located, weakening the adverse effect of inertia: the distribution range of smaller particles in broadened segment expands and shifts to the larger particles as the flow rate increases.^[^ ^280^ ^]^ The experimental results showed that 6 µm polystyrene particles were almost filtered out from particle mixtures (6 µm/20 µm) when the opening size of reverse flow increased to 20% of the width of the broadened segment. Additionally, tumor cells were also successfully isolated from diluted whole blood under optimal conditions.

PFF is a promising technique for isolating non‐spherical bioparticles, such as RBCs, bacteria, and spermatozoa,^[^ ^46^, ^278^, ^281^ ^]^ based on the shape differences. This is because the centroid position of particles in a pinched segment is dependent on the dimensions in multiple direction, and non‐spherical particles exhibit more complex motion patterns that shift their trajectories away from the sidewalls.^[^ ^281^, ^282^ ^]^ However, as the flow rate increases, the distribution bands of smaller particles in the broadened segment expand and shift to the bands of larger particles. As a result, the sample processing throughput of PFF techniques is typically controlled at the level of µL·h^−1^ to guarantee separation purity. In addition, PFF devices currently cannot sort particles at submicron resolution, limiting their application in other research fields, such as the separation of exosomes.

### 2.3 Hybrid Methods

In order to integrate the advantages of active and passive methods, hybrid separation systems designed by combining multiple typical sub‐modules or implanting active control components into the passive platforms have been proposed.^[^ ^283^, ^284^, ^285^ ^]^ According to the type of active components in hybrid systems, they can be divided into four categories: magnetophoresis‐assisted,^[^ ^105^, ^286^, ^287^ ^]^ dielectrophoresis‐assisted,^[^ ^106^, ^288^, ^289^ ^]^ acoustophoresis‐assisted,^[^ ^290^, ^291^ ^]^ and optophoresis‐assisted hybrid methods.^[^ ^292^ ^]^ Several typical systems for each of these hybrid methods are discussed in the following section.

#### 2.3.1 Magnetophoresis‐Assisted Hybrid Methods

**Figure 14:**
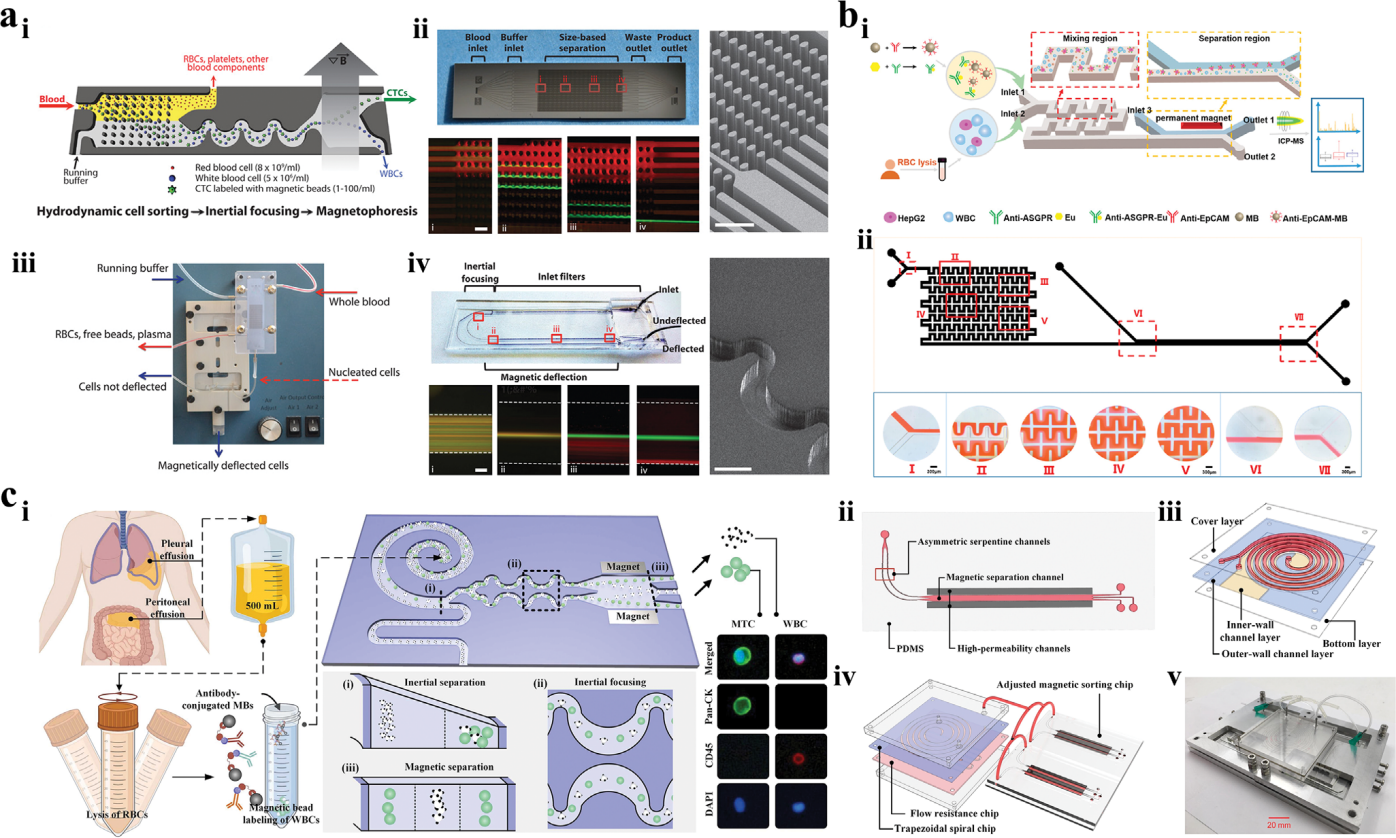
Magnetophoresis‐assisted hybrid methods. a) Integrated device with DLD module, serpentine channel, and magnetophoresis module.^[^ ^9^ ^]^ Copyright 2013, American Association for the Advancement of Science. i) Schematic illustration of the system. ii) The separation of 2 µm (red) and 10 µm (green) particles in DLD module. iii) Prototype platform. iv) Cell focusing and magnetophoretic sorting. b) All‐in‐One Microfluidic Chip.^[^ ^293^ ^]^ Copyright 2023, American Chemical Society. i) Schematic illustration of the device. ii) The distribution of red ink at different positions of the serpentine channel. c) Integrated device with trapezoidal spiral channel, serpentine channel, and magnetophoresis module.^[^ ^28^ ^]^ Copyright 2023, Elsevier. i) Schematic illustration of the system. ii) The planar diagram of magnetophoresis module. iii) The diagram of trapezoidal spiral channel. iv) Schematic illustration of the integrated device. v) Prototype device.

Magnetophoresis‐assisted hybrid isolation systems typically utilize magnetic components to further purify target particles. Ozkumur et al.^[^ ^9^ ^]^ designed an integrated system composed of pillar array, asymmetric serpentine microchannel, and magnetophoresis module (**Figure** **14**a), which are used to remove RBCs and platelets, focus CTCs and WBCs, and isolate CTCs from WBCs, respectively. RBC lysis was omitted, as the DLD module could effectively deplete almost small‐size blood components. The target CTCs were then specifically and precisely isolated from the remaining solution using the magnetophoresis technique. In order to further simplify pretreatment steps and reduce the risk of cell contamination and loss, Xu et al.^[^ ^293^ ^]^ proposed an all‐in‐one microfluidic chip to integrate multiple functions, such as immunolabeling, focusing, and magnetic separation of CTCs (Figure 14b). The mixing zone, consisting of repeated serpentine channel units (>60), was introduced for the thorough mix, and target HepG2 cells were labeled with anti‐ASGPR‐Eu and specifically captured by anti‐epithelial cell adhesion molecule‐modified magnetic beads via antigen−antibody recognition. Similar to the integrated device proposed by Ozkumur et al., Ni et al.^[^ ^28^ ^]^ replaced the DLD module with a trapezoidal spiral channel and selected WBCs as the magnetic beads labeled targets to improve the throughput and purity of tumor cells (Figure 14c).

#### 2.3.2 Dielectrophoresis‐Assisted Hybrid Methods

**Figure 15:**
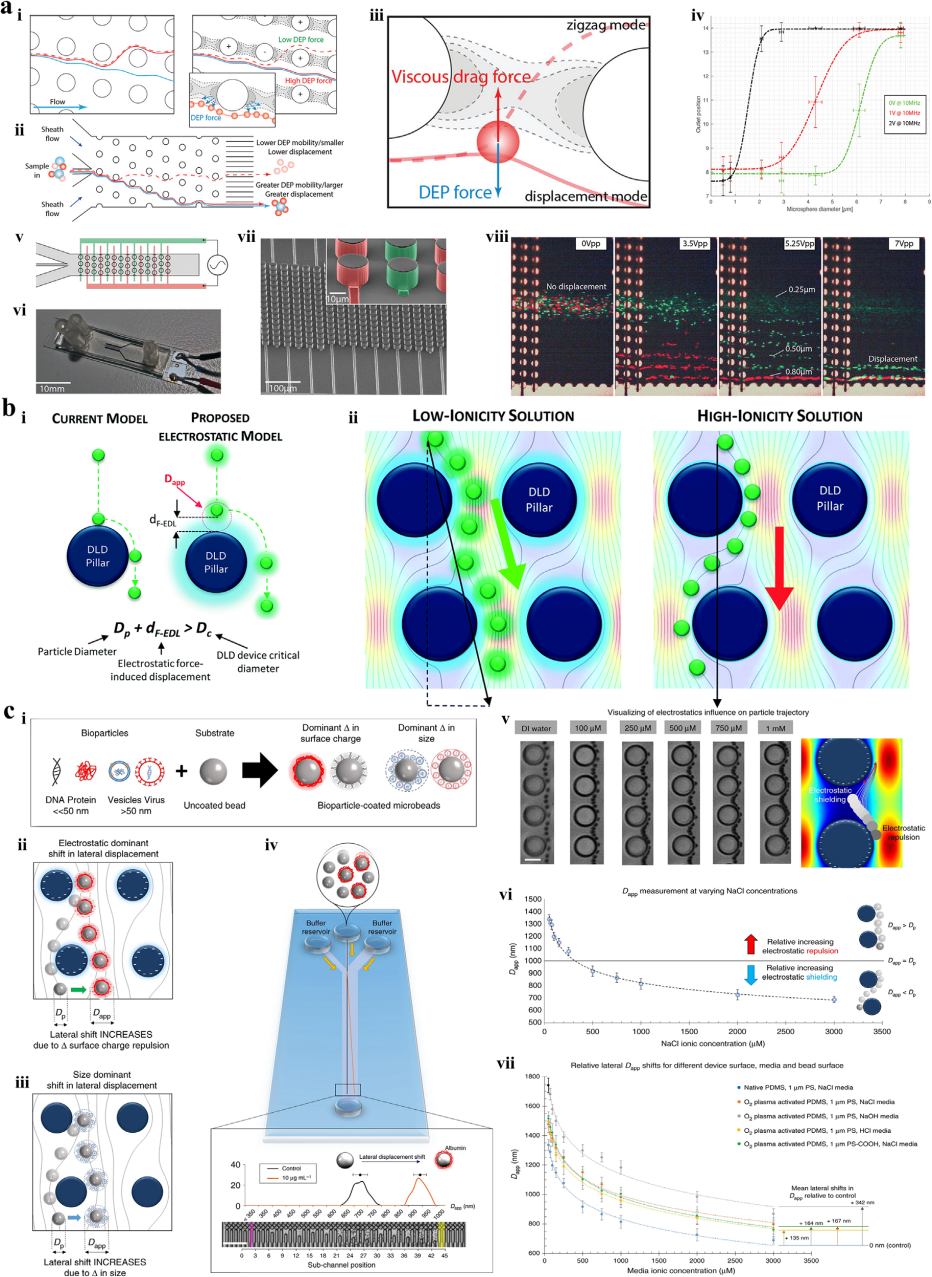
Dielectrophoresis‐assisted hybrid methods. a) Active DLD device with electrically connected metal‐coated pillars.^[^ ^295^ ^]^ Copyright 2019, Wiley. i) Trajectories of particles of different sizes in traditional DLD device (left) and the proposed active DLD device (right). ii) Schematic illustration of the active DLD chip. iii) The lateral components of the dielectrophoretic force and viscous drag force. iv) Outlet positions of microspheres of different sizes under distinct applied voltage input signals. v) Schematic of device design. vi) Prototype platform. vii) Scanning electron microscope image of the active pillar region. viii) Tunable isolation of 0.25, 0.5, and 0.8 µm microspheres under different voltages with frequency of 10 MHz. b) The regulation mechanism of electrostatic force‐modulated DLD platform.^[^ ^289^ ^]^ Copyright 2016, Royal Society of Chemistry. i) The interaction between pillars and particles in traditional and the proposed DLD device. ii) Particle trajectories in the fluid media with different ionic concentrations. c) The electrostatic force‐modulated DLD platform for isolating nano‐sized bioparticles.^[^ ^296^ ^]^ Copyright 2018, Springer Nature. i) Adhering bioparticles onto the polymer beads. ii) Electrostatic dominant shift in lateral displacement. iii) Size dominant shift in lateral displacement. iv) Schematic illustration of the device. v) Particle trajectories under different NaCl concentrations. vi) Apparent diameters under different NaCl ionic concentrations. vii) Apparent diameters shift with device surface charges, bead surface charges, and pH of the media.

The mainstream dielectrophoresis‐assisted hybrid methods refer to embedding electrodes into the microstructure of original microfluidic chips.^[^ ^106^, ^288^, ^294^ ^]^ Beech et al.^[^ ^295^ ^]^ proposed an active DLD chip consisting of cylindrical metal‐coated SU‐8 pillars connected by an underlying metal layer (**Figure** **15**a). This design creates high electric field gradients between adjacent pillars across a wide range of frequencies, generating sufficient dielectrophoretic force on polarizable particles. The dielectrophoretic force, which depends on particle volume, the polarizabilities of the fluid and particles, and the gradient of the electric field, can compel particles to transition from the zigzag motion mode to the bump motion mode. Therefore, the critical size of the active DLD chip can be tuned simply by controlling the output signals of the function generator, allowing different sorting tasks to be flexibly performed using this dielectrophoresis‐assisted DLD system. Based on the above method, another expanding technology was proposed to separate nanoparticles without the requirement of external electric field.^[^ ^289^ ^]^ The electrostatic force in DLD device is non‐trivial and may significantly affect the motion of particles, and the electrostatic interaction between pillars and particles can be characterized by the thickness of the electric double layer, which is dependent on the ionic concentration of NaCl buffer (Figure 15b). Zeming et al.^[^ ^296^ ^]^ investigated a technique for isolating nano‐sized bioparticles by DLD device, based on the changes in size and electrostatic charges of polymer particles after capturing target bioparticles (Figure 15c). Electrostatic interactions dominate the motion of smaller particles, while physical particle size plays the leading role for larger particles. The apparent diameter decreases as the ionic concentration increases, becoming comparable to the actual diameter of the polymer particles when the NaCl concentration is about 350 µM. Further, they concluded that three parameters including device surface charges, bead surface charges, and pH of the media are the main factors for the shifts of apparent diameter.

#### 2.3.3 Acoustophoresis‐Assisted Hybrid Methods

**Figure 16:**
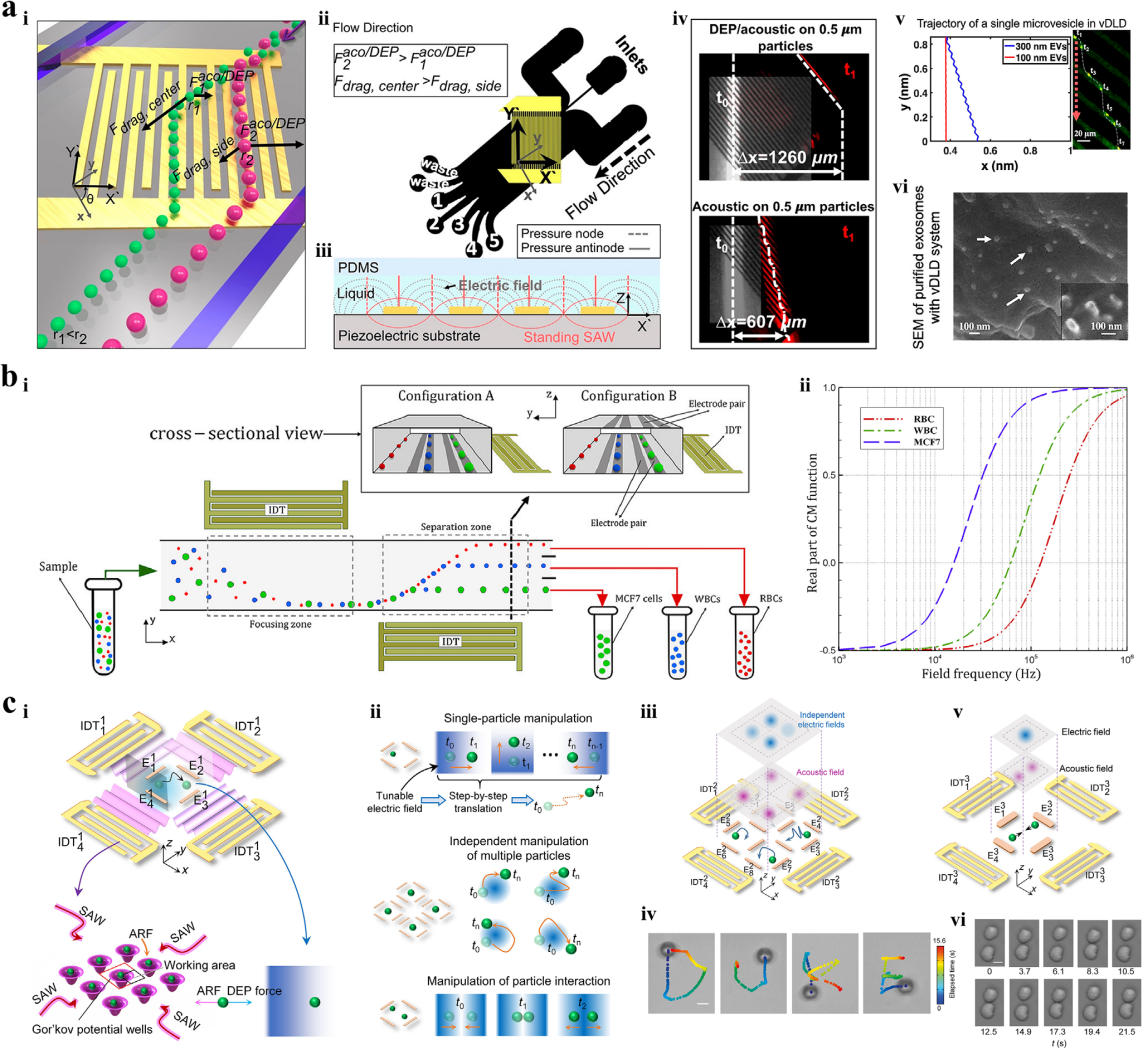
Acoustophoresis‐assisted hybrid methods. a) Deterministic sorting system for isolating nanoparticles.^[^ ^298^ ^]^ Copyright 2021, American Chemical Society. i) Schematic illustration of the working principle. ii) Diagram of the device. iii) Acoustic and electric fields between electrodes. iv) Particle (red) trajectories under different conditions. v) Simulated trajectories of 100 and 300 nm extracellular vesicles in the DEP/acoustic force fields. vi) The scanning electron microscope image of the separated exosomes. b) Microfluidic chip for CTCs isolation by combining acoustophoresis and dielectrophoresis methods.^[^ ^299^ ^]^ Copyright 2021, Elsevier. i) Schematic illustration of the platform. ii) The real part of Clausius–Mossotti factor of different cells. c) Acousto‐dielectric tweezers for manipulating multiple particles.^[^ ^130^ ^]^ Copyright 2024, American Association for the Advancement of Science. i) Schematic illustration of the device. ii) Three manipulation modes of particles. iii) Mechanism of the independent and simultaneous manipulation of multiple particles. iv) Four particles were transported to trace different letters D, U, K, and E simultaneously. v) Mechanism of controlling the distance between two cells. vi) Experimental results of invertible pairing and separation of two cells.

Acoustophoresis‐assisted hybrid systems typically use IDTs to generate the BAW and SAW, which pre‐focus particles before separation or introduce acoustic radiation force to enhance the isolation efficiency.^[^ ^297^ ^]^ Tayebi et al.^[^ ^298^ ^]^ proposed a deterministic sorting system for isolating nanoparticles and extracellular vesicles by combining the electric and acoustic field (**Figure** **16**a). IDTs were implemented within the microchannels to create an acoustic field composed of SSAW and TSAW, which exerted acoustic radiation force on particles. In addition, nonuniform electric fields formed above the electrodes induced the dielectrophoretic force. Employing the dielectrophoretic force in acoustic‐based separating system introduces additional control parameters, such as permittivity and conductivity, which allows for more diversified manipulation of particles with varying physical and electric properties. Larger particles migrated further in the *x* direction due to the greater comprehensive effect caused by acoustic and dielectrophoretic forces, thereby realizing the isolating of particles. Derakhshan et al.^[^ ^299^ ^]^ developed a novel microfluidic chip for CTCs isolation utilizing the combination of acoustophoresis and dielectrophoresis methods (Figure 16b). Bioparticles were firstly focused on the sidewall of the channel by the IDTs on the opposite side, and the aligned cells were then sorted in the separation zone. According to the real part of Clausius–Mossotti factor of different cells, CTCs and the other two types of cells experienced the positive and negative dielectrophoresis under an electrode frequency of 55 kHz, respectively. Therefore, when cells were pushed to the position of first electrode pair by TSAW, CTCs were attracted and flowed along the electrode pair. In contrast, RBCs and WBCs passed through the first electrode pair easily and were separated by second electrode pair at an electrode frequency of 100 kHz based on the similar sorting principle. For more precise and flexible particle manipulation, including tracing designated paths, pairing, and separation, Shen et al.^[^ ^130^ ^]^ investigated the acousto‐dielectric tweezers composed of two pairs of IDTs positioned orthogonally along two axes, four‐port electrodes deposited on the lithium niobate substrate, and the polydimethylsiloxane–based microchamber ((Figure 16c). The IDTs were used to form SSAW with a grid latticelike distribution of Gor'kov potential wells, which trapped particles through acoustic radiation forces. The four electrodes created a nonuniform electric field, applying a negative dielectrophoretic force on the particles. Therefore, the single particle was trapped at an equilibrium position between the center of the Gor'kov potential well and the location with the lowest electric field magnitude, depending on the competition between acoustic radiation and dielectrophoresis forces. Furthermore, the dielectrophoresis force could be adjusted by adjusting the voltages and frequencies, allowing for the flexible control over the motion and position of particles.

#### 2.3.4 Optophoresis‐Assisted Hybrid Methods

**Figure 17:**
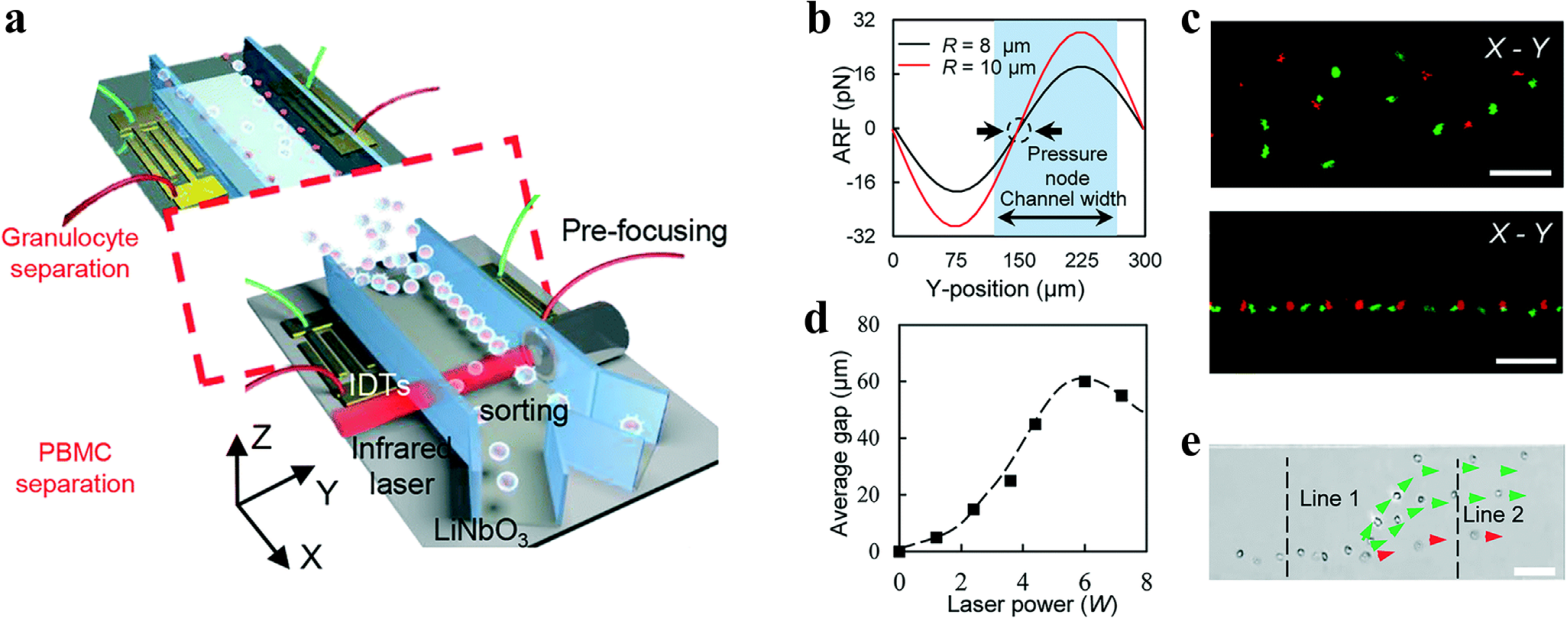
Optophoresis‐assisted hybrid methods.^[^ ^292^ ^]^ Copyright 2018, Royal Society of Chemistry. a) Schematic of the acoustic‐optical separating device. b) The magnitude of acoustic radiation force along *y* direction. c) Trajectories of cells (monocytes and lymphocytes were dyed red and green, respectively) in pre‐focusing region when SSAW was off and on. d) The gap between two types of cells varies with laser power. e) Experimental results of cell trajectories in the sorting region.

The separation mechanism of optophoresis‐assisted hybrid system is similar to that of optophoresis device, with the key difference being that the hybrid system incorporates a pre‐focusing module, rather than relying on sheath fluid to focus bioparticles into a narrow bundle. Hu et al.^[^ ^292^ ^]^ proposed a hybrid acoustic‐optical microfluidic chip for precisely separating the leukocyte subpopulation. Although lymphocytes and monocytes overlap in density and dimension, they exhibit a significant difference in refractive index, which forms the basis for the accurate optical isolation of these cells which are pre‐focused by SSAW (**Figure** **17**).

## 3 Performance Comparison of Isolating Methods

Various isolating methods have been widely employed to separate micro/nano‐scaled bioparticles, such as CTCs, exosomes, bacteria, WBCs, platelets, and viruses. In order to comprehensively evaluate the performance of each isolating technique, six critical parameters, including purity, recovery rate, throughput, resolution, size, and convenience, are introduced for comparison (**Table** **1**). These research data clearly highlight the advantages and limitations of each technique (**Table** **2**), offering researchers a broader perspective and providing a foundation for the future optimization, innovation, and cross‐disciplinary fusion of sorting technologies.

**Table 1:** Performance comparison of various isolating techniques.

Isolation methods	Targets	Purity	Recovery rate	Throughput	Resolution	Size	Convenience
Positive magnetophoresis	CTC^[^ ^78^, ^80^, ^85^ ^]^	≈80%	≈98%	≈2 mL·h^−1^	≈5 µm	≈5 cm^2^	Low
	Exosome^[^ ^88^, ^300^, ^301^ ^]^	≈70%	≈80%	≈4 mL·h^−1^	≈300 nm	≈7 cm^2^	
	Bacterium^[^ ^89^, ^302^, ^303^ ^]^	—	≈90%	≈20 mL·h^−1^	—	≈50 cm^2^	
	WBC^[^ ^70^, ^304^, ^305^ ^]^	≈90%	—	≈1.2 mL·h^−1^	≈3 µm	≈5 cm^2^	
	Virus^[^ ^71^, ^306^, ^307^ ^]^	—	≈90%	≈6 mL·h^−1^	—	≈10 cm^2^	
Negative magnetophoresis	CTC^[^ ^97^, ^100^ ^]^	≈30%	≈80%	≈1 mL·h^−1^	≈5 µm	≈12 cm^2^	Medium
	Bacterium^[^ ^98^ ^]^	—	≈95%	≈0.1 mL·h^−1^	≈6 µm	≈12 cm^2^	
Dielectrophoresis	CTC^[^ ^114^, ^308^ ^]^	≈80%	≈90%	—	≈10 µm	≈4 cm^2^	Medium
	Exosome^[^ ^116^, ^119^ ^]^	—	—	≈0.1 mL·h^−1^	≈50 nm	≈0.2 cm^2^	
	Bacterium^[^ ^118^, ^309^, ^310^ ^]^	—	≈50%	≈1 mL·h^−1^	≈4 µm	≈20 cm^2^	
	WBC^[^ ^115^ ^]^	≈85%	—	≈0.2 mL·h^−1^	≈1 µm	≈15 cm^2^	
	Virus^[^ ^311^ ^]^	—	—	≈0.012 mL·h^−1^	—	—	
Acoustophoresis	CTC^[^ ^142^, ^146^ ^]^	—	≈80%	≈0.3 mL·h^−1^	≈3 µm	≈30 cm^2^	Medium
	Exosome^[^ ^3^, ^147^ ^]^	≈95%	≈80%	≈0.25 mL·h^−1^	≈250 nm	≈10 cm^2^	
	Bacterium^[^ ^6^, ^17^ ^]^	≈95%	≈80%	≈5 µL·h^−1^	≈4 µm	≈10 cm^2^	
	Platelet^[^ ^312^, ^313^, ^314^ ^]^	≈85%	≈90%	≈10 mL·min^−1^	≈5 µm	≈20 cm^2^	
	Virus^[^ ^12^, ^315^ ^]^	≈90%	≈85%	≈1 mL·h^−1^	≈70 nm	≈15 cm^2^	
Optophoresis	CTC^[^ ^35^, ^173^ ^]^	≈90%	≈90%	≈0.1 mL·h^−1^	≈5 µm	≈3 cm^2^	Medium
FAS	CTC^[^ ^61^, ^175^, ^316^ ^]^	≈90%	≈90%	≈40 µL·h^−1^	—	≈40 cm^2^	Low
	WBC^[^ ^179^ ^]^	—	≈80%	≈3 mL·h^−1^	—	≈24 cm^2^	
IMF	CTC^[^ ^204^, ^205^, ^218^ ^]^	≈80%	≈90%	≈3 mL·min^−1^	≈2 µm	≈64 cm^2^	Medium
VEM	CTC^[^ ^22^, ^229^ ^]^	—	≈80%	≈2 mL·h^−1^	≈5 µm	≈10 cm^2^	High
	Exosome^[^ ^53^, ^233^ ^]^	≈90%	≈80%	≈0.2 mL·h^−1^	≈400 nm	≈5 cm^2^	
	Bacterium^[^ ^54^, ^317^, ^318^ ^]^	≈90%	≈80%	≈0.03 mL·h^−1^	≈3 µm	≈15 cm^2^	
DLD	CTC^[^ ^319^, ^320^, ^321^ ^]^	≈90%	≈90%	≈0.6 mL·h^−1^	≈4 µm	≈60 cm^2^	Medium
	Exosome^[^ ^69^, ^322^ ^]^	—	—	≈12 nL·h^−1^	≈60 nm	≈15 cm^2^	
Microfiltration	CTC^[^ ^260^, ^262^, ^323^ ^]^	≈90%	≈90%	≈10 mL·h^−1^	≈5 µm	≈10 cm^2^	High
	WBC^[^ ^261^, ^266^ ^]^	≈90%	≈30%	≈1 mL·h^−1^	≈3 µm	≈5 cm^2^	
PFF	CTC^[^ ^279^, ^324^ ^]^	—	≈90%	≈0.1 mL·h^−1^	≈5 µm	≈6 cm^2^	Medium
	Bacterium^[^ ^46^ ^]^	—	≈80%	≈0.03 mL·h^−1^	≈5 µm	≈2 cm^2^	
	Platelet^[^ ^278^ ^]^	—	—	≈0.1 mL·h^−1^	≈2 µm	≈1 cm^2^	
Magnetophoresis‐assisted hybrid methods	CTC^[^ ^28^, ^286^, ^293^ ^]^	≈70%	≈90%	≈3 mL·min^−1^	≈5 µm	≈64 cm^2^	Low
Dielectrophoresis‐assisted hybrid methods	CTC^[^ ^285^, ^325^, ^326^ ^]^	—	≈90%	≈0.1 mL·h^−1^	≈5 µm	≈15 cm^2^	Medium
	Exosome^[^ ^296^, ^327^ ^]^	—	—	≈1.3 µL·h^−1^	≈300 nm	—	
	Bacterium^[^ ^328^ ^]^	—	≈80%	≈30 mL·h^−1^	—	≈10 cm^2^	
Acoustophoresis‐assisted hybrid methods	CTC^[^ ^297^, ^299^ ^]^	—	≈70%	≈1.2 mL·h^−1^	≈5 µm	—	Medium
	Exosome^[^ ^298^ ^]^	≈95%	≈80%	≈0.1 mL·h^−1^	≈200 nm	≈1cm^2^	
	WBC^[^ ^292^ ^]^	≈95%	≈95%	≈0.2 mL·h^−1^	—	—	

**Table 2:** The advantages and limitations of various isolating methods.

Isolation methods	Advantages	Limitations
Positive magnetophoresis	High sorting resolution Capability of isolating micro‐ and nano‐sized bioparticles Good biocompatibility High throughput	Time‐consuming labeling and washing steps Large dimension due to the requirement of external magnetic device Difficulty in further integration and parallel design due to the coupling effect of multiple magnetic fields Low reliability caused by bioparticle heterogeneity Low convenience
Negative magnetophoresis	Label‐free isolation Low cost	Low purity Cytotoxicity of magnetic fluid Inconvenience for observing Incapability of isolating nano‐sized bioparticles Difficulty in further integration and parallel design due to the coupling effect of multiple magnetic fields
Dielectrophoresis	High sorting resolution Capability of isolating micro‐ and nano‐sized bioparticles Label‐free isolation Tunable separation	Low throughput Requirement of sophisticated and expensive system Cytotoxicity of ionic buffer Short service life caused by electrochemical reactions Excessive joule heat
Acoustophoresis	High sorting resolution Capability of isolating micro‐ and nano‐sized bioparticles Label‐free isolation Tunable separation by constructing virtual microstructures Good biocompatibility Diversified manipulation of bioparticles	Low throughput Requirement of sophisticated and expensive system Intense acoustic streaming Difficulty in further integration and parallel design due to the large dimension of whole system
Optophoresis	Label‐free isolation Good biocompatibility	Low throughput Requirement of sophisticated and expensive system Incapability of isolating nano‐sized bioparticles Difficulty in further integration and parallel design due to the large dimension of whole system
FAS	High sorting resolution Tunable separation by fluorescent labeling Good biocompatibility	Low throughput Time‐consuming fluorescent labeling steps Incapability of isolating nano‐sized bioparticles Requirement of sophisticated and expensive system Difficulty in further integration and parallel design due to the large dimension of whole system
IMF	High throughput Simple structure Label‐free isolation Good biocompatibility Low cost	Requirement of lysing RBCs and diluting samples several tens of times Incapability of isolating nano‐sized bioparticles Difficulty in further integration and parallel design due to the large size of isolation module
VEM	Direct isolation of bioparticles from undiluted blood sample High sorting resolution Capability of isolating micro‐ and nano‐sized bioparticles Tunable separation by adjusting the concentration of synthetic polymer Easiness in further integration and parallel design due to the small size of isolation module Low cost	Time‐consuming steps for preparing viscoelastic fluids Negative effects of shear‐thinning and secondary flow Uncertain sorting mechanism due to the complex dynamics
DLD	High sorting resolution Capability of isolating micro‐ and nano‐sized bioparticles Label‐free isolation Good biocompatibility	High manufacturing costs Low throughput due to the high fluid resistance Unreliable sorting performance due to bioparticle blockage Damage of bioparticles caused by high stress
Microfiltration	Direct isolation of bioparticles from undiluted blood sample Easiness in further integration and parallel design due to the sample and small isolation module Label‐free isolation Low cost	Low throughput Incapability of isolating nano‐sized bioparticles Damage of bioparticles caused by high stress Unreliable sorting performance due to bioparticle blockage
PFF	Capability of isolating non‐spherical bioparticles Easiness in further integration and parallel design due to the sample and small isolation module Label‐free isolation Low cost	Low throughput Low purity Incapability of isolating nano‐sized bioparticles

Active separation techniques typically rely on sophisticated instruments to manipulate bioparticles in a precise, flexible, and on‐demand manner, which can reliably drive various types of bioparticles to move along the predetermined trajectories, further improving the purity of target particles. In addition, active methods can isolate bioparticles and their compounds, labeled with beads or fluorescence, based on multiple physical properties, not just geometric parameters. For example, dielectrophoresis and positive magnetophoresis techniques can respectively utilize the differences in electrical and magnetic properties to sort different types of bioparticles. Therefore, active techniques are powerful tools for isolating bioparticles with similar or even identical sizes. However, high manipulation accuracy is usually achieved at the cost of sacrificing sample loading speed, and the throughput cannot be improved by parallel design due to the coupling interference effects between physical fields of multiple submodules and the large dimension of the whole system. Moreover, active separation systems typically face challenges in compatibly integrating with downstream biological analysis modules, potentially leading to the loss or contamination of target bioparticles.

In contrast, passive isolation systems are usually small and simple, offering several advantages, including high throughput, low cost, and ease of integration, as they do not rely on external physical fields. Passive isolation devices also eliminate the need for time‐consuming and labor‐intensive labeling pretreatments since most passive methods separate target bioparticles based solely on size differences. However, this simplicity comes with trade‐offs: the separation resolution of passive methods is generally lower, and only a few passive techniques can isolate submicron bioparticles, such as exosomes and viruses. Moreover, passive methods struggle to provide tunable manipulation of bioparticles, making them less adaptable for isolating different targets using a fixed microdevice, unlike active methods such as dielectrophoresis and acoustophoresis.

Hybrid methods usually refer to connecting multiple typical subsystems in series or implanting active components into microstructures. Current hybrid separation systems face challenges in simultaneously improving multiple performance parameters, as each submodule performs optimally only under specific conditions. Furthermore, the disadvantages of each subunit often permeate the entire system. For example, the active DLD microfluidic chip, composed of metal‐coated pillars, significantly increases both the complexity and cost of the process.

## 4 Challenges and Perspectives

In this review, we have comprehensively introduced the state‐of‐the‐art techniques for isolating micro/nano‐scaled bioparticles from complex samples, detailing their basic structures, separation mechanisms, material selection, and biomedical applications. Although numerous isolation techniques have been developed and actually demonstrated superior performance in recent years, their clinical application still faces significant challenges, including complex fabrication, inefficient designs, and limited technological flexibility. However, with advancements in micro/nano‐fabrication technologies, novel functional materials, and artificial intelligence (AI), we anticipate that emerging technologies will overcome the current bottlenecks, enabling a smooth transition from laboratory research to actual clinical applications.

### 4.1 Challenges and Limitations

Addressing the challenges and limitations of isolation techniques is essential for advancing our understanding of disease mechanisms and developing new therapeutic methods. The following sections outline the key challenges in current microfluidic isolation technologies, focusing on three main aspects: fabrication, design, and flexibility.″

#### 4.1.1 Challenges in Fabrication

Several techniques have been proposed for fabricating microfluidic isolation platforms, including micromilling,^[^ ^329^ ^]^ micromachining,^[^ ^330^ ^]^ hot embossing,^[^ ^331^ ^]^ injection molding,^[^ ^332^ ^]^ and soft lithography.^[^ ^333^ ^]^ However, these traditional microfabrication methods suffer from multiple limitations, such as time‐consuming and expensive processes, instable quality, and low manufacturing precision,^[^ ^334^ ^]^ and these challenges are particularly pronounced when fabricating nanostructures. In addition, with the advancements in the research on nanoparticles,^[^ ^335^, ^336^, ^337^ ^]^ the demand for isolating nano bioparticles, such as exosomes, bacteria, and viruses, is becoming increasingly urgent.^[^ ^64^, ^338^ ^]^ Nevertheless, ultra‐precision nanostructures in microfluidic chips present remarkable manufacturing challenges.^[^ ^45^ ^]^ For instance, separating 20 nm particles requires a DLD microfluidic chip with structure accuracy of 25 nm, which relies on expensive deep ultraviolet lithography.^[^ ^69^ ^]^ The high requirements for ultrahigh‐precision instruments and practical experience limit the accessibility of the critical attributes of isolation devices, such as batch production, integration, and reliability.

#### 4.1.2 Challenges in Design

Personalized therapy and precision medicine put forward higher requirements for key isolation performances, such as throughput, purity, and recovery rate.^[^ ^339^, ^340^ ^]^ In order to achieve the optimal performance of isolation techniques, an ideal design scheme should be specified first. However, the current common practice is to design an initial isolation platform based on previous empirical knowledge, followed by studying the main influencing factors and performance variation patterns. Afterward, the device is then iteratively optimized based on experimental results.^[^ ^60^, ^222^ ^]^ After several rounds of design‐experiment‐optimization iteration, it may be possible to achieve expected goals or even still fail to meet the requirements, but anyway, the unnecessary investment of time and money in the iteration process is huge. In addition, microfluidic platform design can also be guided by theoretical calculations and numerical simulations,^[^ ^185^, ^341^, ^342^ ^]^ but the simplifying assumptions in theoretical analysis significantly restrict their generalizability. Furthermore, the numerical models are error‐prone and time‐consuming when dealing with complex flow dynamics.^[^ ^343^, ^344^ ^]^ As a result, the efficient design of microfluidic chips remains one of the key challenges hindering the rapid advancement and practical application of isolation techniques.

#### 4.1.3 Challenges in Flexibility

The excessively wide size distribution of bioparticles is another major obstacle to the widespread application of isolation techniques.^[^ ^345^ ^]^ For example, the sizes of exosome and viruses are ≈100 µm, while that of CTCs is ≈15 µm. Therefore, flexibility, which means tunable manipulation, is another key attribute of isolation platforms, in addition to six performance parameters.^[^ ^12^ ^]^ In many cases, it is desirable to separate different types of target bioparticles using the same device to cater for requirements of various applications. However, most current techniques are limited to specific application scenarios. For example, a microfluidic chip can separate MCF‐7 from diluted blood (500 ×) based on the focusing position shift phenomena,^[^ ^66^ ^]^ but it may be difficult to separate other bioparticles or even other CTCs, such as A549. As a result, researchers have to invest considerable time and money to customizing various novel technologies to cope with complex real‐world situations. In addition, this problem also causes significant inconvenience to users due to the lack of highly standardized guidelines, thus users have to rely on rich experience to choose suitable techniques.

### 4.2 Technological Advances and Perspectives

Despite the above challenges and limitations of existing isolation methods, the rapid advancement of emerging technologies, such as 3D printing and artificial intelligence (AI), offer promising possibilities for addressing these issues. The following sections discuss potential solutions and future prospects for each of these challenges.

#### 4.2.1 Emerging 3D Printing Technologies

3D printing is receiving traction as a compelling technique for fabricating microfluidic chips due to its unique advantages, such as rapid prototyping, optimization iteration, automated alignment, incorporating multiple types of materials, and integrating non‐planar microstructures.^[^ ^346^, ^347^ ^]^ 3D models are designed by computer‐aided design software and printed through a layer‐by‐layer process,^[^ ^348^ ^]^ enabling the flexible adjustment to complex models based on empirical data to cater to the specific demands of personalized microfluidic chips. In addition, numerous cutting‐edge engineering applications require the integration of various sensor modules, control units, and functional substrates.^[^ ^346^, ^349^, ^350^ ^]^ 3D printing offers opportunities for innovation in unconventional scenarios, owing to its versatile adaptability to diverse control algorithms and materials, as well as its capability to achieve high‐precision alignment.^[^ ^351^ ^]^ Although the advanced 3D printing technique is a promising avenue for manufacturing ultra‐precision micro/nano structures in microfluidic chips, several technical challenges remain in adapting to the rapidly evolving landscape of emerging technologies. For example, the resolution of 3D printing needs to be further improved to match the accuracy of feature structures, such as nano‐DLD pillar arrays.^[^ ^69^ ^]^ In addition, advanced computer vision technologies need to be developed to significantly reduce the alignment error of each layer.^[^ ^346^, ^352^ ^]^

#### 4.2.2 AI: Data‐Driven Design

Traditional design methods, driven by experiment, theory, and simulation, are usually time‐consuming and labor‐intensive, and typically result in suboptimal outcomes. Benefitting from the parallel advancements in computing hardware and algorithms,^[^ ^353^ ^]^ AI provides more opportunities for diversified design and rapid iteration of microfluidic chips. The experience of experts can be captured by AI, where large and heterogeneous data sets are trained and subsequently used for predicting future behaviors. Therefore, elegantly integrating AI into microfluidic chip design can bridge the knowledge gap between end users and experts, realizing the personalized design for specific application requirements. For isolation techniques, the design, use, and experimental results of microfluidic isolation chips can be quantitatively characterized by several parameters, such as structural size, flow rate, particle diameter, position difference, throughput, purity, and recovery rate.^[^ ^22^, ^28^, ^222^ ^]^ Thus, large data is generated in the process from design to experiment, as researchers usually need to design multi‐version microfluidic devices and conduct multi‐group experiments to investigate the main influencing factors and evolution rules. Precisely because of this, it is difficult to deeply reveal the isolation mechanism from first principle in most cases. AI models, trained on large‐scale data sets, can still provide predictive insights, even under complex flow conditions and in high‐dimensional design spaces.^[^ ^353^ ^]^ Thus, AI holds significant promise as a technique capable of addressing the challenges in the design of microfluidic isolation chips.

#### 4.2.3 Tunable Microfluidics

Most existing microfluidic chips perform well only within an extremely limited range of bioparticle size.^[^ ^345^ ^]^ Therefore, time‐consuming, expensive, and repetitive procedures, including design, manufacture, experiment, and optimization, are often required to meet the needs of different application scenarios. However, two emerging techniques hold promise for overcoming the challenge of technological flexibility: the integration of active manipulation elements and the adoption of flexible structures.^[^ ^12^, ^148^, ^345^ ^]^ For example, embedding microelectrodes at the bottom of a microfluidic device can introduce additional electrophoretic force on particles, further adjusting the focusing patterns of different‐size particles by changing the voltage and frequency.^[^ ^354^ ^]^ Similarly, advanced acoustophoresis techniques offer tunable approaches for bioparticle isolation, as the cutoff size can be precisely modified by varying the input power.^[^ ^147^ ^]^ In addition, the motion of bioparticles within a microfluidic chip is significantly influenced by channel geometry parameters, thus critical parameters such as channel length and width can be flexibly adjusted by stretching the structure to enable multi‐mode manipulation.^[^ ^345^ ^]^ Consequently, developing diversified and tunable isolation technologies is crucial for advancing their application in clinical settings and other research fields.

## Conflict of Interest

The authors declare no conflict of interest.

## References

[1] M. Wu , P. H. Huang , R. Zhang , Z. Mao , C. Chen , G. Kemeny , P. Li , A. V. Lee , R. Gyanchandani , A. J. Armstrong , M. Dao , S. Suresh , T. J. Huang , Small 2018, 14, 1801131. 10.1002/smll.201801131 PMC6105522 29968402

[2] A. Abdulla , W. Liu , A. Gholamipour‐Shirazi , J. Sun , X. Ding , Anal. Chem. 2018, 90, 4397. 29537252 10.1021/acs.analchem.7b04210

[3] M. Wu , Y. Ouyang , Z. Wang , R. Zhang , P.‐H. Huang , C. Chen , H. Li , P. Li , D. Quinn , M. Dao , S. Suresh , Y. Sadovsky , T. J. Huang , Proc. Natl. Acad. Sci. USA 2017, 114, 10584. 28923936 10.1073/pnas.1709210114 PMC5635903

[4] C. Théry , L. Zitvogel , S. Amigorena , Nat. Rev. Immunol. 2002, 2, 569. 12154376 10.1038/nri855

[5] Z. Li , C. Liu , Y. Cheng , Y. Li , J. Deng , L. Bai , L. Qin , H. Mei , M. Zeng , F. Tian , S. Zhang , J. Sun , Sci. Adv. 2023, 9, ade2819. 10.1126/sciadv.ade2819 PMC10121168 37083528

[6] S. Li , F. Ma , H. Bachman , C. E. Cameron , X. Zeng , T. J. Huang , J. Micromech. Microeng. 2016, 27, 015031. 28798539 10.1088/1361-6439/27/1/015031 PMC5546156

[7] F. Mayer , B. Haslinger , A. Shpylovyi , A. Weber , U. Windberger , B. Albrecht , R. Hahn , M. Cserjan‐Puschmann , G. Striedner , J. Chem. Technol. Biotechnol. 2023, 98, 1443.

[8] K. G. Phillips , C. R. Velasco , J. Li , A. Kolatkar , M. Luttgen , K. Bethel , B. Duggan , P. Kuhn , O. McCarty , Front. Oncol 2012, 2, 72. 22826822 10.3389/fonc.2012.00072 PMC3399133

[9] E. Ozkumur , A. M. Shah , J. C. Ciciliano , B. L. Emmink , D. T. Miyamoto , E. Brachtel , M. Yu , P.‐i. Chen , B. Morgan , J. Trautwein , A. Kimura , S. Sengupta , S. L. Stott , N. M. Karabacak , T. A. Barber , J. R. Walsh , K. Smith , P. S. Spuhler , J. P. Sullivan , R. J. Lee , D. T. Ting , X. Luo , A. T. Shaw , A. Bardia , L. V. Sequist , D. N. Louis , S. Maheswaran , R. Kapur , D. A. Haber , M. Toner , Sci. Transl. Med. 2013, 5, 179ra47. 10.1126/scitranslmed.3005616 PMC3760275 23552373

[10] P. Noris , G. Biino , A. Pecci , E. Civaschi , A. Savoia , M. Seri , F. Melazzini , G. Loffredo , G. Russo , V. Bozzi , L. D. Notarangelo , P. Gresele , P. G. Heller , N. Pujol‐Moix , S. Kunishima , M. Cattaneo , J. Bussel , E. De Candia , C. Cagioni , U. Ramenghi , S. Barozzi , F. Fabris , C. L. Balduini , Blood 2014, 124, 4. 24990887 10.1182/blood-2014-03-564328 PMC4126341

[11] J. N. Thon , J. E. Italiano , Blood 2012, 120, 1552. 22665937 10.1182/blood-2012-04-408724 PMC3429301

[12] J. Xia , Z. Wang , R. Becker , F. Li , F. Wei , S. Yang , J. Rich , K. Li , J. Rufo , J. Qian , K. Yang , C. Chen , Y. Gu , R. Zhong , P. J. Lee , D. T. W. Wong , L. P. Lee , T. J. Huang , ACS Nano 2024, 18, 22596. 39132820 10.1021/acsnano.4c09692 PMC12212744

[13] S. Fahimirad , Z. Fahimirad , M. Sillanpää , Sci. Total Environ. 2021, 751, 141673. 32866832 10.1016/j.scitotenv.2020.141673 PMC7428676

[14] M. Poudineh , E. H. Sargent , K. Pantel , S. O. Kelley , Nat. Biomed. Eng 2018, 2, 72. 31015625 10.1038/s41551-018-0190-5

[15] A. Ring , B. D. Nguyen‐Sträul , A. Wicki , N. Aceto , Nat. Rev. Cancer 2022, 23, 95. 36494603 10.1038/s41568-022-00536-4 PMC9734934

[16] R. Lawrence , M. Watters , C. R. Davies , K. Pantel , Y.‐J. Lu , Nat. Rev. Clin. Oncol. 2023, 20, 487. 37268719 10.1038/s41571-023-00781-y PMC10237083

[17] S. H. Lee , B. Cha , H. G. Yi , J. Kim , J. S. Jeon , J. Park , Sens. Actuator B‐Chem. 2024, 417, 136161.

[18] V. Laxmi , S. Tripathi , S. S. Joshi , A. Agrawal , Ind. Eng. Chem. Res. 2020, 59, 4792.

[19] C. Wang , J. Qiu , M. Liu , Y. Wang , Y. Yu , H. Liu , Y. Zhang , L. Han , Adv. Sci. 2024, 11, 2401263. 10.1002/advs.202401263 PMC11267386 38767182

[20] M. M. Usaj , C. H. L. Yeung , H. Friesen , C. Boone , B. J. Andrews , Cell Syst 2021, 12, 608. 34139168 10.1016/j.cels.2021.05.010 PMC9112900

[21] P. Paterlini‐Brechot , N. L. Benali , Cancer Lett 2007, 253, 180. 17314005 10.1016/j.canlet.2006.12.014

[22] Y. Cheng , S. Zhang , L. Qin , J. Zhao , H. Song , Y. Yuan , J. Sun , F. Tian , C. Liu , Anal. Chem. 2023, 95, 3468. 36725367 10.1021/acs.analchem.2c05257

[23] C. Liu , J. Zhao , F. Tian , J. Chang , W. Zhang , J. Sun , J. Am. Chem. Soc. 2019, 141, 3817. 30789261 10.1021/jacs.9b00007

[24] Y. Yang , W. Pang , H. Zhang , W. Cui , K. Jin , C. Sun , Y. Wang , L. Zhang , X. Ren , X. Duan , Microsyst. Nanoeng. 2022, 8, 88. 35935274 10.1038/s41378-022-00424-9 PMC9352906

[25] M. Bayareh , Chem. Eng. Process. 2020, 153, 107984.

[26] L. Menze , P. A. Duarte , L. Haddon , M. Chu , J. Chen , ACS Nano 2021, 16, 211. 34559518 10.1021/acsnano.1c05668

[27] A. Mishra , T. D. Dubash , J. F. Edd , M. K. Jewett , S. G. Garre , N. M. Karabacak , D. C. Rabe , B. R. Mutlu , J. R. Walsh , R. Kapur , S. L. Stott , S. Maheswaran , D. A. Haber , M. Toner , Proc. Natl. Acad. Sci. USA 2020, 117, 16839. 32641515 10.1073/pnas.2006388117 PMC7382214

[28] C. Ni , Y. Chen , Y. Zhou , D. Jiang , Z. Ni , N. Xiang , Sens. Actuator B‐Chem. 2023, 397, 134619.

[29] H. Tang , J. Niu , H. Jin , S. Lin , D. Cui , Microsyst. Nanoeng. 2022, 8, 62. 35685963 10.1038/s41378-022-00386-y PMC9170746

[30] M. Ali , S. H. Lee , B. Cha , W. Kim , N.‐E. Oyunbaatar , D.‐W. Lee , J. Park , Sens. Actuator B‐Chem. 2024, 415, 135988.

[31] M. Li , D. Li , Y. Song , D. Li , J. Colloid Interface Sci. 2022, 612, 23. 34974255 10.1016/j.jcis.2021.12.140

[32] Y. Liu , R. M. S. Vieira , L. Mao , ACS Nano 2022, 17, 94. 36541668 10.1021/acsnano.2c04542

[33] Z. R. Gagnon , Electrophoresis 2011, 32, 2466. 21922493 10.1002/elps.201100060

[34] J. Nam , H. Huang , H. Lim , C. Lim , S. Shin , Anal. Chem. 2013, 85, 7316. 23815099 10.1021/ac4012057

[35] B. Chen , J. J. Zheng , K. F. Gao , X. J. Hu , S. S. Guo , X. Z. Zhao , F. Liao , Y. Yang , W. Liu , ACS Appl. Bio Mater. 2022, 5, 2768. 10.1021/acsabm.2c00204 35537085

[36] W. Al‐Faqheri , T. H. G. Thio , M. A. Qasaimeh , A. Dietzel , M. Madou , A. a. Al‐Halhouli , Microfluid. Nanofluid. 2017, 21, 102.

[37] M. Bagi , F. Amjad , S. M. Ghoreishian , S. Sohrabi Shahsavari , Y. S. Huh , M. K. Moraveji , S. Shimpalee , BioChip J. 2024, 18, 45.

[38] N. Lu , H. M. Tay , C. Petchakup , L. He , L. Gong , K. K. Maw , S. Y. Leong , W. W. Lok , H. B. Ong , R. Guo , K. H. H. Li , H. W. Hou , Lab Chip 2023, 23, 1226. 36655549 10.1039/d2lc00904h

[39] Y. Jia , L. Yu , T. Ma , W. Xu , H. Qian , Y. Sun , H. Shi , Theranostics 2022, 12, 6548. 36185597 10.7150/thno.74305 PMC9516236

[40] J. Zhou , I. Papautsky , Microsyst. Nanoeng. 2020, 6, 113. 34567720 10.1038/s41378-020-00218-x PMC8433399

[41] J. Zhang , S. Yan , D. Yuan , G. Alici , N. T. Nguyen , M. E Warkiani , W. Li , Lab Chip 2016, 16, 10. 26584257 10.1039/c5lc01159k

[42] A. Dalili , E. Samiei , M. Hoorfar , Analyst 2018, 144, 87. 30402633 10.1039/c8an01061g

[43] W. Tang , S. Zhu , D. Jiang , L. Zhu , J. Yang , N. Xiang , Lab Chip 2020, 20, 3485. 32910129 10.1039/d0lc00714e

[44] N. Xiang , Z. Ni , Lab Chip 2022, 22, 4792. 36263793 10.1039/d2lc00722c

[45] T. Salafi , Y. Zhang , Y. Zhang , Nano‐Micro Lett. 2019, 11, 77. 10.1007/s40820-019-0308-7 PMC7770818 34138050

[46] G. de Timary , C. J. Rousseau , L. Van Melderen , B. Scheid , Lab Chip 2023, 23, 659. 36562423 10.1039/d2lc00969b

[47] G. Li , Y. Ji , Y. Wu , Y. Liu , H. Li , Y. Wang , M. Chi , H. Sun , H. Zhu , Biosens. Bioelectron. 2023, 237, 115451. 37327603 10.1016/j.bios.2023.115451

[48] H. Gao , J. Zhou , M. M. Naderi , Z. Peng , I. Papautsky , Microsyst. Nanoeng. 2023, 9, 73. 37288322 10.1038/s41378-023-00520-4 PMC10241945

[49] D. D. Carlo , D. Irimia , R. G. Tompkins , M. Toner , Proc. Natl. Acad. Sci. USA 2007, 104, 18892. 18025477 10.1073/pnas.0704958104 PMC2141878

[50] D. D. Carlo , J. F. Edd , K. J. Humphry , H. A. Stone , M. Toner , Phys. Rev. Lett. 2009, 102, 094503. 19392526 10.1103/PhysRevLett.102.094503 PMC4485430

[51] C. Xuan , W. Liang , B. He , B. Wen , Phys. Rev. Fluids 2022, 7, 064201.

[52] Z. Chen , L. Zhao , L. Wei , Z. Huang , P. Yin , X. Huang , H. Shi , B. Hu , J. Tian , Sens. Actuator B‐Chem. 2019, 301, 127125.

[53] Y. Meng , Y. Zhang , M. Bühler , S. Wang , M. Asghari , A. Stürchler , B. Mateescu , T. Weiss , S. Stavrakis , A. J. deMello , Sci. Adv. 2023, 9, adi5296. 10.1126/sciadv.adi5296 PMC10558121 37801500

[54] M. A. Faridi , H. Ramachandraiah , I. Banerjee , S. Ardabili , S. Zelenin , A. Russom , J. Nanobiotechnol. 2017, 15, 3. 10.1186/s12951-016-0235-4 PMC5210221 28052769

[55] J. Zhou , A. Kulasinghe , A. Bogseth , K. O'Byrne , C. Punyadeera , I. Papautsky , Microsyst. Nanoeng. 2019, 5, 8. 31057935 10.1038/s41378-019-0045-6 PMC6387977

[56] A. Abdulla , T. Zhang , S. Li , W. Guo , A. R. Warden , Y. Xin , N. Maboyi , J. Lou , H. Xie , X. Ding , Microsyst. Nanoeng. 2022, 8, 13. 35136652 10.1038/s41378-021-00342-2 PMC8807661

[57] Y. Shen , Y. Yalikun , Y. Tanaka , Sens. Actuator B‐Chem. 2019, 282, 268.

[58] K. Loutherback , J. D'Silva , L. Liu , A. Wu , R. H. Austin , J. C. Sturm , AIP Adv. 2012, 2, 042107. 23112922 10.1063/1.4758131 PMC3477176

[59] M. Charjouei Moghadam , A. Eilaghi , P. Rezai , Microfluid. Nanofluid 2019, 23, 135.

[60] N. Xiang , J. Wang , Q. Li , Y. Han , D. Huang , Z. Ni , Anal. Chem. 2019, 91, 10328. 31304740 10.1021/acs.analchem.9b02863

[61] L. Ren , S. Yang , P. Zhang , Z. Qu , Z. Mao , P. H. Huang , Y. Chen , M. Wu , L. Wang , P. Li , T. J. Huang , Small 2018, 14, 1801996. 10.1002/smll.201801996 PMC6291339 30168662

[62] F. Javi , M. Zaferani , N. Lopez‐Barbosa , M. P. DeLisa , A. Abbaspourrad , Microfluid. Nanofluid. 2022, 26, 54.

[63] Z. Zhao , S. Yang , L. Feng , L. Zhang , J. Wang , K. Chang , M. Chen , Adv. Mater. Technol. 2023, 8, 2202201.

[64] Y. Xie , J. Rufo , R. Zhong , J. Rich , P. Li , K. W. Leong , T. J. Huang , ACS Nano 2020, 14, 16220. 33252215 10.1021/acsnano.0c06336 PMC8164652

[65] S. Yan , J. Zhang , D. Yuan , W. Li , Electrophoresis 2016, 38, 238. 27718260 10.1002/elps.201600386

[66] J.‐a. Kim , J.‐R. Lee , T.‐J. Je , E.‐c. Jeon , W. Lee , Anal. Chem. 2018, 90, 1827. 29271639 10.1021/acs.analchem.7b03851

[67] V. Laxmi , S. Tripathi , S. S. Joshi , A. Agrawal , J. Indian Inst. Sci. 2018, 98, 185.

[68] J. Cruz , T. Graells , M. Walldén , K. Hjort , Lab Chip 2019, 19, 1257. 30821308 10.1039/c9lc00080a

[69] B. H. Wunsch , J. T. Smith , S. M. Gifford , C. Wang , M. Brink , R. L. Bruce , R. H. Austin , G. Stolovitzky , Y. Astier , Nat. Nanotechnol. 2016, 11, 936. 27479757 10.1038/nnano.2016.134

[70] S. Lin , X. Zhi , D. Chen , F. Xia , Y. Shen , J. Niu , S. Huang , J. Song , J. Miao , D. Cui , X. Ding , Biosens. Bioelectron. 2019, 129, 175. 30710755 10.1016/j.bios.2018.12.058

[71] X. Peng , G. Luo , Z. Wu , W. Wen , X. Zhang , S. Wang , ACS Appl. Mater. Interfaces 2019, 11, 41148. 31613583 10.1021/acsami.9b16718

[72] M. Antfolk , C. Magnusson , P. Augustsson , H. Lilja , T. Laurell , Anal. Chem. 2015, 87, 9322. 26309066 10.1021/acs.analchem.5b02023

[73] H. Jeon , S. H. Lee , J. Shin , K. Song , N. Ahn , J. Park , Microsyst. Nanoeng. 2024, 10, 15. 38264707 10.1038/s41378-023-00633-w PMC10803301

[74] P. Li , M. Kaslan , S. H. Lee , J. Yao , Z. Gao , Theranostics 2017, 7, 789. 28255367 10.7150/thno.18133 PMC5327650

[75] D. Yuan , Q. Zhao , S. Yan , S. Y. Tang , G. Alici , J. Zhang , W. Li , Lab Chip 2018, 18, 551. 29340388 10.1039/c7lc01076a

[76] M. Hejazian , W. Li , N.‐T. Nguyen , Lab Chip 2015, 15, 959. 25537573 10.1039/c4lc01422g

[77] T. Zhu , R. Cheng , Y. Liu , J. He , L. Mao , Microfluid. Nanofluid 2014, 17, 973.

[78] M. Tang , J. Feng , H.‐F. Xia , C.‐M. Xu , L.‐L. Wu , M. Wu , S.‐L. Hong , G. Chen , Z.‐L. Zhang , Chem. Commun. 2023, 59, 11955. 10.1039/d3cc04062c 37727113

[79] F. Alnaimat , S. Dagher , B. Mathew , A. Hilal‐Alnqbi , S. Khashan , Chem. Rec. 2018, 18, 1596. 29888856 10.1002/tcr.201800018

[80] A. Seyfoori , S. A. Seyyed Ebrahimi , M. Samandari , E. Samiei , E. Stefanek , C. Garnis , M. Akbari , Small 2023, 19, 2205320. 10.1002/smll.202205320 36720798

[81] A. Tay , D. Pfeiffer , K. Rowe , A. Tannenbaum , F. Popp , R. Strangeway , D. Schüler , D. Di Carlo , R. M. Kelly , Appl. Environ. Microbiol. 2018, 84, 01308. 10.1128/AEM.01308-18 PMC6102981 29959254

[82] E. P. Furlani , J. Phys. D‐Appl. Phys. 2007, 40, 1313.

[83] L. Luo , Y. He , Cancer Med. 2020, 9, 4207. 32325536 10.1002/cam4.3077 PMC7300401

[84] Q. Chen , D. Li , A. Malekanfard , Q. Cao , J. Lin , M. Wang , X. Han , X. Xuan , Anal. Chem. 2018, 90, 8600. 29923401 10.1021/acs.analchem.8b01813

[85] X. Chen , H. Ding , D. Zhang , K. Zhao , J. Gao , B. Lin , C. Huang , Y. Song , G. Zhao , Y. Ma , L. Wu , C. Yang , Adv. Sci. 2021, 8, 2102070. 10.1002/advs.202102070 PMC8529431 34473422

[86] K. Xiong , W. Wei , Y. Jin , S. Wang , D. Zhao , S. Wang , X. Gao , C. Qiao , H. Yue , G. Ma , H. Y. Xie , Adv. Mater. 2016, 28, 7929. 27376951 10.1002/adma.201601643

[87] Z. Wang , H. Wang , S. Lin , S. Ahmed , S. Angers , E. H. Sargent , S. O. Kelley , Nano Lett. 2022, 22, 4774. 35639489 10.1021/acs.nanolett.2c01018

[88] Z. Yu , S. Lin , F. Xia , Y. Liu , D. Zhang , F. Wang , Y. Wang , Q. Li , J. Niu , C. Cao , D. Cui , N. Sheng , J. Ren , Z. Wang , D. Chen , Biosens. Bioelectron. 2021, 194, 113594. 34474280 10.1016/j.bios.2021.113594

[89] L. Xue , L. Zheng , H. Zhang , X. Jin , J. Lin , Sens. Actuator B‐Chem. 2018, 265, 318.

[90] A. Munaz , M. J. A. Shiddiky , N.‐T. Nguyen , Sens. Actuator B‐Chem. 2018, 275, 459.

[91] W. Zhao , R. Cheng , J. R. Miller , L. Mao , Adv. Funct. Mater. 2016, 26, 3916. 28663720 10.1002/adfm.201504178 PMC5487005

[92] A. Shamloo , M. Besanjideh , IEEE Trans. Biomed. Eng. 2020, 67, 372. 31034404 10.1109/TBME.2019.2913670

[93] Z. Chen , B. Chen , M. He , B. Hu , Anal. Chem. 2022, 94, 6649. 35481740 10.1021/acs.analchem.1c04216

[94] T. Zhu , D. J. Lichlyter , M. A. Haidekker , L. Mao , Microfluid. Nanofluid. 2011, 10, 1233.

[95] W. H. Chong , S. S. Leong , J. Lim , Electrophoresis 2021, 42, 2303. 34213767 10.1002/elps.202100081

[96] A. R. Kosea , B. Fischerb , L. Maoc , H. Kosera , Proc. Natl. Acad. Sci. USA 2009, 106, 21478.19995975

[97] W. Zhao , T. Zhu , R. Cheng , Y. Liu , J. He , H. Qiu , L. Wang , T. Nagy , T. D. Querec , E. R. Unger , L. Mao , Adv. Funct. Mater. 2015, 26, 3990. 27478429 10.1002/adfm.201503838 PMC4963013

[98] T. Zhu , R. Cheng , S. A. Lee , E. Rajaraman , M. A. Eiteman , T. D. Querec , E. R. Unger , L. Mao , Microfluid. Nanofluid 2012, 13, 645. 26430394 10.1007/s10404-012-1004-9 PMC4587988

[99] S. D. Conner , S. L. Schmid , Nature 2003, 422, 37. 12621426 10.1038/nature01451

[100] W. Zhao , R. Cheng , S. H. Lim , J. R. Miller , W. Zhang , W. Tang , J. Xie , L. Mao , Lab Chip 2017, 17, 2243. 28590489 10.1039/c7lc00327g PMC5543773

[101] D. Robert , N. Pamme , H. Conjeaud , F. Gazeau , A. Iles , C. Wilhelm , Lab Chip 2011, 11, 1902. 21512692 10.1039/c0lc00656d

[102] A. Kefayat , O. Sartipzadeh , F. Molaabasi , M. Amiri , R. Gholami , M. Mirzadeh , F. Shokati , M. Khandaei , F. Ghahremani , S. A. Poursamar , R. Sarrami‐Forooshani , Anal. Chem. 2024, 96, 4377. 38442207 10.1021/acs.analchem.3c03567

[103] M. Tang , H.‐F. Xia , C.‐M. Xu , J. Feng , J.‐G. Ren , F. Miao , M. Wu , L.‐L. Wu , D.‐W. Pang , G. Chen , Z.‐L. Zhang , Anal. Chem. 2019, 91, 15260. 31692331 10.1021/acs.analchem.9b04286

[104] J. Zhang , X. Jian , S. Bai , G. Xu , M. Du , C. Guo , Y. Guan , J. Nanopart. Res. 2023, 25, 217.

[105] Q. Chen , D. Li , J. Lin , M. Wang , X. Xuan , Anal. Chem. 2017, 89, 6915. 28548482 10.1021/acs.analchem.7b01608

[106] A. Al‐Ali , W. Waheed , E. Abu‐Nada , A. Alazzam , J. Chromatogr. A 2022, 1676, 463268. 35779391 10.1016/j.chroma.2022.463268

[107] E. O. Adekanmbi , S. K. Srivastava , Lab Chip 2016, 16, 2148. 27191245 10.1039/c6lc00355a

[108] E. A. Kwizera , M. Sun , A. M. White , J. Li , X. He , ACS Biomater. Sci. Eng. 2021, 7, 2043. 33871975 10.1021/acsbiomaterials.1c00083 PMC8205986

[109] L. A. N. Julius , D. Akgül , G. Krishnan , F. Falk , J. Korvink , V. Badilita , Microsyst. Nanoeng. 2024, 10, 29. 38434587 10.1038/s41378-024-00654-z PMC10907756

[110] M. Wu , Y. Gao , Q. Luan , I. Papautsky , X. Chen , J. Xu , Electrophoresis 2023, 44, 1802. 37026613 10.1002/elps.202200287

[111] T. N. G. Adams , A. Y. L. Jiang , P. D. Vyas , L. A. Flanagan , Methods 2018, 133, 91. 28864355 10.1016/j.ymeth.2017.08.016 PMC6058702

[112] J. Niu , S. Lin , Y. Xu , S. Tong , Z. Wang , S. Cui , Y. Liu , D. Chen , D. Cui , Talanta 2024, 279, 126585. 39053361 10.1016/j.talanta.2024.126585

[113] Z. Tian , X. Wang , J. Chen , Microsyst. Nanoeng. 2024, 10, 117. 39187499 10.1038/s41378-024-00750-0 PMC11347631

[114] V. Shirmohammadli , N. Manavizadeh , IEEE Trans. Electron Devices 2019, 66, 4075.

[115] Y.‐C. Kung , K. R. Niazi , P.‐Y. Chiou , Lab Chip 2021, 21, 1049. 33313615 10.1039/d0lc00853b

[116] S. D. Ibsen , J. Wright , J. M. Lewis , S. Kim , S.‐Y. Ko , J. Ong , S. Manouchehri , A. Vyas , J. Akers , C. C. Chen , B. S. Carter , S. C. Esener , M. J. Heller , ACS Nano 2017, 11, 6641. 28671449 10.1021/acsnano.7b00549

[117] J. L. Duncan , R. V. Davalos , Electrophoresis 2021, 42, 2423. 34609740 10.1002/elps.202100135

[118] F. Salimian Rizi , S. Talebi , M. K. D. Manshadi , M. Mohammadi , Biomech. Model. Mechanobiol. 2023, 22, 825. 36787033 10.1007/s10237-022-01683-1

[119] S. Ayala‐Mar , V. H. Perez‐Gonzalez , M. A. Mata‐Gómez , R. C. Gallo‐Villanueva , J. González‐Valdez , Anal. Chem. 2019, 91, 14975. 31738514 10.1021/acs.analchem.9b03448

[120] H.‐W. Su , J. L. Prieto , J. Voldman , Lab Chip 2013, 13, 4109. 23970334 10.1039/c3lc50392e

[121] M. D. Vahey , J. Voldman , Anal. Chem. 2009, 81, 2446. 19253950 10.1021/ac8019575 PMC2675787

[122] X. Zhu , K.‐W. Tung , P.‐Y. Chiou , Appl. Phys. Lett. 2017, 111, 143506.

[123] Y. C. Kung , K. W. Huang , W. Chong , P. Y. Chiou , Small 2016, 12, 4302. 10.1002/smll.201600996 27348575

[124] N. Mittal , A. Rosenthal , J. Voldman , Lab Chip 2007, 7, 1146. 17713613 10.1039/b706342c

[125] R. Martinez‐Duarte , Electrophoresis 2012, 33, 3110. 22941778 10.1002/elps.201200242

[126] A. Gencoglu , A. Minerick , Lab Chip 2009, 9, 1866. 19532961 10.1039/b820126a

[127] J. Suehiro , Z. Guangbin , M. Imamura , M. Hara , IEEE Trans. Ind. Appl. 2003, 39, 1514.

[128] C. Jun , C. Ping , H. FangJun , Sci. China Ser. E‐Technol. Sci. 2009, 52, 3477.

[129] S. Yang , J. Rufo , R. Zhong , J. Rich , Z. Wang , L. P. Lee , T. J. Huang , Nat. Protoc. 2023, 18, 2441. 37468650 10.1038/s41596-023-00844-5 PMC11052649

[130] L. Shen , Z. Tian , K. Yang , J. Rich , J. Zhang , J. Xia , W. Collyer , B. Lu , N. Hao , Z. Pei , C. Chen , T. J. Huang , Sci. Adv. 2024, 10, ado8992. 10.1126/sciadv.ado8992 PMC11305384 39110808

[131] K. Dholakia , B. W. Drinkwater , M. Ritsch‐Marte , Nat. Rev. Phys. 2020, 2, 480.

[132] X. Wang , L. Wang , H. Feng , J. Jin , C. Zhao , Sens. Actuator A‐Phys 2023, 362, 114656.

[133] Z. Wu , H. Jiang , L. Zhang , K. Yi , H. Cui , F. Wang , W. Liu , X. Zhao , F. Zhou , S. Guo , Lab Chip 2019, 19, 3922. 31693035 10.1039/c9lc00874h

[134] A. Lenshof , M. Evander , T. Laurell , J. Nilsson , Lab Chip 2012, 12, 684. 22246532 10.1039/c1lc20996e

[135] Z. Ma , J. Xia , N. Upreti , E. David , J. Rufo , Y. Gu , K. Yang , S. Yang , X. Xu , J. Kwun , E. Chambers , T. J. Huang , Microsyst. Nanoeng. 2024, 10, 83. 38915828 10.1038/s41378-024-00707-3 PMC11194281

[136] Y. Fan , X. Wang , J. Ren , F. Lin , J. Wu , Microsyst. Nanoeng. 2022, 8, 1. 36060525 10.1038/s41378-022-00435-6 PMC9434534

[137] C. Magnusson , P. Augustsson , E. Undvall Anand , A. Lenshof , A. Josefsson , K. Welén , A. Bjartell , Y. Ceder , H. Lilja , T. Laurell , Anal. Chem. 2024, 96, 6914. 38655666 10.1021/acs.analchem.3c05371 PMC11079855

[138] L. Li , C. W. Shields , J. Huang , Y. Zhang , K. A. Ohiri , B. B. Yellen , A. Chilkoti , G. P. López , Analyst 2020, 145, 8087. 10.1039/d0an01164a 33079081

[139] S. Xue , Q. Xu , Z. Xu , X. Zhang , H. Zhang , X. Zhang , F. He , Y. Chen , Y. Xue , P. Hao , Anal. Chem. 2023, 95, 4282. 36815437 10.1021/acs.analchem.2c03841

[140] G. Liu , W. Shen , Y. Li , H. Zhao , X. Li , C. Wang , F. He , Sens. Actuator A‐Phys 2022, 341, 113589.

[141] M. Wu , A. Ozcelik , J. Rufo , Z. Wang , R. Fang , T. Jun Huang , Microsyst. Nanoeng. 2019, 5, 32. 31231539 10.1038/s41378-019-0064-3 PMC6545324

[142] F. Wu , M. H. Shen , J. Yang , H. Wang , R. Mikhaylov , A. Clayton , X. Qin , C. Sun , Z. Xie , M. Cai , J. Wei , D. Liang , F. Yuan , Z. Wu , Y. Fu , Z. Yang , X. Sun , L. Tian , X. Yang , IEEE Electron Device Lett. 2021, 42, 577.

[143] D. J. Collins , A. Neild , Y. Ai , Lab Chip 2016, 16, 471. 26646200 10.1039/c5lc01335f

[144] Z. Ma , D. J. Collins , J. Guo , Y. Ai , Anal. Chem. 2016, 88, 11844. 27934119 10.1021/acs.analchem.6b03580

[145] M. C. Jo , R. Guldiken , Sens. Actuator A‐Phys 2012, 187, 22.

[146] K. Wang , W. Zhou , Z. Lin , F. Cai , F. Li , J. Wu , L. Meng , L. Niu , H. Zheng , Sens. Actuator B‐Chem. 2018, 258, 1174.

[147] J. Zhang , C. Chen , R. Becker , J. Rufo , S. Yang , J. Mai , P. Zhang , Y. Gu , Z. Wang , Z. Ma , J. Xia , N. Hao , Z. Tian , D. T. W. Wong , Y. Sadovsky , L. P. Lee , T. J. Huang , Sci. Adv. 2022, 8, ade0640. 10.1126/sciadv.ade0640 PMC9683722 36417505

[148] Y. Yang , L. Zhang , K. Jin , M. He , W. Wei , X. Chen , Q. Yang , Y. Wang , W. Pang , X. Ren , X. Duan , Sci. Adv. 2022, 8, abn8440. 10.1126/sciadv.abn8440 PMC9337757 35905179

[149] Y. Gu , C. Chen , Z. Mao , H. Bachman , R. Becker , J. Rufo , Z. Wang , P. Zhang , J. Mai , S. Yang , J. Zhang , S. Zhao , Y. Ouyang , D. T. W. Wong , Y. Sadovsky , T. J. Huang , Sci. Adv. 2021, 7, abc0467. 10.1126/sciadv.abc0467 PMC7775782 33523836

[150] T. D. Naquin , A. J. Canning , Y. Gu , J. Chen , C. M. Naquin , J. Xia , B. Lu , S. Yang , A. Koroza , K. Lin , H.‐N. Wang , W. R. Jeck , L. P. Lee , T. Vo‐Dinh , T. J. Huang , Sci. Adv. 2024, 10, adm8597. 10.1126/sciadv.adm8597 PMC10923504 38457504

[151] W. Su , H. Li , W. Chen , J. Qin , Trac‐Trends Anal. Chem. 2019, 118, 686.

[152] H.‐K. Woo , V. Sunkara , J. Park , T.‐H. Kim , J.‐R. Han , C.‐J. Kim , H.‐I. Choi , Y.‐K. Kim , Y.‐K. Cho , ACS Nano 2017, 11, 1360. 28068467 10.1021/acsnano.6b06131

[153] L. R. Huang , E. C. Cox , R. H. Austin , J. C. Sturm , Science 2004, 304, 987. 15143275 10.1126/science.1094567

[154] S. Zhao , M. Wu , S. Yang , Y. Wu , Y. Gu , C. Chen , J. Ye , Z. Xie , Z. Tian , H. Bachman , P.‐H. Huang , J. Xia , P. Zhang , H. Zhang , T. J. Huang , Lab Chip 2020, 20, 1298. 32195522 10.1039/d0lc00106f PMC7199844

[155] J. L. Han , H. Hu , Q. Y. Huang , Y. L. Lei , Sens. Actuator A‐Phys. 2021, 326, 112731.

[156] J. Rufo , F. Cai , J. Friend , M. Wiklund , T. J. Huang , Nat. Rev. Method. Prim. 2022, 2, 30.

[157] A. A. Nawaz , D. Soteriou , C. K. Xu , R. Goswami , M. Herbig , J. Guck , S. Girardo , Lab Chip 2023, 23, 372. 36620943 10.1039/d2lc00636g PMC9844123

[158] P. Zhang , H. Bachman , A. Ozcelik , T. J. Huang , Annu. Rev. Anal. Chem. 2020, 13, 17. 10.1146/annurev-anchem-090919-102205 PMC7415005 32531185

[159] A. Ntimtsas , E. Gizeli , Sens. Actuator A‐Phys 2024, 378, 115814.

[160] Y. Ren , Q. Chen , M. He , X. Zhang , H. Qi , Y. Yan , ACS Nano 2021, 15, 6105. 33834771 10.1021/acsnano.1c00466

[161] S. B. Kim , S. Y. Yoon , H. J. Sung , S. S. Kim , Anal. Chem. 2008, 80, 2628. 18275223 10.1021/ac8000918

[162] W. Wu , X. Zhu , Y. Zuo , L. Liang , S. Zhang , X. Zhang , Y. Yang , ACS Photonics 2016, 3, 2497.

[163] A. Rohrbach , E. H. K. Stelzer , J. Opt. Soc. Am. A 2001, 18, 839. 10.1364/josaa.18.000839 11318334

[164] J. Li , Z. Chen , Y. Liu , P. S. Kollipara , Y. Feng , Z. Zhang , Y. Zheng , Sci. Adv. 2021, 7, 1101. 10.1126/sciadv.abh1101 PMC8232904 34172454

[165] G. F. Vasse , P. Buzón , B. N. Melgert , W. H. Roos , P. van Rijn , Small Methods 2021, 5, 2000849. 10.1002/smtd.202000849 34927846

[166] X. Xie , X. Wang , C. Min , H. Ma , Y. Yuan , Z. Zhou , Y. Zhang , J. Bu , X. Yuan , Photonics Res. 2021, 10, 166.

[167] S. B. Kim , S. S. Kim , J. Opt. Soc. Am. B 2006, 23, 897.

[168] S. B. Kim , J. H. Kim , S. S. Kim , Appl. Optics 2006, 45, 6919. 10.1364/ao.45.006919 16946766

[169] H. Zhao , L. K. Chin , Y. Shi , P. Y. Liu , Y. Zhang , H. Cai , E. P. H. Yap , W. Ser , A.‐Q. Liu , Sens. Actuator B‐Chem. 2021, 331, 129428.

[170] P. F. Barker , Phys. Rev. Lett. 2010, 105, 073002. 20868038 10.1103/PhysRevLett.105.073002

[171] P. Y. Liu , L. K. Chin , W. Ser , H. F. Chen , C. M. Hsieh , C. H. Lee , K. B. Sung , T. C. Ayi , P. H. Yap , B. Liedberg , K. Wang , T. Bourouina , Y. Leprince‐Wang , Lab Chip 2016, 16, 634. 26732872 10.1039/c5lc01445j

[172] W. K. Metcalf , N. F. Metcalf , R. N. Gould , Antibiotica et Chemotherapia 1978, 22, 149.

[173] X. Hu , D. Zhu , M. Chen , K. Chen , H. Liu , W. Liu , Y. Yang , Lab Chip 2019, 19, 2549. 31263813 10.1039/c9lc00361d

[174] Y. Park , M. Diez‐Silva , G. Popescu , G. Lykotrafitis , W. Choi , M. S. Feld , S. Suresh , Proc. Natl. Acad. Sci. USA 2008, 105, 13730. 18772382 10.1073/pnas.0806100105 PMC2529332

[175] P. Li , Z. Ma , Y. Zhou , D. J. Collins , Z. Wang , Y. Ai , Anal. Chem. 2019, 91, 9970. 31179691 10.1021/acs.analchem.9b01708

[176] D. Schraivogel , L. M. Steinmetz , Mol. Syst. Biol. 2023, 19, 11254. 10.15252/msb.202211254 PMC9996229 36779527

[177] H. Pereira , P. S. C. Schulze , L. M. Schüler , T. Santos , L. Barreira , J. Varela , Algal Res. 2018, 30, 113.

[178] G. Sun , L. Qu , F. Azi , Y. Liu , J. Li , X. Lv , G. Du , J. Chen , C.‐H. Chen , L. Liu , Biosens. Bioelectron. 2023, 225, 115107. 36731396 10.1016/j.bios.2023.115107

[179] C. Wang , Y. Ma , Z. Pei , F. Song , J. Zhong , Y. Wang , X. Yan , P. Dai , Y. Jiang , J. Qiu , M. Shi , X. Wu , Cytom. Part A 2021, 101, 311. 10.1002/cyto.a.24521 34806837

[180] Z. Ma , Y. Zhou , D. J. Collins , Y. Ai , Lab Chip 2017, 17, 3176. 28815231 10.1039/c7lc00678k

[181] C. Liu , G. Hu , X. Jiang , J. Sun , Lab Chip 2015, 15, 1168. 25563524 10.1039/c4lc01216j

[182] K. Lee , R. Mishra , T. Kim , Sens. Actuator A‐Phys. 2023, 363, 114688.

[183] R. Nasiri , A. Shamloo , S. Ahadian , L. Amirifar , J. Akbari , M. J. Goudie , K. Lee , N. Ashammakhi , M. R. Dokmeci , D. Di Carlo , A. Khademhosseini , Small 2020, 16, 2000171. 10.1002/smll.202000171 32529791

[184] H. Amini , W. Lee , D. Di Carlo , Lab Chip 2014, 14, 2739. 24914632 10.1039/c4lc00128a

[185] R. Shi , Eng. Appl. Comp. Fluid Mech. 2023, 17, 2177350.

[186] J. Zhou , Z. Peng , I. Papautsky , Microsyst. Nanoeng. 2020, 6, 105. 34567714 10.1038/s41378-020-00217-y PMC8433405

[187] E. S. Asmolov , J. Fluid Mech. 1999, 381, 63.

[188] L. Zeng , S. Balachandar , P. Fischer , J. Fluid Mech. 2005, 536, 1.

[189] P. G. Saffman , J. Fluid Mech. 1965, 22, 385.

[190] S. I. Rubinow , J. B. Keller , J. Fluid Mech. 1961, 11, 447.

[191] J. M. Martel , M. Toner , Annu. Rev. Biomed. Eng. 2014, 16, 371. 24905880 10.1146/annurev-bioeng-121813-120704 PMC4467210

[192] X. Li , Y. Yang , S. C. Villareal , K. Griffin , D. Pappas , Analyst 2022, 147, 4536. 36098233 10.1039/d2an01310j

[193] J.‐P. Matas , J. F. Morris , É. Guazzelli , J. Fluid Mech. 2004, 515, 171.

[194] D. Di Carlo , Lab Chip 2009, 9, 3038. 19823716 10.1039/b912547g

[195] J. A. Schonberg , E. J. Hinch , J. Fluid Mech. 1989, 203, 517.

[196] H. Shi , Y. Zhao , Z. Liu , Sens. Actuator B‐Chem. 2020, 321, 128503.

[197] E. Ghazimirsaeed , M. Madadelahi , M. Dizani , A. Shamloo , Langmuir 2021, 37, 5118. 33877832 10.1021/acs.langmuir.0c03662

[198] D. H. Yoon , J. B. Ha , Y. K. Bahk , T. Arakawa , S. Shoji , J. S. Go , Lab Chip 2009, 9, 87. 19209339 10.1039/b809123d

[199] H. Jeon , T. Kwon , J. Yoon , J. Han , Lab Chip 2022, 22, 272. 34931631 10.1039/d1lc00995h

[200] S. Ramya , S. P. Kumar , G. D. Ram , D. Lingaraja , Microfluid. Nanofluid 2022, 26, 95.

[201] K. Akbarnataj , S. Maleki , M. Rezaeian , M. Haki , A. Shamloo , Talanta 2023, 254, 124125. 36462283 10.1016/j.talanta.2022.124125

[202] C. Wang , Y. Chen , X. Gu , X. Zhang , C. Gao , L. Dong , S. Zheng , S. Feng , N. Xiang , Electrophoresis 2021, 43, 464. 34611912 10.1002/elps.202100232

[203] S. Ebrahimi , M. Alishiri , A. Shamloo , E. Pishbin , P. Hemmati , S. Seifi , H. Shaygani , Sens. Actuator A‐Phys. 2023, 358, 114432.

[204] Z. Zhu , D. Wu , S. Li , Y. Han , N. Xiang , C. Wang , Z. Ni , Anal. Chim. Acta 2021, 1143, 306. 33384126 10.1016/j.aca.2020.11.001

[205] H. Ren , Z. Zhu , N. Xiang , H. Wang , T. Zheng , H. An , N.‐T. Nguyen , J. Zhang , Sens. Actuator B‐Chem. 2021, 337, 129758.

[206] A. J. Mach , J. H. Kim , A. Arshi , S. C. Hur , D. Di Carlo , Lab Chip 2011, 11, 2827. 21804970 10.1039/c1lc20330d

[207] Z. Zhu , H. Ren , D. Wu , Z. Ni , N. Xiang , Microsyst. Nanoeng. 2024, 10, 36. 38482464 10.1038/s41378-024-00661-0 PMC10933397

[208] E. Sollier , D. E. Go , J. Che , D. R. Gossett , S. O'Byrne , W. M. Weaver , N. Kummer , M. Rettig , J. Goldman , N. Nickols , S. McCloskey , R. P. Kulkarni , D. Di Carlo , Lab Chip 2014, 14, 63. 24061411 10.1039/c3lc50689d

[209] D. Jiang , C. Ni , W. Tang , D. Huang , N. Xiang , Biomicrofluidics 2021, 15, 041501. 34262632 10.1063/5.0058732 PMC8254650

[210] R. Khojah , R. Stoutamore , D. Di Carlo , Lab Chip 2017, 17, 2542. 28613306 10.1039/c7lc00355b

[211] X. Wang , J. Zhou , I. Papautsky , Biomicrofluidics 2013, 7, 044119. 24404052 10.1063/1.4818906 PMC3765293

[212] S. C. Hur , A. J. Mach , D. Di Carlo , Biomicrofluidics 2011, 5, 022206. 21918676 10.1063/1.3576780 PMC3171489

[213] P. Paiè , J. Che , D. Di Carlo , Microfluid. Nanofluid. 2017, 21, 104.

[214] X. Wang , I. Papautsky , Lab Chip 2015, 15, 1350. 25590954 10.1039/c4lc00803k

[215] X. Wang , X. Yang , I. Papautsky , Technology 2016, 04, 88.

[216] C. Renier , E. Pao , J. Che , H. E. Liu , C. A. Lemaire , M. Matsumoto , M. Triboulet , S. Srivinas , S. S. Jeffrey , M. Rettig , R. P. Kulkarni , D. Di Carlo , E. Sollier‐Christen , npjP. Oncol 2017, 1, 15. 10.1038/s41698-017-0015-0 PMC5859469 29872702

[217] A. Karimi , M. Sattari‐Najafabadi , Heliyon 2023, 9, 20380. 10.1016/j.heliyon.2023.e20380 PMC10539965 37780775

[218] J. H. Shin , M. G. Lee , S. Choi , J.‐K. Park , RSC Adv. 2014, 4, 39140.

[219] A. Mihandoust , N. Maleki‐Jirsaraei , S. Rouhani , S. Safi , M. Alizadeh , Electrophoresis 2020, 41, 353. 32012295 10.1002/elps.201900436

[220] Y. Huang , S. Yu , S. Chao , L. Wu , M. Tao , B. Situ , X. Ye , Y. Zhang , S. Luo , W. Chen , X. Jiang , G. Guan , L. Zheng , Lab Chip 2020, 20, 4342. 33155006 10.1039/d0lc00895h

[221] A. Behera , S. Mane , V. Hemadri , S. Bhand , S. Tripathi , IEEE Sens. Lett. 2023, 7, 1.37529707

[222] A. Abdulla , T. Zhang , K. Z. Ahmad , S. Li , J. Lou , X. Ding , Anal. Chem. 2020, 92, 16170. 33232155 10.1021/acs.analchem.0c03920

[223] A. H. Raffiee , A. M. Ardekani , S. Dabiri , J. Non‐Newton , Fluid Mech 2019, 272, 104166.

[224] X. Lu , C. Liu , G. Hu , X. Xuan , J. Colloid Interface Sci. 2017, 500, 182. 28412635 10.1016/j.jcis.2017.04.019

[225] X. Lu , X. Xuan , Anal. Chem. 2015, 87, 11523. 26505113 10.1021/acs.analchem.5b03321

[226] X. Lu , X. Xuan , Anal. Chem. 2015, 87, 6389. 26005774 10.1021/acs.analchem.5b01432

[227] C. Liu , C. Xue , X. Chen , L. Shan , Y. Tian , G. Hu , Anal. Chem. 2015, 87, 6041. 25989347 10.1021/acs.analchem.5b00516

[228] A. M. Leshansky , A. Bransky , N. Korin , U. Dinnar , Phys. Rev. Lett. 2007, 98, 234501. 17677908 10.1103/PhysRevLett.98.234501

[229] Y. Chen , L. Jiang , X. Zhang , Z. Ni , N. Xiang , Anal. Chem. 2023, 95, 18180. 38018866 10.1021/acs.analchem.3c03792

[230] V. Tirtaatmadja , G. H. McKinley , J. J. Cooper‐White , Phys. Fluids 2006, 18, 043101.

[231] C.‐S. Gan , Z.‐Z. Tian , L. Liu , L.‐L. Fan , L. Zhao , Microfluid. Nanofluid 2024, 28, 38.

[232] D. F. James , Annu. Rev. Fluid Mech. 2009, 41, 129.

[233] C. Liu , J. Guo , F. Tian , N. Yang , F. Yan , Y. Ding , J. Wei , G. Hu , G. Nie , J. Sun , ACS Nano 2017, 11, 6968. 28679045 10.1021/acsnano.7b02277

[234] E. Cho , J.‐a. Kim , M. K. Aslan , Y. Meng , S. Stavrakis , A. deMello , Sens. Actuator B‐Chem 2024, 414, 135892.

[235] D. Li , X. Xuan , Phys. Fluids 2023, 35, 092013.

[236] H. Lim , J. Nam , S. Shin , Microfluid. Nanofluid 2014, 17, 683.

[237] B. Ha , J. Park , G. Destgeer , J. H. Jung , H. J. Sung , Anal. Chem. 2016, 88, 4205. 27049167 10.1021/acs.analchem.6b00710

[238] K. Devanand , J. C. Selser , Macromolecules 1991, 24, 5943.

[239] F. Tian , L. Cai , J. Chang , S. Li , C. Liu , T. Li , J. Sun , Lab Chip 2018, 18, 3436. 30328446 10.1039/c8lc00700d

[240] F. Tian , W. Zhang , L. Cai , S. Li , G. Hu , Y. Cong , C. Liu , T. Li , J. Sun , Lab Chip 2017, 17, 3078. 28805872 10.1039/c7lc00671c

[241] Y. Lu , W. C. d. Vries , N. J. Overeem , X. Duan , H. Zhang , H. Zhang , W. Pang , B. J. Ravoo , J. Huskens , Angew. Chem.‐Int. Edit. 2018, 58, 159. 10.1002/anie.201810181 PMC6391938 30417518

[242] M. Asghari , X. Cao , B. Mateescu , D. van Leeuwen , M. K. Aslan , S. Stavrakis , A. J. deMello , ACS Nano 2019, 14, 422. 31794192 10.1021/acsnano.9b06123

[243] M. K. D. Manshadi , M. Mohammadi , L. K. Monfared , A. Sanati‐Nezhad , Biotechnol. Bioeng. 2019, 117, 580. 31654394 10.1002/bit.27211

[244] S. Hettiarachchi , L. Ouyang , H. Cha , H. H. W. B. Hansen , H. An , N.‐T. Nguyen , J. Zhang , Nanoscale 2024, 16, 3560. 38289397 10.1039/d3nr05410a

[245] B. H. Wunsch , K. Y. Hsieh , S. C. Kim , M. Pereira , S. Lukashov , C. Scerbo , J. M. Papalia , E. A. Duch , G. Stolovitzky , S. M. Gifford , J. T. Smith , Adv. Mater. Technol. 2021, 6, 2001083.

[246] R. Vernekar , T. Kruger , K. Loutherback , K. Morton , D. W. Inglis , Lab Chip 2017, 17, 3318. 28861573 10.1039/c7lc00785j

[247] Z. Liu , Y. Huang , W. Liang , J. Bai , H. Feng , Z. Fang , G. Tian , Y. Zhu , H. Zhang , Y. Wang , A. Liu , Y. Chen , Lab Chip 2021, 21, 2881. 34219135 10.1039/d1lc00360g

[248] J.‐c. Hyun , J. Hyun , S. Wang , S. Yang , Sep. Purif. Technol. 2017, 172, 258.

[249] S. Du , S. Shojaei‐Zadeh , G. Drazer , Soft Matter 2017, 13, 7649. 28990019 10.1039/c7sm01510k

[250] M. Xavier , S. H. Holm , J. P. Beech , D. Spencer , J. O. Tegenfeldt , R. O. C. Oreffo , H. Morgan , Lab Chip 2019, 19, 513. 30632599 10.1039/c8lc01154k

[251] S. L. Feng , A. M. Skelley , A. G. Anwer , G. Liu , D. W. Inglis , Biomicrofluidics 2017, 11, 024121. 28503245 10.1063/1.4981014 PMC5409848

[252] A. Hochstetter , R. Vernekar , R. H. Austin , H. Becker , J. P. Beech , D. A. Fedosov , G. Gompper , S. C. Kim , J. T. Smith , G. Stolovitzky , J. O. Tegenfeldt , B. H. Wunsch , K. K. Zeming , T. Kruger , D. W. Inglis , ACS Nano 2020, 14, 10784. 32844655 10.1021/acsnano.0c05186

[253] Y. Wang , J. Wang , Y. Wu , J. Dong , J. Chem. Technol. Biotechnol. 2021, 96, 2228.

[254] B. M. Dincau , A. Aghilinejad , X. Chen , S. Y. Moon , J.‐H. Kim , Microfluid. Nanofluid. 2018, 22, 137.

[255] K. Torres‐Castro , J. Jarmoshti , L. Xiao , A. Rane , A. Salahi , L. Jin , X. Li , F. Caselli , C. Honrado , N. S. Swami , Adv. Mater. Technol. 2023, 8, 2201463. 37706194 10.1002/admt.202201463 PMC10497222

[256] E. Pariset , C. Pudda , F. Boizot , N. Verplanck , J. Berthier , A. Thuaire , V. Agache , Small 2017, 13, 1701901. 10.1002/smll.201701901 28783259

[257] T. G. Kuznetsova , M. N. Starodubtseva , N. I. Yegorenkov , S. A. Chizhik , R. I. Zhdanov , Micron 2007, 38, 824. 17709250 10.1016/j.micron.2007.06.011

[258] B. R. Long , M. Heller , J. P. Beech , H. Linke , H. Bruus , J. O. Tegenfeldt , Phys. Rev. E 2008, 78, 046304. 10.1103/PhysRevE.78.046304 18999523

[259] N. Kihara , D. Kuboyama , D. Onoshima , K. Ishikawa , H. Tanaka , N. Ozawa , T. Hase , R. Koguchi , H. Yukawa , H. Odaka , Y. Hasegawa , Y. Baba , M. Hori , Jpn. J. Appl. Phys. 2018, 57, 037001.

[260] Y. Tang , J. Shi , S. Li , L. Wang , Y. E. Cayre , Y. Chen , Sci. Rep. 2014, 4, 6052. 25116599 10.1038/srep06052 PMC7365311

[261] X. Li , W. Chen , G. Liu , W. Lu , J. Fu , Lab Chip 2014, 14, 2565. 24895109 10.1039/c4lc00350k PMC4106416

[262] X. Fan , C. Jia , J. Yang , G. Li , H. Mao , Q. Jin , J. Zhao , Biosens. Bioelectron. 2015, 71, 380. 25950932 10.1016/j.bios.2015.04.080

[263] S. Zheng , H. Lin , J.‐Q. Liu , M. Balic , R. Datar , R. J. Cote , Y.‐C. Tai , J. Chromatogr. A 2007, 1162, 154. 17561026 10.1016/j.chroma.2007.05.064

[264] S. J. Tan , L. Yobas , G. Y. H. Lee , C. N. Ong , C. T. Lim , Biomed. Microdevices 2009, 11, 883. 19387837 10.1007/s10544-009-9305-9

[265] D. Onoshima , T. Hase , N. Kihara , D. Kuboyama , H. Tanaka , N. Ozawa , H. Yukawa , M. Sato , K. Ishikawa , Y. Hasegawa , M. Ishii , M. Hori , Y. Baba , ACS Meas. Sci. Au 2022, 3, 113. 37090261 10.1021/acsmeasuresciau.2c00057 PMC10120030

[266] Y. Cheng , X. Ye , Z. Ma , S. Xie , W. Wang , Biomicrofluidics 2016, 10, 014118. 26909124 10.1063/1.4941985 PMC4752536

[267] M. Du , Z. Ma , X. Ye , Z. Zhou , Sci. China‐Technol. Sci. 2013, 56, 1047.

[268] D. L. Adams , P. Zhu , O. V. Makarova , S. S. Martin , M. Charpentier , S. Chumsri , S. Li , P. Amstutz , C.‐M. Tang , RSC Adv. 2014, 4, 4334. 10.1039/C3RA46839A PMC4299665 25614802

[269] Y. Quan , Z. Zhu , D. Tang , S. Zhu , C. Wang , K. Chen , Z. Ni , Sep. Purif. Technol. 2022, 296, 121349.

[270] H. Luo , F. Liang , W. Wang , X. Huang , Z. Mao , L. Wang , J. Shi , J. Peng , Y. Chen , Sens. Actuator B‐Chem. 2024, 398, 134720.

[271] J. S. Kuo , Y. Zhao , P. G. Schiro , L. Ng , D. S. W. Lim , J. P. Shelby , D. T. Chiu , Lab Chip 2010, 10, 837. 20379567 10.1039/b922301k

[272] T. Morijiri , S. Sunahiro , M. Senaha , M. Yamada , M. Seki , Microfluid. Nanofluid. 2011, 11, 105.

[273] J. Takagi , M. Yamada , M. Yasuda , M. Seki , Lab Chip 2005, 5, 778. 15970972 10.1039/b501885d

[274] M. Yamada , M. Nakashima , M. Seki , Anal. Chem. 2004, 76, 5465. 15362908 10.1021/ac049863r

[275] A. L. Vig , A. Kristensena , Appl. Phys. Lett. 2008, 93, 203507.

[276] S. Wang , Z. Liu , S. Wu , H. Sun , W. Zeng , J. Wei , Z. Fan , Z. Sui , L. Liu , X. Pan , Electrophoresis 2021, 42, 2223. 33938005 10.1002/elps.202000325

[277] H. W. Nho , T. H. Yoon , Lab Chip 2013, 13, 773. 23340906 10.1039/c2lc41154g

[278] H. W. Nho , N. Yang , J. Song , J. S. Park , T. H. Yoon , Sens. Actuator B‐Chem. 2017, 249, 131.

[279] S. Wang , Q. Xu , Z. Zhang , S. Chen , Y. Jiang , Z. Feng , D. Wang , X. Jiang , Lab Chip 2023, 23, 4324. 37702391 10.1039/d3lc00473b

[280] X. Lu , X. Xuan , Anal. Chem. 2015, 87, 4560. 25837725 10.1021/acs.analchem.5b00752

[281] J. T. W. Berendsen , J. C. T. Eijkel , A. M. Wetzels , L. I. Segerink , Microsyst. Nanoeng. 2019, 5, 24. 31123596 10.1038/s41378-019-0068-z PMC6527678

[282] T. Kaya , H. Koser , Phys. Rev. Lett. 2009, 103, 138103. 19905544 10.1103/PhysRevLett.103.138103

[283] S. Ebrahimi , Z. Rostami , M. Alishiri , A. Shamloo , S. M. A. Hoseinian , Phys. Fluids 2023, 35, 121906.

[284] T. Peng , J. Qiang , S. Yuan , Phys. Fluids 2023, 35, 082009.

[285] M. Aghaamoo , A. Aghilinejad , X. Chen , J. Xu , Electrophoresis 2019, 40, 1486. 30740752 10.1002/elps.201800459

[286] J. Cai , B. Chen , M. He , G. Yuan , B. Hu , Anal. Chem. 2024, 96, 14222. 39159467 10.1021/acs.analchem.4c02876

[287] D. Huang , N. Xiang , Lab Chip 2021, 21, 1409. 33605279 10.1039/d0lc01223h

[288] M. Rahmati , X. Chen , Biomed. Microdevices 2021, 23, 49. 34581876 10.1007/s10544-021-00587-8

[289] K. K. Zeming , N. V. Thakor , Y. Zhang , C. H. Chen , Lab Chip 2016, 16, 75. 26575003 10.1039/c5lc01051a

[290] A. H. K. Ashkezari , M. Dizani , A. Shamloo , Acta Mech. 2022, 233, 1881.

[291] B. Çetin , M. B. Özer , E. Çağatay , S. Büyükkoçak , Biomicrofluidics 2016, 10, 014112. 26865905 10.1063/1.4940431 PMC4733080

[292] X. J. Hu , H. L. Liu , Y. X. Jin , L. Liang , D. M. Zhu , X. Q. Zhu , S. S. Guo , F. L. Zhou , Y. Yang , Lab Chip 2018, 18, 3405. 30357194 10.1039/c8lc00911b

[293] Y. Xu , B. Chen , M. He , Z. Cui , B. Hu , Anal. Chem. 2023, 95, 14061. 37677145 10.1021/acs.analchem.3c02680

[294] V. Calero , R. Fernández‐Mateo , H. Morgan , P. García‐Sánchez , A. Ramos , J. Chromatogr. A 2023, 1706. 10.1016/j.chroma.2023.464240 37544238

[295] J. P. Beech , K. Keim , B. D. Ho , C. Guiducci , J. O. Tegenfeldt , Adv. Mater. Technol. 2019, 4, 1900339.

[296] K. K. Zeming , T. Salafi , S. Shikha , Y. Zhang , Nat. Commun. 2018, 9, 1254. 29593276 10.1038/s41467-018-03596-z PMC5871788

[297] Z. G. Soroush , K. K. Vahid , M. Masoud , Chem. Eng. Process. 2024, 201, 109803.

[298] M. Tayebi , D. Yang , D. J. Collins , Y. Ai , Nano Lett. 2021, 21, 6835. 34355908 10.1021/acs.nanolett.1c01827

[299] R. Derakhshan , A. Mahboubidoust , A. Ramiar , Chem. Eng. Process. 2021, 167, 108544.

[300] H. Fang , M. Liu , W. Jiang , Appl. Biochem. Biotechnol. 2022, 195, 3109. 36542270 10.1007/s12010-022-04272-1

[301] L. Ding , X. Liu , Z. Zhang , L.‐e. Liu , S. He , Y. Wu , C. Y. Effah , R. Yang , A. Zhang , W. Chen , M. Yarmamat , L. Qu , X. Yang , Y. Wu , Lab Chip 2023, 23, 1694. 36789765 10.1039/d2lc00996j

[302] L. Wang , R. Wang , H. Wang , M. Slavik , H. Wei , Y. Li , Anal. Biochem. 2017, 533, 34. 28645756 10.1016/j.ab.2017.06.010

[303] F. Huang , H. Zhang , L. Wang , W. Lai , J. Lin , Biosens. Bioelectron. 2018, 100, 583. 29032045 10.1016/j.bios.2017.10.005

[304] Q. Zhang , T. Yin , R. Xu , W. Gao , H. Zhao , J. G. Shapter , K. Wang , Y. Shen , P. Huang , G. Gao , Y. Wu , D. Cui , Nanoscale 2017, 9, 13592. 28875998 10.1039/c7nr04914e

[305] M. Khorrami , M. Mahmoudi , S. S. Shobeiri , M. Moghadam , M. Sankian , Appl. Biochem. Microbiol. 2023, 59, 706.

[306] K. Takemura , J. Lee , T. Suzuki , T. Hara , F. Abe , E. Y. Park , Sens. Actuator B‐Chem 2019, 296, 126672.

[307] Y. Wang , Y. Li , R. Wang , M. Wang , J. Lin , J. Sep. Sci. 2017, 40, 1540. 28139889 10.1002/jssc.201601379

[308] W.‐P. Chou , H.‐M. Wang , J.‐H. Chang , T.‐K. Chiu , C.‐H. Hsieh , C.‐J. Liao , M.‐H. Wu , Sens. Actuator B‐Chem 2017, 241, 245.

[309] J. Cebricos , R. Hoptowit , S. Jun , LWT‐Food Sci. Technol. 2017, 80, 185.

[310] L. D'Amico , N. J. Ajami , J. A. Adachi , P. R. C. Gascoyne , J. F. Petrosino , Lab Chip 2017, 17, 1340. 28276545 10.1039/c6lc01277a PMC5894507

[311] F. R. Madiyar , S. L. Haller , O. Farooq , S. Rothenburg , C. Culbertson , J. Li , Electrophoresis 2017, 38, 1515. 28211116 10.1002/elps.201600436

[312] Y. Chen , M. Wu , L. Ren , J. Liu , P. H. Whitley , L. Wang , T. J. Huang , Lab Chip 2016, 16, 3466. 27477388 10.1039/c6lc00682e PMC5010861

[313] Y. Gu , C. Chen , Z. Wang , P.‐H. Huang , H. Fu , L. Wang , M. Wu , Y. Chen , T. Gao , J. Gong , J. Kwun , G. M. Arepally , T. J. Huang , Lab Chip 2019, 19, 394. 30631874 10.1039/c8lc00527c PMC6366625

[314] C. Richard , A. Fakhfouri , M. Colditz , F. Striggow , R. Kronstein‐Wiedemann , T. Tonn , M. Medina‐Sánchez , O. G. Schmidt , T. Gemming , A. Winkler , Lab Chip 2019, 19, 4043. 31723953 10.1039/c9lc00804g

[315] E. J. Fong , A. C. Johnston , T. Notton , S.‐Y. Jung , K. A. Rose , L. S. Weinberger , M. Shusteff , Analyst 2014, 139, 1192. 24448925 10.1039/c4an00034j

[316] K. Cai , S. Mankar , T. Ajiri , K. Shirai , T. Yotoriyama , Lab Chip 2021, 21, 3112. 34286793 10.1039/d1lc00298h

[317] T. Zhang , A. K. Cain , L. Semenec , L. Liu , Y. Hosokawa , D. W. Inglis , Y. Yalikun , M. Li , Anal. Chem. 2023, 95, 2561. 36656064 10.1021/acs.analchem.2c05084

[318] T. Zhang , A. K. Cain , L. Semenec , J. V. Pereira , Y. Hosokawa , Y. Yalikun , M. Li , Sens. Actuator B‐Chem. 2023, 390, 133918.

[319] H. Tang , J. Niu , X. Pan , H. Jin , S. Lin , D. Cui , J. Chromatogr. A 2022, 1679, 463384. 35940060 10.1016/j.chroma.2022.463384

[320] V. Varmazyari , H. Ghafoorifard , H. Habibiyan , M. Ebrahimi , S. Ghafouri‐Fard , J. Mol. Liq. 2022, 349, 118192. 10.1038/s41598-022-16286-0 PMC9287561 35840699

[321] R. Bhattacharjee , R. Kumar , F. Al‐Turjman , Cogn. Comput. 2021, 14, 1660.

[322] J. T. Smith , B. H. Wunsch , N. Dogra , M. E. Ahsen , K. Lee , K. K. Yadav , R. Weil , M. A. Pereira , J. V. Patel , E. A. Duch , J. M. Papalia , M. F. Lofaro , M. Gupta , A. K. Tewari , C. Cordon‐Cardo , G. Stolovitzky , S. M. Gifford , Lab Chip 2018, 18, 3913. 30468237 10.1039/c8lc01017j

[323] J. A. Hernández‐Castro , K. Li , A. Meunier , D. Juncker , T. Veres , Lab Chip 2017, 17, 1960. 28443860 10.1039/c6lc01525e

[324] M. Podenphant , N. Ashley , K. Koprowska , K. U. Mir , M. Zalkovskij , B. Bilenberg , W. Bodmer , A. Kristensen , R. Marie , Lab Chip 2015, 15, 4598. 26510401 10.1039/c5lc01014d

[325] H.‐S. Moon , K. Kwon , S.‐I. Kim , H. Han , J. Sohn , S. Lee , H.‐I. Jung , Lab Chip 2011, 11, 1118. 21298159 10.1039/c0lc00345j

[326] J. Yao , J. Chen , X. Cao , H. Dong , Talanta 2019, 196, 546. 30683404 10.1016/j.talanta.2018.12.059

[327] R. J. Gillams , V. Calero , R. Fernandez‐Mateo , H. Morgan , Lab Chip 2022, 22, 3869. 36065949 10.1039/d2lc00583b

[328] T. Jung , Y.‐R. Yun , J. Bae , S. Yang , Anal. Chim. Acta 2021, 1173, 338696. 34172153 10.1016/j.aca.2021.338696

[329] D. J. Guckenberger , T. E. de Groot , A. M. D. Wan , D. J. Beebe , E. W. K. Young , Lab Chip 2015, 15, 2364. 25906246 10.1039/c5lc00234f PMC4439323

[330] K. Sugioka , J. Xu , D. Wu , Y. Hanada , Z. Wang , Y. Cheng , K. Midorikawa , Lab Chip 2014, 14, 3447. 25012238 10.1039/c4lc00548a

[331] Y. Qin , J. E. Kreutz , T. Schneider , G. S. Yen , E. S. Shah , L. Wu , D. T. Chiu , Lab Chip 2022, 22, 4729. 36367074 10.1039/d2lc00857b PMC9691590

[332] U. N. Lee , X. Su , D. J. Guckenberger , A. M. Dostie , T. Zhang , E. Berthier , A. B. Theberge , Lab Chip 2018, 18, 496. 29309079 10.1039/c7lc01052d PMC5790604

[333] A. Khademhosseini , K. Y. S. S. Jon , G. Eng , J. Yeh , G.‐J. Chen , R. Langer , Anal. Chem. 2004, 76, 3675. 15228340 10.1021/ac035415s

[334] A. V. Nielsen , M. J. Beauchamp , G. P. Nordin , A. T. Woolley , Annu. Rev. Anal. Chem. 2020, 13, 45. 10.1146/annurev-anchem-091619-102649 PMC7282950 31821017

[335] J. Jiang , X. Cui , Y. Huang , D. Yan , B. Wang , Z. Yang , M. Chen , J. Wang , Y. Zhang , G. Liu , C. Zhou , S. Cui , J. Ni , F. Yang , D. Cui , Nano Biomed. Eng. 2024, 16, 152.

[336] Y. Wu , J. Zhang , W. He , C. Li , Y. Wang , Nano Biomed. Eng. 2023, 15, 199.

[337] W. Chen , Y. Xu , D. Yang , P. Wang , Y. Xu , J. Zhu , D. Cui , Nano Biomed. Eng. 2022, 14, 71.

[338] W. Li , Z. Yao , T. Ma , Z. Ye , K. He , L. Wang , H. Wang , Y. Fu , X. Xu , Adv. Colloid Interface Sci. 2024, 332. 10.1016/j.cis.2024.103276 39146580

[339] N. Gulbahce , M. J. M. Magbanua , R. Chin , M. R. Agarwal , X. Luo , J. Liu , D. M. Hayden , Q. Mao , S. Ciotlos , Z. Li , Y. Chen , X. Chen , Y. Li , R. Y. Zhang , K. Lee , R. Tearle , E. Park , S. Drmanac , H. S. Rugo , J. W. Park , R. Drmanac , B. A. Peters , Cancer Res. 2017, 77, 4530. 28811315 10.1158/0008-5472.CAN-17-0688

[340] L. Wang , W. Asghar , U. Demirci , Y. Wan , Nano Today 2013, 8, 374. 10.1016/j.nantod.2013.07.001 PMC4059613 24944563

[341] E. E. Tsur , Annu. Rev. Biomed. Eng. 2020, 22, 285. 32343907 10.1146/annurev-bioeng-082219-033358

[342] D. Stoecklein , D. Di Carlo , Anal. Chem. 2019, 91, 296. 30501182 10.1021/acs.analchem.8b05042

[343] C. N. Baroud , F. Gallaire , R. Dangla , Lab Chip 2010, 10, 2032. 20559601 10.1039/c001191f

[344] S. L. Anna , Annu. Rev. Fluid Mech. 2016, 48, 285.

[345] H. Fallahi , J. Zhang , J. Nicholls , H.‐P. Phan , N.‐T. Nguyen , Anal. Chem. 2020, 92, 12473. 32786464 10.1021/acs.analchem.0c02294

[346] R. Su , F. Wang , M. C. McAlpine , Lab Chip 2023, 23, 1279. 36779387 10.1039/d2lc01177h

[347] S. Waheed , J. M. Cabot , N. P. Macdonald , T. Lewis , R. M. Guijt , B. Paull , M. C. Breadmore , Lab Chip 2016, 16, 1993. 27146365 10.1039/c6lc00284f

[348] L. Wu , Z. Dong , Adv. Mater. 2023, 35. 10.1002/adma.202300903 37147788

[349] S. R. Shin , T. Kilic , Y. S. Zhang , H. Avci , N. Hu , D. Kim , C. Branco , J. Aleman , S. Massa , A. Silvestri , J. Kang , A. Desalvo , M. A. Hussaini , S. K. Chae , A. Polini , N. Bhise , M. A. Hussain , H. Lee , M. R. Dokmeci , A. Khademhosseini , Adv. Sci. 2017, 4. 10.1002/advs.201600522 PMC5441508 28546915

[350] K. Kadimisetty , S. Malla , J. F. Rusling , ACS Sens. 2017, 2, 670. 28723166 10.1021/acssensors.7b00118 PMC5535808

[351] Y. Yang , Y. Ohtake , T. Yatagawa , H. Suzuki , Virtual Phys. Prototyp. 2021, 17, 33.

[352] G. Gonzalez , I. Roppolo , C. F. Pirri , A. Chiappone , Addit. Manuf. 2022, 55,

[353] D. McIntyre , A. Lashkaripour , P. Fordyce , D. Densmore , Lab Chip 2022, 22, 2925. 35904162 10.1039/d2lc00254j PMC9361804

[354] J. Zhang , D. Yuan , Q. Zhao , S. Yan , S.‐Y. Tang , S. H. Tan , J. Guo , H. Xia , N.‐T. Nguyen , W. Li , Sens. Actuator B‐Chem. 2018, 267, 14.

